# Allele-Specific Methylation Occurs at Genetic Variants Associated with Complex Disease

**DOI:** 10.1371/journal.pone.0098464

**Published:** 2014-06-09

**Authors:** John N. Hutchinson, Towfique Raj, Jes Fagerness, Eli Stahl, Fernando T. Viloria, Alexander Gimelbrant, Johanna Seddon, Mark Daly, Andrew Chess, Robert Plenge

**Affiliations:** 1 Division of Rheumatology, Immunology and Allergy, Brigham and Women's Hospital, Boston, Massachusetts, United States of America; 2 Department of Biostatistics, Harvard School of Public Health, Boston, Massachusetts, United States of America; 3 Broad Institute, Cambridge, Massachusetts, United States of America; 4 Division of Genetics, Department of Medicine, Brigham and Women's Hospital, Boston, Massachusetts, United States of America; 5 Center for Human Genetic Research, Massachusetts General Hospital, Boston, Massachusetts, United States of America; 6 Department of Genetics, Harvard Medical School, Boston, Massachusetts, United States of America; 7 Department of Cancer Biology, Dana-Farber Cancer Institute, Boston, Massachusetts, United States of America; 8 Department of Ophthalmology, Tufts University School of Medicine, Boston, Massachusetts, United States of America; 9 Ophthalmic Epidemiology and Genetics Service, Tufts Medical Center, Boston, Massachusetts, United States of America; 10 Department of Developmental and Regenerative Biology, Fishberg Department of Neuroscience, Department of Genetics and Genomic Sciences, Friedman Brain Institute, Mount Sinai School of Medicine, New York, New York, United States of America; Ohio State University Medical Center, United States of America

## Abstract

We hypothesize that the phenomenon of allele-specific methylation (ASM) may underlie the phenotypic effects of multiple variants identified by Genome-Wide Association studies (GWAS). We evaluate ASM in a human population and document its genome-wide patterns in an initial screen at up to 380,678 sites within the genome, or up to 5% of the total genomic CpGs. We show that while substantial inter-individual variation exists, 5% of assessed sites show evidence of ASM in at least six samples; the majority of these events (81%) are under genetic influence. Many of these cis-regulated ASM variants are also eQTLs in peripheral blood mononuclear cells and monocytes and/or in high linkage-disequilibrium with variants linked to complex disease. Finally, focusing on autoimmune phenotypes, we extend this initial screen to confirm the association of cis-regulated ASM with multiple complex disease-associated variants in an independent population using next-generation bisulfite sequencing. These four variants are implicated in complex phenotypes such as ulcerative colitis and AIDS progression disease (rs10491434), Celiac disease (rs2762051), Crohn's disease, IgA nephropathy and early-onset inflammatory bowel disease (rs713875) and height (rs6569648). Our results suggest cis-regulated ASM may provide a mechanistic link between the non-coding genetic changes and phenotypic variation observed in these diseases and further suggests a route to integrating DNA methylation status with GWAS results.

## Introduction

In recent years, Genome Wide Association Studies (GWASs) have unearthed thousands of disease associated DNA sequence variants. As the majority of these variants are non-coding, their functional roles have been difficult to identify. Recent evidence showing enrichment of expression Quantitative Trait Loci (eQTLs) within these uncategorized groups of variants [1] suggests they can affect phenotype by regulating gene expression levels, likely through their effects on regulatory mediators. Indeed, GWAS-derived variants are enriched for regulatory marks such as DNAse hypersensitivity [2] and various chromatin states [3], [4].

Evidence also suggests that DNA methylation [5], an epigenetic process that can regulate gene expression [6], may also mediate genetic variants' phenotypic effects [7]. While studies of DNA methylation have typically focused on either CpG islands or differentially methylated regions associated with genomic imprinting, studies [8]–[11] have demonstrated a novel type of differential methylation where the methylation mark is consistently associated with one allele. Often termed allele-specific methylation (or ASM), the phenomenon can be influenced to varying degrees by DNA sequence within a population, ranging from complete association of methylation and genotype (i.e. cis-regulated ASM) [9]–[11], to more stochastic associations, where either allele may be associated with the methylation mark. As ASM is associated with expression changes in nearby genes, this genetic control of cis-regulated differential methylation has the potential to affect phenotypic variation [10]–[13].

Here we investigate 1) genome-wide/population patterns of ASM, including genomic features of ASM regions; and 2) the overlap between genetic variants associated with complex phenotypes and genetic variants that control ASM to identify individual ASM loci associated with complex traits. To do so, we employ a recently developed method that utilizes single nucleotide polymorphism (SNP) genotyping arrays [8], [9] in an initial screen to systematically identify cis-regulated ASM in a population. We then verify a subset of these cis-regulated ASM regions in an independent population by targeted next-generation bisulfite sequencing. Our findings show that cis-regulated ASM can be associated with intergenic variants linked to both expression and phenotypic variation, suggesting it could provide a mechanistic link between the non-coding genetic changes and phenotypic variation observed in many GWA studies.

## Results

We performed an initial screen for ASM in whole blood using a microarray method based method and confirmed a small subset of these loci using next-generation bisulfite sequencing.

### Microarray Based Detection of Allele-Specific Methylation

**Figure 1:**
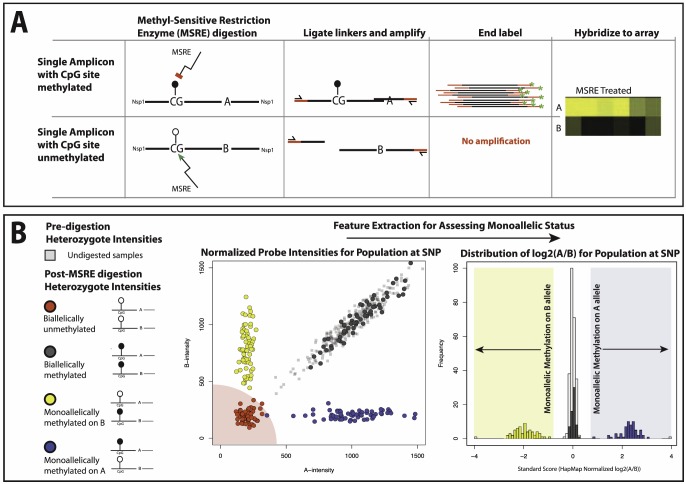
Microarray based detection of allele-specific methylation. **A)** A simplified representation of the Methyl-Sensitive Restriction Enzyme (MSRE) based Allele-Specific Methylation (ASM) assay. DNA is MSRE treated (left panels) and MSRE sites with methylated CpGs protected from digestion (upper panels, Allele-A) while its homologous chromosomal region with unmethylated CpGs are not (lower panels, Allele-B). The DNA is digested with StyI and NspI to form 200–1200 bp fragments, linkers ligated and DNA amplified to create amplicons that are hybridized to the array. Only regions with protected MSRE sites (methylated CpG) are amplified and can hybridize to show signal on the array (final panel). **B)** Bioinformatic detection of Allele-Specific Methylation (ASM) from Affymetrix SNP 6.0 arrays signals after MSRE digestion. In the scatter plot on the left, 4 different expected states after MSRE digest at a heterozygous region are compared to the typical distribution of probe intensities observed within the HapMap samples for the same MPR (here portrayed by light grey squares): biallelic methylation (dark grey circles), monoallelic A methylation (blue circles), monoallelic B methylation (yellow circles) and finally biallelic lack of methylation (red circles). The primary calling method relies on feature extraction by way of conversion of 2-dimensional A and B probe intensity data (scatter plot) from heterozygotes to log2(A/B) values and is compared against the typical log2(A/B) distribution observed for this MPR within the HapMap samples (histogram, light grey). Simply put, MPRs diverging from this distribution after MSRE treatment are called ASM. Using this method, biallelic unmethylated states have the potential to result in false positive ASM calls as any log2(A/B) value would be based on background noise, so are filtered out by removing MPRS with low total intensities (highlighted here with a red quarter-circle, for further information on how this filter was devised, see Figure S8).

The initial microarray screen was based on detecting “loss of heterozygosity” signals after amplicon ablation by methylation sensitive restriction enzyme (MSRE) based digestion at sites nearby one of the two alleles. Using Affymetrix SNP 6.0 arrays we looked for allelic ratio changes of these MSRE positive regions (MPRs) after MSRE digestion of genomic DNA derived from whole blood. We adjusted for both probe-specific and sample specific biases by adjusting for the variation observed for individual SNP probes within HapMap samples and the variation observed for MSRE negative regions (or MNRs) within an individual sample, respectively (Figure 1).

To lower the chance of erroneously calling ASM we filtered out MPRs with 1) poor genotype discrimination by allelic ratio and genotype calling rate in the HapMap samples, 2) non-ideal predicted amplicon size, and 3) predicted presence of MSRE modifying SNPs (Figure S1). After quality control filtering, our assay can theoretically examine a maximum of 380,678 MPRs in an individual, were all SNPs to be heterozygous in that individual. More plausibly, because any given individual is heterozygous for ∼25% of SNPs, our assay interrogates approximately 100,000 different MPRs per individual. These MPRs contain 1,278,397 MSRE sites in total; with each MSRE site assaying one CpG, a maximum of ∼5% of the total genomic CpGs are assayed. A final filter acted to remove MPRs with low overall intensities in individual samples as the allelic ratios of these MPRs are overly affected by background noise, leaving a median number of 58,173 MPRs available for assay in each individual.

Using this methodology, we examined ASM patterns in whole blood samples derived from 42 individuals from the National Academy of Sciences-National Research Council World War II Veteran Twin Registry. We were able to see ASM at multiple sites known to exhibit ASM (Figure S2). Of the 5 cis-regulated ASM sites and the 8 imprinted DMRs from Schalkwyk et al. [11] that we could assay, we found that 5/5 show ASM and 7/8 show some level of monoallelic methylation, respectively. The allele preferred for ASM in our study was consistent with that observed by Schalkwyk et al. Of the 297,333 SNPs with at least one heterozygote sample, 127,292 show at least one ASM event within the sample population. Our allele-specific methylation assay is based on empirical cutoffs that exclude 95% of MNRs (for more details, see Information S1), and as such would be expected to exhibit a 5% false positive rate when assessing MPRs. We observed candidate ASM levels that were significantly higher than this expected false positive rate of 5%, with a mean rate of 7.8% ASM (or 1.6 fold higher than the expected false positive rate) (Table S1).

### Population Characteristics of Allele-Specific Methylation Candidates

We proceeded to attempt to identify candidates for cis-regulated ASM in this population. The extent to which a given MPR can be assessed for ASM is dependent upon the number of individuals heterozygous for each SNP, the strength of the ASM event, and the background noise of ASM (which may be due to technical artifact or stochastic variation in methylation itself). For the purposes of our study, we limited our analysis to MPRs with at least 6 heterozygous individuals in order to obtain robust statistics for ASM. Of the 242,533 MPRs eligible for analysis, 126,488 (52%) showed zero ASM events and 116,045 (48%) had at least one ASM event.

**Figure 2:**
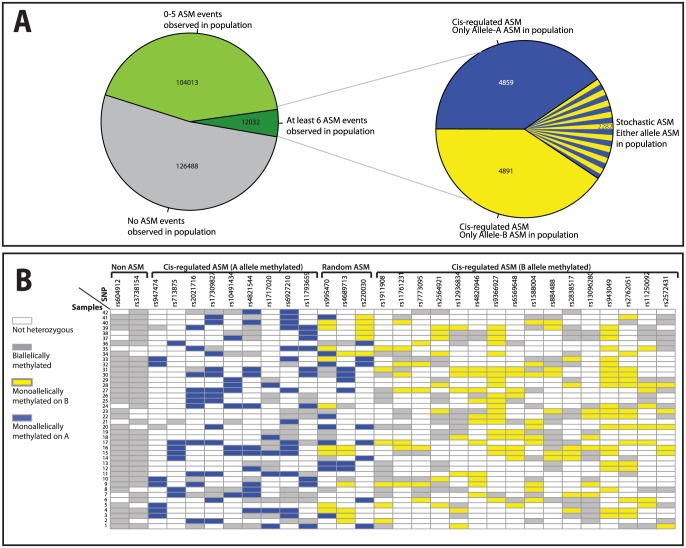
Types of allele-specific methylation candidates. Plots showing number of different categories of ASM candidates within the microarray study sample population. Of the 242533 MPRs for which there were at least 6 heterozygotes within the population (left pie chart) we detected some level of ASM in at least 116045 (left pie chart, green), of these we detected ASM in at least 6 samples in 12032 MPRs (left pie chart, dark green). Of these 12032 ASM MPRs, we detected cis-regulated ASM in 9750 MPRs (right pie chart, solid blue or solid yellow), and random or stochastic ASM in 2282 MPRs (right pie chart, mixed blue and yellow). Representation of patterns of allelic-choice in ASM within the microarray study sample population. ASM allelic choice is shown at 28 ASM and 2 non-ASM MPRs for the 42 samples in our initial microarray sample population. Non-heterozygous samples (white), samples with biallelic methylation (grey), and samples with ASM (blue and yellow) with methylation at either Allele-A (yellow) or Allele-A (blue) are shown. MPRs are organized in columns to show those determined to have no ASM (first two columns), cis-regulated ASM (both for Allele-A (3rd to 11th columns) and Allele-B (15th to final columns) or random ASM (12th to 14th columns).

To minimize the number of false positive ASM events, we focused on 12,032 MPRs with at least 6 ASM events. We picked this threshold, as it is the minimum number of samples necessary to yield a p-value of less than 0.05 in an exact binomial test. Of these candidate ASM events, 9,750 (∼81%) showed non-random allelic choice (i.e. for a given amplicon ASM events were on the A allele or all were on the B allele) and 2282 (∼19%) failed to reject the null hypothesis of random allelic choice (i.e.. we observed a mix between A allele and B allele ASM). That is, of the 242,533 MPRs eligible for analysis, 9,750 MPRs (or 4% of the total) showed evidence for cis-regulated ASM in our study population of 42 individuals (Figure 2A). Note that while MPRs with apparently random allelic choice may be imprinted, it is not possible to infer their prevalence from this statistic, as failure to reject the null hypothesis does not entail it's acceptance. Representative examples are shown in Figure 2B (Figure 2).

### Properties of Cis-Regulated ASM Candidates

To identify interesting subsets of these candidate cis-regulated ASM variants to study further, we examined 1) their genomic positions relative to genes 2) their status in two sets of Expression Quantitative Trait Loci (eQTLs) from appropriate cell types (monocytes and peripheral blood monocytes (PBMCs)) [14]–[16] and 3) their status in the set of GWAS variants curated by the National Human Genome Research Institute [17]. For the latter two steps, the candidate ASM SNP sets were pruned to remove SNPs in linkage disequilibrium (LD), yielding a mean of 8,147 cis-regulated ASM SNPs.

A majority of the candidate cis-regulated ASM associated SNPs were upstream or downstream of a gene (6113/9687 SNPs, or 63.1%) as opposed to within the gene body (defined as within the 5′UTR, 3′UTR, exon, or intron of an annotated gene) (Figure S3A). These non-genic candidate cis-regulated ASM SNPs were a median distance of ∼129 kb from the closest gene (Figure S3B). Multiple LD-pruned SNPs associated with candidate cis-regulated ASM were eQTLs in either monocytes (1752/8147) or PBMCs (906/8147). Finally, multiple candidate variants associated with cis-regulated ASM were in high LD (r^2^>0.80) with a variant drawn from the NHGRI GWAS catalog (average 90/8147 in 10 LD-pruned NHGRI GWAS SNP sets respectively) (Table 1). These GWAS variants were associated with a number of phenotypes, including such medically relevant phenotypes as autoimmunity, coronary heart disease, obesity, and type 2 diabetes. These findings provided us with a subset of candidate cis-regulated ASM loci that are biologically relevant for both disease and gene expression phenotypes (Table S2).

**Table 1:** Candidate cis-regulated ASM variants in phenotypically implicated SNPs.

		cis-regulated ASM		
		mean number	sd	%
	**LD pruned SNPs**	8147	323	100.0
**Subset that is also:**	**NHGRI GWAS SNP**	90	4	1.1
	**Monocyte eQTL**	1752	75	21.5
	**PBMC eQTL**	906	34	11.1

Mean number of candidate cis-regulated ASM variants in ten random LD pruned (ld<0.3) sets of candidate cis-regulated ASM SNPs that are also in high LD (LD>0.8) with a Genome Wide Association Study (GWAS) derived variant from the National Human Genome Research Institute (NHGRI) database or an eQTL in monocytes or peripheral blood monocytes (PBMCs), with accompanying standard deviations of the mean (sd).

### Confirmation in Independent Population

To independently confirm some of these candidate cis-regulated ASM loci, we performed a complementary assay (bisulfite sequencing) in an independent collection of healthy control subjects (n = 82). We prioritized regions for further investigation based on the following criteria: (1) the cis-regulated MPR must contain both a genetic variant and an MSRE site within a minimally sized target amplicon (n = 500 bp); (2) strength of ASM signal, defined as the percentage of heterozygous individuals with ASM; (3) association of the target SNP (or a SNP in high LD) with risk of an autoimmune disease, as determined from the NHGRI; and when possible (4) association of the target SNP (or a SNP in high LD) with an eQTL. We chose autoimmunity as a relevant phenotype because our ASM assays were done using whole blood samples, and many autoimmune risk alleles disrupt the function of genes expressed in whole blood. As controls, variants were also targeted based on their known ASM status (2) or lack of association with ASM (1) in our microarray data. After quality control filtering (see Methods), we examined 10 sites in 70 individuals; amplicons comprised two known ASM regions, one non-ASM region from our microarray studies and seven ASM regions found to be in high LD with GWAS variants.

As an initial step, to increase our ability to identify ASM associated variants in our set of targets, we combined the reads from all samples, and examined the association of methylation status with allele identity by chi-square analyses. Of the ten amplicons examined, two were positive controls (rs943049 and rs9366927) and seven (rs10491434, rs2021716, rs2564921, rs2762051, rs6569648, rs713875, rs884488) were variants in high LD with (or identical to) phenotypic variants that show evidence of ASM in our microarray data. One variant (rs3738154) showed no evidence of ASM in our microarray data and acted as a negative control.

**Figure 3:**
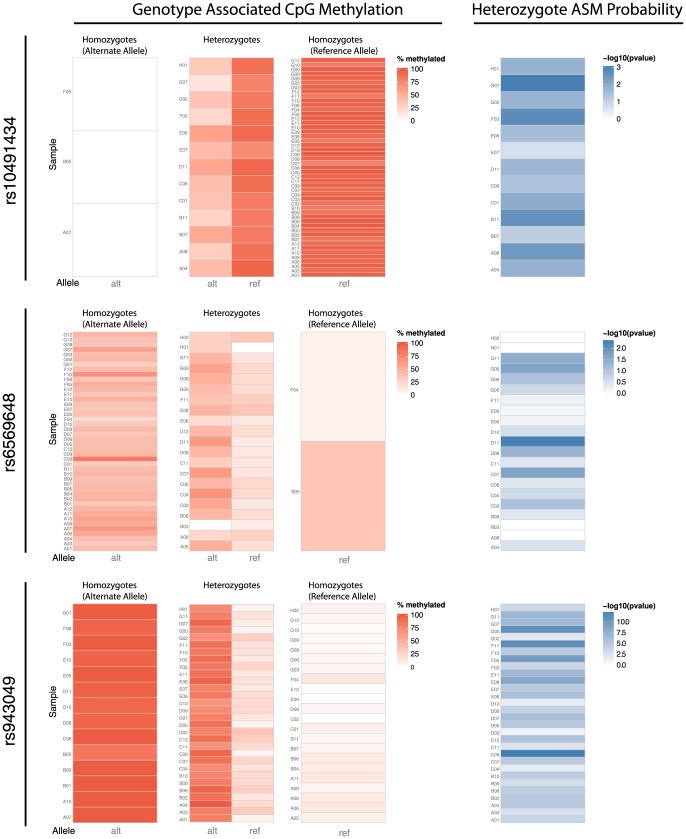
Cis-regulated allele-specific methylation confirmation in an independent population. Heatmaps show percent methylation status for a single CpG within the amplified regions of 3 different MPRs (rs10491434 (top row), rs6569648 (middle row) and rs943049 (bottom row)) for all samples (alternate allele homozygotes (1^st^ column), heterozygotes (2^nd^ column) and reference allele homozygotes. (3^rd^ column)); darker red denotes higher methylation percentages within a sample at the CpG. The final column shows the –log10 p-values derived from chi-square tests for association of methylation with one allele; darker blue results show greater evidence of cis-regulated ASM at the CpG in a particular sample. (CpGs illustrated within this figure are marked with an asterisk in Figure 4).

**Figure 4:**
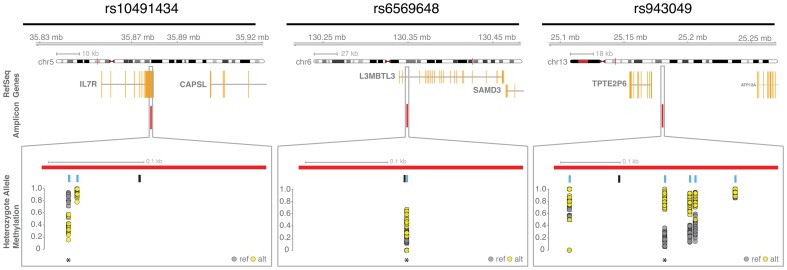
Genomic context of cis-regulated allele-specific methylation events. Illustrations showing genomic context and individual CpG methylation levels at three separate MPRs (rs10491434 (left), rs6569648 (middle) and rs943049 (right)). The chromosomal location of each amplicon is demonstrated with an ideogram. and the RefSeq genes (orange) surrounding the amplicon (red line) shown below. A section (grey box) contracts to the amplicon region itself to show the relative positions of the SNPs (black lines) and CpGs (blue lines) within the amplicons themselves (red bars); methylation levels of the alternate (grey circles) and reference (yellow circles) alleles within samples heterozygous for the SNP are graphed below each CpG. Asterixes (*) mark the CpGs illustrated within Figure 3.

Our results confirm both the presence of cis-regulated ASM at the two positive control variants (rs943049 and rs9366927), and its absence at the negative control variant (rs3738154), none of which are associated with complex disease. More significantly, our results show that three of the seven phenotypic associated variants predicted to exhibit ASM (rs2762051, rs6569648, rs713875), also show cis-regulated ASM in our bisulfite sequencing results, with at least one MSRE associated CpG within each variant's amplicon having a Bonferroni adjusted p-value of less than 0.05, as measured by a chi-square test of the association between the allele and CpG's cytosine methylation states (Table 2 and Table S3). Similarly, one other phenotypic associated variant (rs10491434) while not confirming cis-regulated ASM at an MSRE associated CpG, did show cis-regulated ASM at a nearby non-MSRE associated CpG. Notably, for all ASM associated CpGs, the allele associated with methylation was consistent between the microarray and sequencing analyses. Further analysis of methylation patterns within individual samples confirms these results, showing clear patterns of ASM with varying incidence within the sample set (Figure 3, Figure S4, Figure 4, Figure S5 and Figure S6).

**Table 2:** Confirmation of ASM in a subset of candidate cis-regulated ASM variants.

SNPid	CpG Genomic Position	MSRE CpG	Bonferroni Adjusted p-value	Percent Methylated Reference Allele	Percent Methylated Alternate Allele	Methylated Allele	Assays Consistent
rs10491434	chr5:35877849	NO	8.3E-152	93.9	27.4	REF	YES
	chr5:35877849	YES	1.8E+00	95.6	93.9	REF	YES
rs2021716	chr6:90940976	NO	2.1E-02	89.9	87.5	REF	NO
	chr6:90941090	YES	1.5E+00	93.4	90.9	REF	NO
rs2564921	chr3:53125238	NO	1.1E+00	94.8	92.7	REF	YES
	chr3:53125256	NO	4.8E-01	95.4	92.9	REF	YES
	chr3:53125274	YES	1.0E+01	87.5	87.5	ALT	NO
	chr3:53125271	YES	1.0E+01	97.3	97.2	REF	YES
	chr3:53125164	YES	1.1E+00	97.7	99.4	ALT	NO
rs2762051	chr13:50835759	YES	3.4E-36	71.5	39.1	REF	YES
	chr13:50835802	NO	9.1E-286	1.5	85.8	ALT	NO
rs6569648	chr6:130349122	YES	1.8E-19	44.0	20.9	REF	YES
rs713875	chr22:30592463	NO	8.0E-03	4.0	7.4	ALT	YES
	chr22:30592475	NO	5.9E-03	1.8	4.4	ALT	YES
	chr22:30592477	YES	2.9E-02	2.4	4.7	ALT	YES
	chr22:30592487	NO	NA	NA	NA	ALT	YES
	chr22:30592493	NO	2.0E-03	2.0	4.9	ALT	YES
	chr22:30592871	YES	2.8E+00	13.6	5.4	REF	NO
	chr22:30592946	YES	1.0E+01	11.1	11.1	ALT	YES
rs884488	chr8:101898369	YES	1.0E+01	86.6	86.6	ALT	NO
	chr8:101898533	YES	1.0E+01	42.0	41.9	REF	YES
	chr8:101898673	YES	1.0E+01	93.3	93.3	ALT	NO
rs9366927	chr6:37027391	YES	1.4E-19	67.8	86.9	ALT	YES
	chr6:37027403	NO	1.2E-30	77.4	95.7	ALT	YES
	chr6:37027415	NO	9.4E-76	54.3	91.9	ALT	YES
	chr6:37027424	NO	2.6E-91	54.7	94.6	ALT	YES
	chr6:37027426	NO	9.5E-105	42.0	89.6	ALT	YES
	chr6:37027315	YES	2.3E-89	7.3	49.8	ALT	YES
	chr6:37027178	YES	4.8E-79	15.8	67.9	ALT	YES
rs943049	chr13:25180346	YES	2.3E-02	96.6	94.2	REF	YES
	chr13:25180279	YES	9.5E-288	90.6	29.5	REF	YES
	chr13:25180422	NO	0.0E+00	89.4	16.3	REF	YES
	chr13:25180228	YES	0.0E+00	90.1	12.2	REF	YES
	chr13:25180624	NO	6.7E-14	95.7	74.2	REF	YES
rs3738154	chr1:205064133	YES	1.0E+01	98.2	98.2	ALT	NA
	chr1:205064179	NO	4.8E+00	98.4	98.6	ALT	NA
	chr1:205064224	NO	2.6E+00	98.4	98.8	ALT	NA
	chr1:205064024	YES	3.0E+00	98.5	98.8	ALT	NA
	chr1:205064301	NO	8.3E+00	97.2	97.2	ALT	NA

Results from microarray and next-generation bisulfite sequencing ASM assays of ten variant-containing regions. The table shows the CpG position, whether it is found within an MSRE site and the Bonferroni adjusted chi-square based p-values of the association of methylation at this CpG with either the reference of alternate alleles. The allele with the highest number percentage of methylated reads was designated the most frequently methylated allele (REF = reference, ALT = alternate) and compared to that from the microarray data; for all CpGs that were within MSRE sites and showed significant association of methylation with an allele in the sequencing assay the methylated allele matched that of the microarray assay. For more details, see Table S3.

## Discussion

We present here an evaluation of genome-wide cis-regulated ASM and it relation to expression and phenotypic variation. Our initial screen identified candidate cis-regulated ASM variants that are non-coding, functional eQTLS and/or are in high LD with genetic variants associated with complex phenotypes. We extended this initial screen to confirm cis-regulated ASM at three of these complex phenotype-associated variants in an independent population. Our results suggest that cis-regulated ASM may provide a mechanistic explanation for many of the non-coding genetic changes observed in GWA studies.

In total, 254 unique variants from the NHGRI GWAS catalog (Table S2) are in high LD with at least one candidate cis-regulated ASM variant. Many DNA methylation marks show marked tissue specificity and given recent results by Trynka et al. [4] suggesting that the overlap of regulatory chromatin marks with phenotypically associated variation is cell type specific, further tissue specific studies of ASM may reveal links to other variants within the NGHRI GWAS catalog.

We completely validate three of the seven amplicons predicted to exhibit cis-regulated ASM. Comparison of these confirmation results with our intial microarray screen is complicated by the age differences between our microarray population and our follow-up population, with median ages of 83.5 and 24 years of age respectively. Given the well-established relationship between methylation variation and age [18]–[23], with even genetically identical twins showing increased “epigenetic drift” over time [21], [24], [25] a higher age range might be expected to be associated with greater DNA methylation pattern variation [13]. However, higher false positive rates within the initial screen cannot be ruled out; broader interpretation of candidate ASM loci properties should bear this caveat in mind.

Our microarray screen suggested that multiple eQTLs and GWAS-derived variants show cis-regulated ASM and we confirm three of these MSRE-related CpG methylation events in an independent population. The three variants have been shown to affect multiple phenotypes:

1. rs2762051 is a C/T variant located within the long non-coding RNA DLEU1 and has been implicated in Celiac disease [26].
2. rs713875 is a C/G variant located downstream of the HORMAD2 and LIF genes that has been implicated in multiple diseases, including Crohn's disease [27], IgA nephropathy [28] and early-onset inflammatory bowel disease [29]. It is also an eQTL for MTMR3 [16] and a DNAse sensitive quantitative trait loci for the chr22:28922487–28922487 region [30].
3. rs6569648 is a C/T variant located within the second intron of L3MBTL3 (Figure 4), for which it is also an eQTL [16]; this SNP is one of the hundreds associated with variation in height.

A fourth variant, rs10491434 did not directly confirm our MSRE-based microarray results but did show ASM at a nearby (#bp) non-MSRE CpG. The rs10491434 SNP is a C/T variant located within the 3′UTR of the RefSeq IL7R gene (Figure 4), and is in high LD with the rs3194051 variant implicated in ulcerative colitis [31]. More recently, rs10491434 has been implicated in AIDS progression [32]. Interestingly, this methylation phenotype does not appear to originate from the rs10491434 variant. While MSRE sites were screened for modifying SNPs, CpGs were not and closer analysis of the affected CpG reveals the presence of a variant, rs10491435, within the CpG site itself. This CpG modifying variant is in perfect LD with rs10491434 and is likely responsible for the observed cis-regulated ASM. This variant may influence methylation at the nearby MSRE-CpG in the older microarray population; its close proximity is well within the previously ascribed limits to this local methylation influence [9].

Few studies have examined the association of allele-specific methylation changes with phenotypic traits genome-wide. Allele-specific methylation of MCHR1 is associated with BMI [33]. In cancer progression, Kang et al. report an association of p14ARF (CDKN2A) polymorphisms with the likelihood of methylation of this gene within colorectal cancers [34], while Boumber et al. report an indel polymorphism in the PDLIM4 that influences its methylation in leukemia and colon cancer [35]. More recently, case-control analyses by Liu et al [36] uncovered CpGs within the MHC region which show an association between genotype, the variance of methylation and risk for rheumatoid arthritis. Our study has the advantages of the use of a genome-wide, unbiased screen for allele-specific methylation through the use of a platform specifically designed for differential allele detection. While the results of Liu et al. show that assessment of the role of genetics and methylation in disease progression can be approached by direct study of affected individuals, untargeted studies of ASM such as we present here can supplement these approaches to help identify candidate loci for more intensive, targeted, studies. In that respect, while our results suggest links between cis-regulated ASM at rs10491434, rs2762051 and rs713875 and various autoimmune related phenotypes, further targeted studies of individuals with the relevant phenotypes are necessary to fully substantiate them.

Our choice of technology for the bisulfite confirmation assay was strongly influenced by our desire to examine the association of allele and methylation states without *a priori* knowledge of genotype. As our microarray approach can only assay ASM in heterozygotes, we did not want to exclude the possibility that we would only observe cis-regulated ASM in the heterozygote state. This required observation of the allele and CpG methylation states on the same read; we chose 454 pyrosequencing as it produces reads capable of spanning the distances between target CpGs and variants. Unfortunately, we were unable to obtain quality results for a majority of the amplicons we assayed, largely due to insufficient reads mapping to these amplicons. This is likely at least partially due to biased amplification during sequencing, but also to non-optimal mapping due to extensive homopolymer-related gaps in our reads, a known issue with the 454-pyrosequencing technology (expected to be of even greater impact in the reduced base space of bisulfite sequencing). Supporting this, initial attempts to map these reads with the non-indel aware mapper Bowtie were largely unsuccessful.

Our results have further implications for GWAS. We observe incomplete penetrance of cis-regulated ASM in both our initial screen and our bisulfite sequencing results, or put another way, a lack of consistency in the occurrence of cis-regulated ASM within a group heterozygous individuals [13]. This incomplete penetrance raises the possibility that genetic control of ASM may act as a capricious mediator between a genetic variant and its associated phenotypic outcome, introducing phenotypic variation to nominally identical genotypic backgrounds. This would be expected to reduce the observed odds-ratios/effect-sizes of putative disease causing variants in GWA studies. For instance, the rs713875 variant is only weakly associated with Crohn's disease with an odds ratio of ∼1.08 [27]. It is possible that the local methylation state of rs713875 may provide a more consistent predictor of disease. In this respect, investigations integrating both genetic and of cis-regulated ASM variation may help reveal variant in what has been called “the grey zone” of GWA studies [37], comprised of sub-significant GWAS signals that nonetheless play a role in disease.

## Materials and Methods

These analyses were conducted under the auspices of protocols approved by the institutional review board of Partners Healthcare and Massachusetts Eye and Ear Infirmary. All participants signed consent statements and research adhered to the tenets of the Declaration of Helsinki

### Microarray Study Population

The microarray study population for methylation analyses was derived from the National Academy of Sciences-National Research Council World War II Veteran Twin Registry [38]. Details of this study population have been previously described [39]. DNA samples for methylation analyses were drawn from whole blood samples from 42 total samples. Of these samples, 18 were singletons and 24 were drawn from 12 twin pairs. All samples were genotyped once and all Affymetrix SNP 6.0 methylation array based analyses run in duplicate.

### Resequencing Study Population

Peripheral venous blood was obtained from healthy control volunteers enrolled in The Brigham and Women's Hospital PhenoGenetic Project. The PhenoGenetic Project is a living tissue bank that consists of healthy subjects who are re-contactable and can therefore be recalled based on their genotype. 1,741 healthy subjects >18 years old have been recruited from the general population of Boston. They are free of chronic inflammatory, infectious and metabolic diseases. Their median age is 24, and 62.7% of subjects are women.

For this study whole blood samples were derived from 86 phenotypically normal participants of European ancestry. Four of these samples were subsequently discarded, two due to gender mismatch and two due their status as EIGENSTRAT [40] outliers for European ancestry.

### Microarray Study Population Genotyping

Samples were genotyped on the Affymetrix SNP 6.0 (Santa Clara, California) platform at the Broad Institute Center for Genotyping and Analysis (Cambridge, MA). Each twin pair was genotyped and analyzed with PLINK [41] to determine basic quality assurance and quality control metrics.

### Methyl-Sensitive Restriction Enzyme Digest

For the microarray study, 3 µg of genomic NA was digested at 37 degrees Celsius for 16 hrs with an in the methyl-sensitive restriction enzyme (MSRE) cocktail including Aci I (60 units), BsaH I (3.9 units), Hha I (7.5 units), Hpa II (7.5 units), and HpyCH4 IV (30 units) (New England Biolabs), in a 200 µl reaction volume with 1% BSA and 10% NEB buffer #4 (New England Biolabs) and heat inactivated for 20 minutes at 60°C. Samples were ethanol precipitated, washed in 70% ethanol and resuspended in reduced EDTA TE (5 mM Tris, 0.1 mM EDTA) at 50 ng/µl. All samples underwent the regular Affymetrix SNP 6.0 hybridization procedure in duplicate; samples were digested with the Nsp I and StyI restriction enzymes, fragments of 100–1200 bp (containing the polymorphic sites to be assessed) PCR amplified, and the resulting amplicons labeled and hybridized to the array.

### Methyl-Sensitive Restriction Enzyme Digest Validation

Efficacy of the independent methylation sensitive restriction enzymes was determined by examining MSRE action on amplicons with solitary cut sites for a component MSRE in a control mix run alongside the study samples. Our results showed amplicons with single MSRE cut sites, with the exception of HhaI, exhibited greatly reduced combined probe intensities as compared to amplicons with no MSRE sites (or MSRE Negative Regions (MNRs)) (Figure S7). Amplicons with only HhaI sites (6132 in total) were removed from further analyses.

### Methylation Array Quality Control

To eliminate replicates with poor hybridizations, the un-normalized probe intensities of the replicates of MSRE digested samples were compared by linear regression. All replicate coefficients of determination were within 2.5 standard deviations of the mean of the entire sample set (mean = 0.919, standard deviation = 0.125). After normalization, the mean coefficient of determination was 0.94 with a standard deviation of 0.028.

### Methylation Array Normalization and ASM Detection

For further details on methods development, see Supporting Information (Information S1, Figure S8, Figure S9, Figure S10, Figure S11, Figure S12, Figure S13 and Table S4). Briefly, invariant probesets were quantile normalized between arrays and normalized values for variant probesets interpolated from the normalized invariant probesets derived from their respective arrays. After various levels of technical filtering by multiple criteria (Figure S1) allele-specific methylation at heterozygous SNPs was detected as a sample and SNP-specific normalized deviation from the HapMap derived heterozygote relative probe intensity. To account for inter-probeset variation we standard normalized all post-MSRE treatment MPR log2(A/B) values against the HapMap distribution for that MPR to derive a Standard Score; MPRs with different HapMap log2(A/B) distributions then had comparable Standard Score distributions. To account for inter-sample technical variability within this standard score we used the samples' MNR Standard Score distributions (which are not expected to vary between samples) to determine the final ASM calls; MPRs with values lower than the 2.5 and 97.5 percentiles of the MNR distribution for a sample were called ASM (Figure 1). All microarray data are available upon request from Dr. Seddon.

### GWAS and EQTL Enrichment Analyses

Independent GWAS variants from the NHGRI GWAS Catalog [17] were restricted to annotated results with annotated p-values of less than 1×10^−7^ and at least 500 individuals in the “Total Initial Sample Size”. All sets of SNPs were pruned to produce subsets with pair wise linkage-disequilibrium (LD) values of no more than 0.3. Minor allele frequencies (MAFs) for MAF matching between sets of SNPs were calculated from the 1000 genomes project.

For eQTL derivation, associations between SNP genotypes and adjusted expression values were conducted using Spearman Rank Correlation (SRC). For the cis-eQTL analysis, we considered only SNPs within a 1 MB window from the transcript start site (TSS) of genes. Significance of the nominal p-values was determined by comparing the distribution of the most significant p-values generated by permuting expression phenotypes 10,000 times independently for each gene. Significant *cis*-eQTLs were those with a nominal association P-value greater than the 0.05 tail of the minimal P-value distribution resulting from the SNP's associations with 10,000 permuted sets of expression values or each gene.

Enrichment of cis-regulated ASM in eQTLs was calculated by chi-squared test. Random LD pruned SNPs from sets of both non-ASM SNPs and cis-regulated ASM SNPs (10 sets each) were examined to determine the proportion that were also found within sets of eQTL SNPs derived from either monocytes or peripheral blood mononuclear cells (PBMCs).

### Resequencing Primer Design

As most of the candidate target amplicons were larger than the maximum read length (minimum length of 221 bp and a median length of 362.5 bp) typically obtained with Illumina technologies, we employed 454 large-scale parallel pyrosequencing in order to examine SNP and CpG site methylation status on the same read. Primers for bisulfite PCR (BSP) amplification were designed with Methprimer [42] and BiSearch [43] to uniquely amplify fragments of less than 500 bp encompassing the MSRE sites and target SNP, when possible. For some variants, two amplicons were necessary to cover all predicted MSRE sites and the target SNP, for others, it was necessary to use proxy SNPs to report the status of target SNPs too far away from MSRE sites assayed by the microarray assay. All amplifications were strand specific and were designed to allow observation of the SNP status after bisulfite conversion and amplification; for C/T SNPs, the guanine strand was amplified. Primer sequences (against bisulfite converted DNA) and amplicon genomic locations for all amplicons can be found in Table S5.

### Resequencing Bisulfite PCRs

The set of DNA samples from 82 phenotypically normal individuals were bisulfite converted, and the initial set of the 32 target regions amplified. DNA was bisulfite converted with the Qiagen EpiTect 96 Bisulfite Kit according to the standard protocol in the manufacturer's instructions by centrifugation without carrier tRNA. Converted DNA was quantitated by Nanodrop and separate aliquots amplified in 96 well plates for each amplicon (Figure S14). Each 20 ul PCR reaction comprised 10 ng of converted DNA, 1 unit of Qiagen HotStarTaq Plus, 50 picomoles of each primer (2.5 µM final concentration), 1× PCR buffer, and 200 nanomoles of dNTPs (10 mM final concentration) and was run for 35 cycles. Melting temperatures and extension times were empirically tested for each primer set to a) optimize product yield while b) reducing amplification of unconverted DNA and off-target products. Final individual amplification conditions can be found in the Supplementary Information (Table S5). A random subset of amplification products from each plate was visually confirmed by agarose gel electrophoresis and all amplification products quantitated by Picogreen. Equimolar amounts from each target's amplification products were mixed for each individual and prepared for bar-coded 454 Pyrosequencing.

### Resequencing Read Alignment

Pooled reads were separated by individual according to bar-coded index. Sff formatted reads were converted to fastq format with sff_extract version 0.2.13 and adapter and low quality sequences clipped; reads shorter than 100 were removed using fastx_clipper. Reads were non-directionally aligned to unconverted amplicon sequences with Bismark [44]. To adjust for small insertions/deletions which result from 454 sequencing's known homopolymer issues [45], we ran Bismark with the Bowtie 2 gapped aligner with a multi-seed length of fifteen with one mismatch. Up to 20 consecutive seed extension attempts were attempted before accepting a read alignment. After barcode filtering, reads mapped to the amplicon reference sequence by Bismark with Bowtie 2 aligner at a mean of 49.6%.

Bisulfite conversion rates on all reads were calculated with the Bismark methylation_extractor script (Figure S15). Individual reads were not filtered by conversion rate to avoid biases against CpG methylated reads which might result from removal of reads containing correlated methylated non-CpGs (i.e. CHG and CHH). Instead we looked for samples with failed bisulfite conversions, i.e. samples with a mean conversion rate lower than 95%. Examination of bisulfite conversion at non-CpG cytosines showed successful conversion in all samples, with an average conversion rate of 98.7%. In order to obtain reads with information for both the SNP and MSRE site status, reads which did extend over both the amplicon's target SNP and MSRE sites were then discarded.

### SNP Genotyping and Quality Control

Genotypes at target SNPs were called in VCF format with samtools mpileup [46] with extended BAQ, and a BAQ cutoff of 10; indels were not called. To ensure quality analyses, we filtered our samples and amplicons based on coverage depth, genotype quality, Hardy-Weinberg equilibrium with the sample population, and agreement expected minor allele frequency. Samples or amplicons with consistently low confidence genotypes (depth of coverage less than 20, genotype quality less than 20) at the target SNP in a majority (>50%) of samples or amplicons were discarded (Figure S16). Hardy-Weinberg and minor allele frequencies for all amplicon target SNPs were calculated for the population with VCFtools [47]. Amplicons with SNPs deviating from the expected Hardy-Weinberg equilibrium (p<0.01) and any amplicons whose minor allele frequency showed a relative mean difference of more than 0.25 from the expected CEU minor allele frequency (HapMap release 27) were discarded. Filtering amplicons or samples with low read coverage or called genotype quality in a majority of our set of samples or amplicons respectively removed 21 of the 32 amplicons and 12 of the 82 samples leaving 11 amplicons and 70 samples. Further filtering based on deviation from the expected Hardy-Weinberg equilibrium (p<0.01), removed 1 amplicon. All remaining amplicons also had minor allele frequencies with relative mean differences of less than 0.2 from the expected CEU minor allele frequency (HapMap release 27) (Figure S17).

## Supporting Information

Figure S1 **Technical filtering.** MPRs were filtered for quality and potential technical artifacts by multiple criteria. Of the ∼910,000 amplicons on the Affymetrix SNP6.0 array, ∼∼150,000 had no predicted MSRE sites, and were used to normalize between arrays but discarded from downstream analyses. We also removed amplicons with MSRE sites that did not perform well on our HapMap reference set; ∼70,000 amplicons had no calls across the entire HapMap samples, and ∼180,000 had poor separation (or low “discrimination”) between the 3 log2 (A/B) distributions for the 3 genotype classes (AA, AB and BB). The potential for artifacts arising from polymorphisms in MSRE sites was eliminated in a similar manner to that previously described [9]. Briefly, we excluded all SNPs on the Affymetrix array residing on amplicons containing any polymorphism in an MSRE site with a minor allele frequency more than 4% in individuals of European descent from the 1000 Genomes Project data [48]. Although this filter may discard SNPs that do not reside on amplicons with MSRE site polymorphisms in the individuals examined here, we conservatively chose to ensure robust analyses by eliminating any SNP expected to appear at least once in the microarray study population.
(PDF)

Figure S2 **Detection of previously identified allele-specific methylation events.** Shown are the standard scores (or Standard Scores) of heterozygote samples after probeset normalization against the log2 (A/B) distribution of heterozygote undigested HapMap samples for four MPRs found in genomic regions known to be associated with allele-specific methylation; rs220030 is a SNP within the imprinted *SNRPN* locus (A); rs2107425 is a SNP located ∼2 kb upstream of the imprinted *H19* locus (B); rs6494120 is an intergenic SNP located ∼11 kb upstream of *GCNT3* (C) and rs943049 is an intergenic SNP located ∼75 kb upstream of *ATP12A* (D). Red and blue circles denote MSRE treated and untreated samples respectively. Open and closed circles denote samples for which an allele-specific methylation event was and was not observed, respectively. Standard scores with a negative value denote allele-specific methylation of the B allele (i.e. log2 (A/B)<0) and those with a positive value denote allele-specific methylation of the A allele (i.e. log2 (A/B)>0) (base identities of the A and B alleles are indicated for each variant). For MPRs within known imprinted regions (panels A and B), an approximately equal number of allele-specific methylation events at the A and B alleles is observed, consistent with a pattern of allele-specific methylation based on allelic parent-of-origin within our sample population. The differential methylation patterns of MPRs found in genomic regions previously associated with cis-regulated allele-specific methylation (panels C and D) are consistent with previous results, i.e. only one allele is associated with methylation.
(PDF)

Figure S3 **Genomic properties of cis-regulated allele-specific methylation candidates.** Non-genic localization (upstream or downstream of annotated gene, blue) versus genic localization (5′UTR, 3′UTR, exons or introns of an annotated gene, red) of candidate cis-regulated ASM regions (A). Non-genic (upstream or downstream of annotated gene) candidate cis-regulated ASM regions are located a median distance of 129 kb from the closest gene (B).
(PDF)

Figure S4 **Assessing allele-specific methylation in an independent population.** Results for all individual CpGs of the ten amplicons (A - rs10491434_BSP; B - rs2021716_BSP; C - rs2564921_BSP; D - rs2762051_BSP; E - rs3738154_BSP; F - rs6569648_BSP; G - rs713875_BSP; H - rs884488_BSP; I - rs943049A_BSP; J - rs9366927_BSP) are shown. Heatmaps show percent methylation status for single CpGs within the amplified regions of MPRs for all samples (alternate allele homozygotes (1^st^ column), heterozygotes (2^nd^ column) and reference allele homozygotes. (3^rd^ column)); darker red denotes higher methylation percentages within a sample at the CpG. The final column shows the –log10 p-values derived from chi-square tests for association of methylation with one allele; darker blue results show greater evidence of cis-regulated ASM at the CpG in a particular sample.
(PDF)

Figure S5 **Genomic context of amplicons and methylation events.** Illustrations showing genomic context and individual CpG methylation levels for ten amplicons (A - rs10491434_BSP; B - rs2021716_BSP; C - rs2564921_BSP; D - rs2762051_BSP; E - rs3738154_BSP; F - rs6569648_BSP; G - rs713875_BSP; H - rs884488_BSP; I - rs943049A_BSP; J - rs9366927_BSP) are shown. The chromosomal location of each amplicon is demonstrated with an ideogram and the RefSeq genes (orange) surrounding the amplicon (red line) are shown below. A section (grey box) contracts to the amplicon region itself to show the relative positions of the SNPs (black lines) and CpGs (blue lines) within the amplicons themselves (red bars); methylation levels of the alternate (grey circles) and reference (yellow circles) alleles within samples heterozygous for the SNP are graphed below each. The final bottom plot shows, for individual heterozygote samples, the results of chi-square tests of the association between allele state and individual CpG's cytosine methylation states, reported as–log10 transformed p-values.
(PDF)

Figure S6 **Methylation patterns of amplicons.** Illustrations showing methylation patterns for ten amplicons at both a population level and within a representative individual. “Lollipop” plots show both methylation calls for 30 randomly drawn reads from each allele from the entire population (left plot) and for a representative heterozygous individual for that amplicon (right plot). Representative individuals were selected by finding the individual closest to the median chi-square based p-value for the CpG with the highest likelihood of ASM based on lowest mean chi-square based p-value. CpG methylation status is shown in black (methylated) and white (unmethylated) while SNP status is shown in blue (reference allele) and orange (alternate allele). CpGs or SNPs on a read without information (typically due to gaps in the read) are displayed in grey.
(PDF)

Figure S7 **Individual assessment of methylation-sensitive restriction enzyme digest efficacy.** Density plots are shown for assayed probe intensities for amplicons with single MSRE sites for each of the five MSRE enzymes used as well as for MNRs (UNCUT). Intensities are expressed as the assayed total intensities for these amplicons normalized against the total intensities for these amplicons in the HapMap samples. All MSREs with the exception of HhaI exhibited reduced overall intensities as compared to amplicons without MSRE sites (MNRs/UNCUT).
(PDF)

Figure S8 **Derivation of intensity ratio cutoff.** To filter out potential false positives derived from biallelic unmethylated MPRs a filter based on the intensity ratio of the MSRE treated MPRs as compared to that of the HapMap reference samples. The threshold was chosen to screen out biallelic unmethylated MPRS while still passing any ASM MPRs (which would be expected to show reduced overall intensities as compared to biallelic methylated MPRs). The final value of this filter was based on observation of this intensity ratio in 1∶1 unmethylated control mixes, which are expected to model the properties of monoallelic methylated MPRs at all assayed amplicons. At an intensity ratio value of 0.2, only 0.2% of these mock ASM MPRs were filtered out.
(PDF)

Figure S9 **Assessment of traditional Affymetrix SNP6.0 array normalization methods.** Scatter plots (left panels) and histograms (right panels) of un-normalized (top panels), median normalized (middle panels) and quantile normalized (bottom panels) probe intensities for both MSRE undigested and MSRE digested samples of an unmethylated control are shown.
(PDF)

Figure S10 **Method of MNR selection.** Amplicons were chosen as MNRs based on two criteria: 1) assay based size selection of amplicons results in a final amplicon size range of 200–1200 bp and 2) bioinformatic prediction of MSRE site locations. These criteria allow selection of 3 classes of MNR amplicons expected to show no effect of MSRE digestion: a) those with both NspI and StyI amplicons of 200–1200bp with no MSRE sites (1st alternative) and those with either b) NspI amplicons of 200–1200 bp with no MSRE sites and StyI amplicons outside this size range (2nd alternative, top) or c) StyI amplicons of 200–1200 bp with no MSRE sites and NspI amplicons outside this size range (2nd alternative, bottom).
(PDF)

Figure S11 **MPRs show lower combined probe intensities relative to MNRs after MSRE digest.** Scatter plot (top panel) and density plots (bottom panels) of total probe intensities before (top panel and bottom left panel) and after MSRE digestion (top panel and bottom right panel) for amplicons with (MPRs - in red) and without (MNRs - in blue) MSRE sites in an unmethylated control sample. MPRs show a pronounced shift to lower intensities after MSRE digestion.
(PDF)

Figure S12 **Allele frequencies vary with MSRE digest for MPRs but not MNRs in control methylation mixes.** Scatter plots of A allele frequencies (probe A intensity/(probe A intensity + probe B intensity) for amplicons with (MPRs - in red) and without (MNRs - in blue) MSRE sites from two 50∶50 E44-H16 control mix samples, one where one sample has H16 methylated and E44 unmethylated (y-axis) and the other H16 unmethylated and E44 methylated (x-axis). In the top panel, MPRs where E44 contributes the A allele are shown and in the bottom panel, E44 contributes the B allele. MSRE digest does not change the distribution for MNRs but shifts those of MPRs towards the axis of the mix with the methylated A allele.
(PDF)

Figure S13 **Assessment of MNRs based quantile interpolation normalization method in control methylation mixes.** Scatterplots are shown of the probe A intensity values of un-normalized (left panels) and normalized (right panels) of two replicates from two separate reciprocal 50∶50 E44:H16 control mixes. In these 50∶50 mixes, only one of the samples is methylated; in the top panels, the E44 sample is methylated and the H16 unmethylated, in the bottom panels the H16 sample is methylated and the E44 sample unmethylated. The MNR quantile interpolated normalization based method used greatly reduced variation between replicates.
(PDF)

Figure S14 **Bisulfite PCR methods.** A simplified representation of the bisulfite PCR sequencing assays. Sample DNA was plated in 96 well plates, bisulfite converted and aliquoted into separate 96 well plates for each amplification target (left panel). Each 96 well plate was subjected to bisulfite-specific PCR (BSP), amplifications were combined in equimolar amounts by individual into a single 96 well and bar-coded before sequencing. Primers were designed to flank both the target SNP (here a G/A SNP on the top strand, middle and right panels) and CpG, amplify only bisulfite converted DNA and be strand specific in a manner that allowed allele identification after bisulfite conversion. Example amplifications for a scenario where the G-allele is not associated with CpG methylation (middle panel) and the A allele is associated with a methylated CpG (right panel) are shown. Note that strand specific amplification of the C/T SNP on the bottom strand would not allow allele identification after bisulfite conversion.
(PDF)

Figure S15 **Bisulfite conversion rates.** Distributions of median bisulfite conversion rates for all non-CpG cytosines in each amplicon are shown for each sample. Both histograms (grey) and density plots (orange) are shown.
(PDF)

Figure S16 **Amplicon and sample genotype coverage and qualities.** Target SNP read coverage (A and C) and genotype quality (B and D) distributions across all samples for each amplicon (A and B) or across all amplicons for each sample (C and D) are shown. Amplicons failed this test (orange filled distributions) if the majority of samples had either less than 20-fold coverage or genotype qualities below 20. Amplicons that passed these quality filters (grey filled distributions in both panels A and B) were carried forward. Similarly, samples failed these tests if the majority of amplicons had either less than 20-fold coverage or genotype qualities below 20.
(PDF)

Figure S17 **Hardy Weinberg Equilibrium and allele frequency based amplicon filtering.** Reference allele frequencies and heterozygote frequencies for assayed variants. A) Observed versus expected (HapMap) reference allele frequencies are shown. Colors of plotted data reflect the relative mean difference of the observed minor allele frequency from the expected CEU minor allele frequency (HapMap release 27). B) Observed versus expected heterozygote frequencies as based on observed allele frequencies. Colors of plotted data reflect the p-values from of an exact test as defined by [49].
(PDF)

Table S1 **MPR ASM levels are significantly higher than expected from MNR allele-specific methylation levels.** Shown are numbers of heterozygous MPRs assessed for ASM within each sample, the expected number of ASM MPRs based on the MNR Standard Score distribution (i.e. 95% non-ASM, or 5% ASM), the actual observed number, fold increase over expected and the associated chi-square based p-value.
(XLS)

Table S2 **NHRI GWAS SNPs in high LD with candidate cis-regulated ASM SNPs.** Details are shown for both NHGRI variants in high linkage disequilibrium with cis-regulated ASM variants and for genes regulated by eQTLs that are also cis-regulated ASM variants. Study details for the NHGRI variants and the allelic association counts observed in the microarray assay are also shown.
(XLS)

Table S3 **Detailed confirmation of ASM by sequencing in a subset of candidate cis-regulated ASM variants.** Results from microarray and next-generation bisulfite sequencing ASM assays of ten variant-containing regions. For each amplicon region the table shows the variant and its categorization. Autoimmune variants that exhibited ASM in the microarray study are labeled “AI ASM”, similar non-autoimmune NHGRI variants are labeled “NHGRI ASM”. “Known ASM” variants have been previously shown to exhibit ASM. “Non ASM” variants did not exhibit ASM in the microarray study. For the bisulfite next generation sequencing analyses, the table shows the CpG position within the amplicon and whether it is found within an MSRE site; the reference and alternate allele sequences after bisulfite conversion; the number of methylated and unmethylated CpGs associated with the reference and alternate alleles and the unadjusted and Bonferroni adjusted Chi-square p-values of those associations. The allele with the highest number percentage of methylated reads was designated the most frequently methylated allele (REF = reference, ALT = alternate). For the microarray data, the number of heterozygotes that were observed with ASM associated with either the “A” or “B” alleles were used to determine the identity of the most frequently methylated allele which was matched against the most frequently methylated allele in the sequencing data. within the microarray and next-generation studies. For all CpGs that were within MSRE sites and showed significant association of methylation with an allele in the sequencing assay, the methylated allele matched that of the microarray assay.
(XLSX)

Table S4 **MNR properties.** Details about the genomic location and MSRE sites present on MNR NspI and StyI based amplicons are shown. For each of the NspI and StyI amplicons, the genomic location and amplicon size are presented. The number of each of the 5 potential MSRE types (AciI, BsaHI, HhaI, HpaII and HpyCH4IV) within the amplicon are shown before the bracket. Within the bracket, the first number denotes the number of dbSNP-derived variants that could add an MSRE site while the second number denotes the number, which could remove an MSRE site.
(XLSX)

Table S5 **PCR properties.** Properties of regions assayed by bisulfite next-generation sequencing. For each amplicon, this table reports its intended purpose of the amplicon, whether it be as controls like CpG islands and X-chromosomal, imprinted and known ASM regions, or as confirmation of the microarray results, like regions without ASM (Non ASM), regions with random ASM or regions with linked variants associated with either NGHRI or autoimmune phenotypes. The table also reports the identity of the variant within the amplicon (“reporting variant”), the linked NHGRI/autoimmune variant, the sequences of the bisulfite PCR primers, the melting temperature used for the bisulfite PCR, the predicted product length and the genomic region amplified by the bisulfite PCR.
(XLSX)

Information S1 **Supplementary methods and references.**
(DOC)
