# Supplementary figures and images for: Allele-Specific Methylation Occurs at Genetic Variants Associated with Complex Disease
Source: PLoS One. 2014 Jun 9;9(6):e98464. doi: 10.1371/journal.pone.0098464 (PMC4049588; doi:10.1371/journal.pone.0098464)

Figure S1

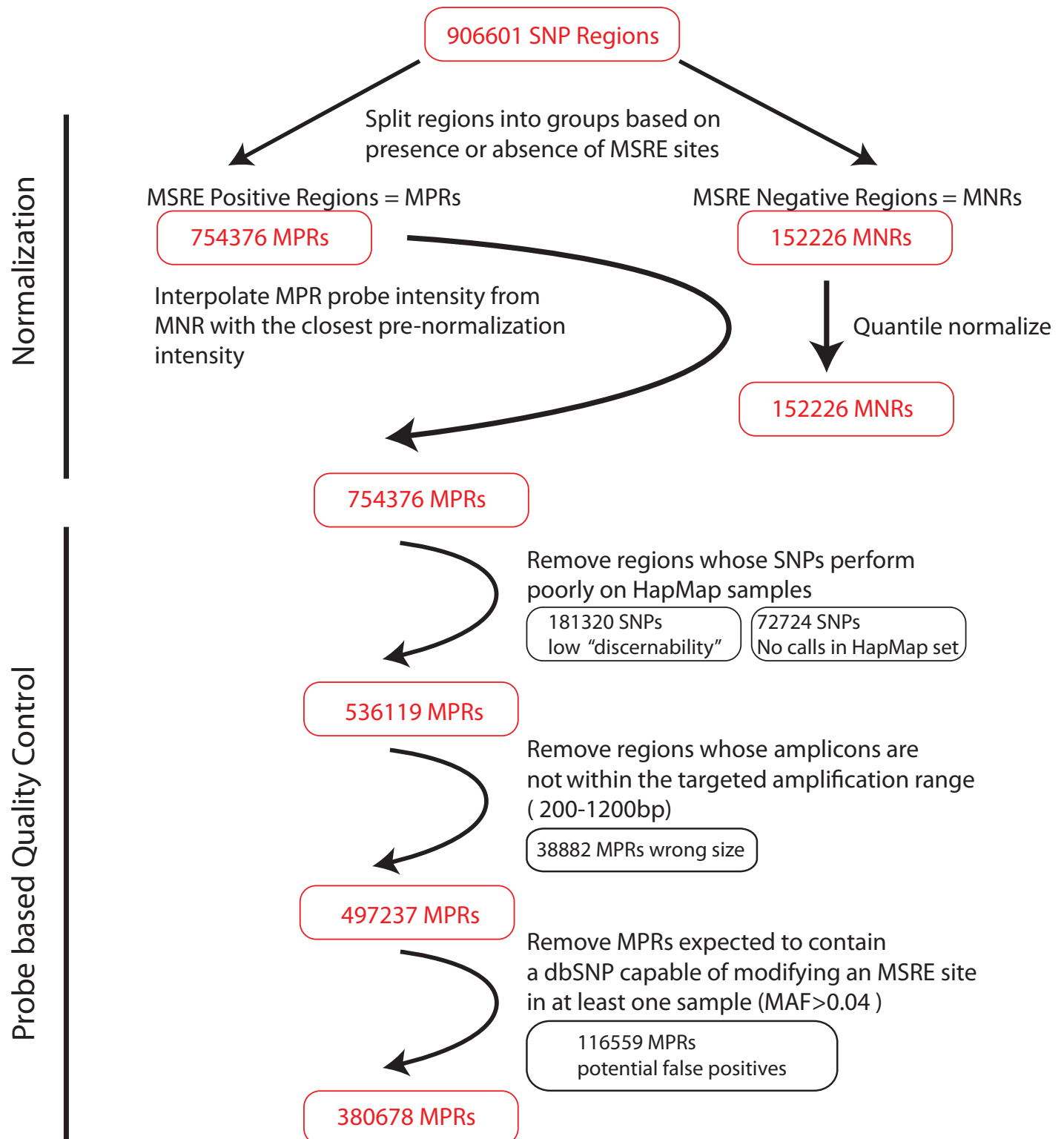

Supplement: Figure S1 — Technical filtering. MPRs were filtered for quality and potential technical artifacts by multiple criteria. Of the ∼910,000 amplicons on the Affymetrix SNP6.0 array, ∼∼150,000 had no predicted MSRE sites, and were used to normalize between arrays but discarded from downstream analyses. We also removed amplicons with MSRE sites that did not perform well on our HapMap reference set; ∼70,000 amplicons had no calls across the entire HapMap samples, and ∼180,000 had poor separation (or low “discrimination”) between the 3 log2 (A/B) distributions for the 3 genotype classes (AA, AB and BB). The potential for artifacts arising from polymorphisms in MSRE sites was eliminated in a similar manner to that previously described [9]. Briefly, we excluded all SNPs on the Affymetrix array residing on amplicons containing any polymorphism in an MSRE site with a minor allele frequency more than 4% in individuals of European descent from the 1000 Genomes Project data [48]. Although this filter may discard SNPs that do not reside on amplicons with MSRE site polymorphisms in the individuals examined here, we conservatively chose to ensure robust analyses by eliminating any SNP expected to appear at least once in the microarray study population. (PDF) [file pone.0098464.s001.pdf]

Figure S2

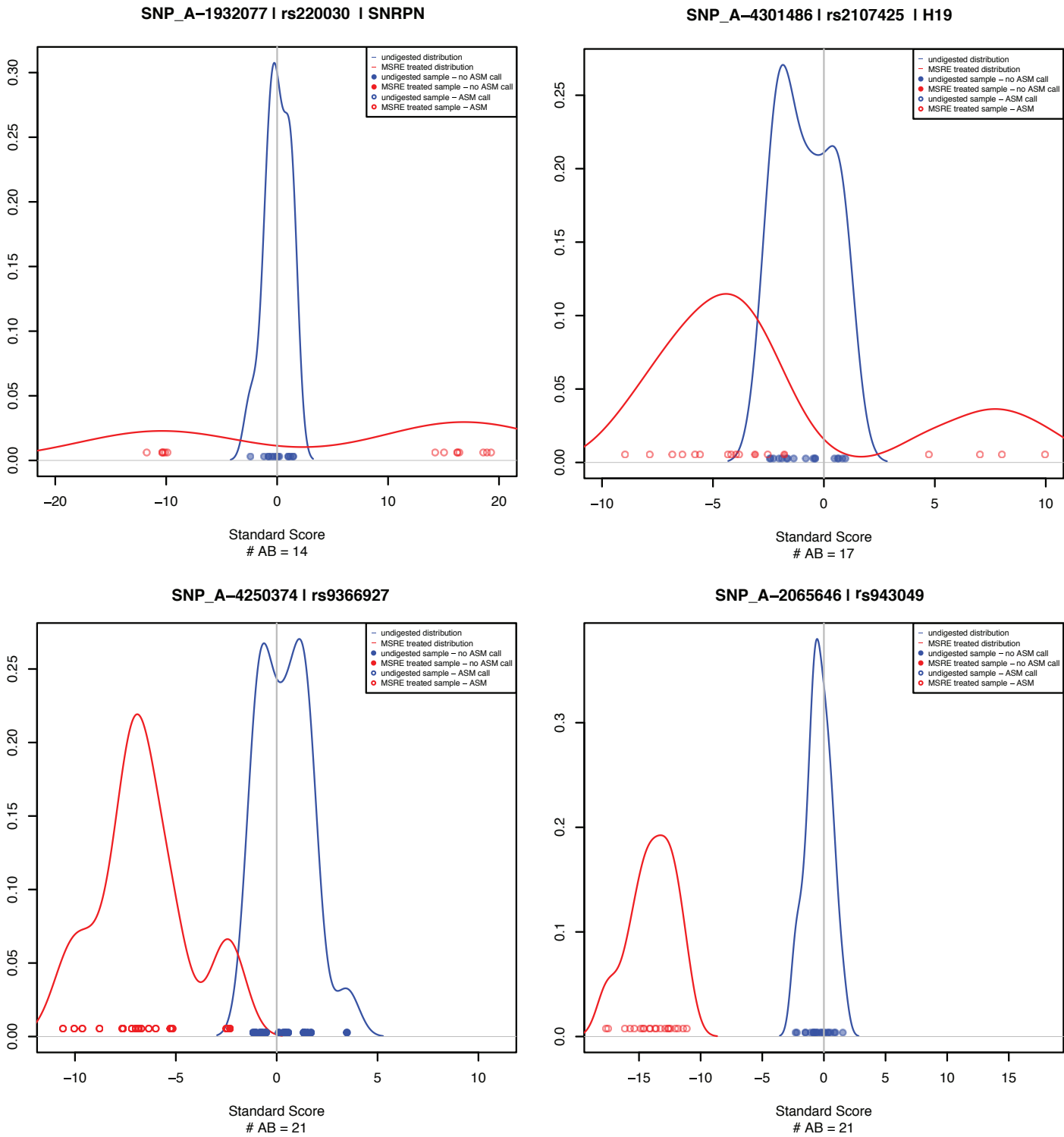

Supplement: Figure S2 — Detection of previously identified allele-specific methylation events. Shown are the standard scores (or Standard Scores) of heterozygote samples after probeset normalization against the log2 (A/B) distribution of heterozygote undigested HapMap samples for four MPRs found in genomic regions known to be associated with allele-specific methylation; rs220030 is a SNP within the imprinted SNRPN locus (A); rs2107425 is a SNP located ∼2 kb upstream of the imprinted H19 locus (B); rs6494120 is an intergenic SNP located ∼11 kb upstream of GCNT3 (C) and rs943049 is an intergenic SNP located ∼75 kb upstream of ATP12A (D). Red and blue circles denote MSRE treated and untreated samples respectively. Open and closed circles denote samples for which an allele-specific methylation event was and was not observed, respectively. Standard scores with a negative value denote allele-specific methylation of the B allele (i.e. log2 (A/B)<0) and those with a positive value denote allele-specific methylation of the A allele (i.e. log2 (A/B)>0) (base identities of the A and B alleles are indicated for each variant). For MPRs within known imprinted regions (panels A and B), an approximately equal number of allele-specific methylation events at the A and B alleles is observed, consistent with a pattern of allele-specific methylation based on allelic parent-of-origin within our sample population. The differential methylation patterns of MPRs found in genomic regions previously associated with cis-regulated allele-specific methylation (panels C and D) are consistent with previous results, i.e. only one allele is associated with methylation. (PDF) [file pone.0098464.s002.pdf]

Figure S3

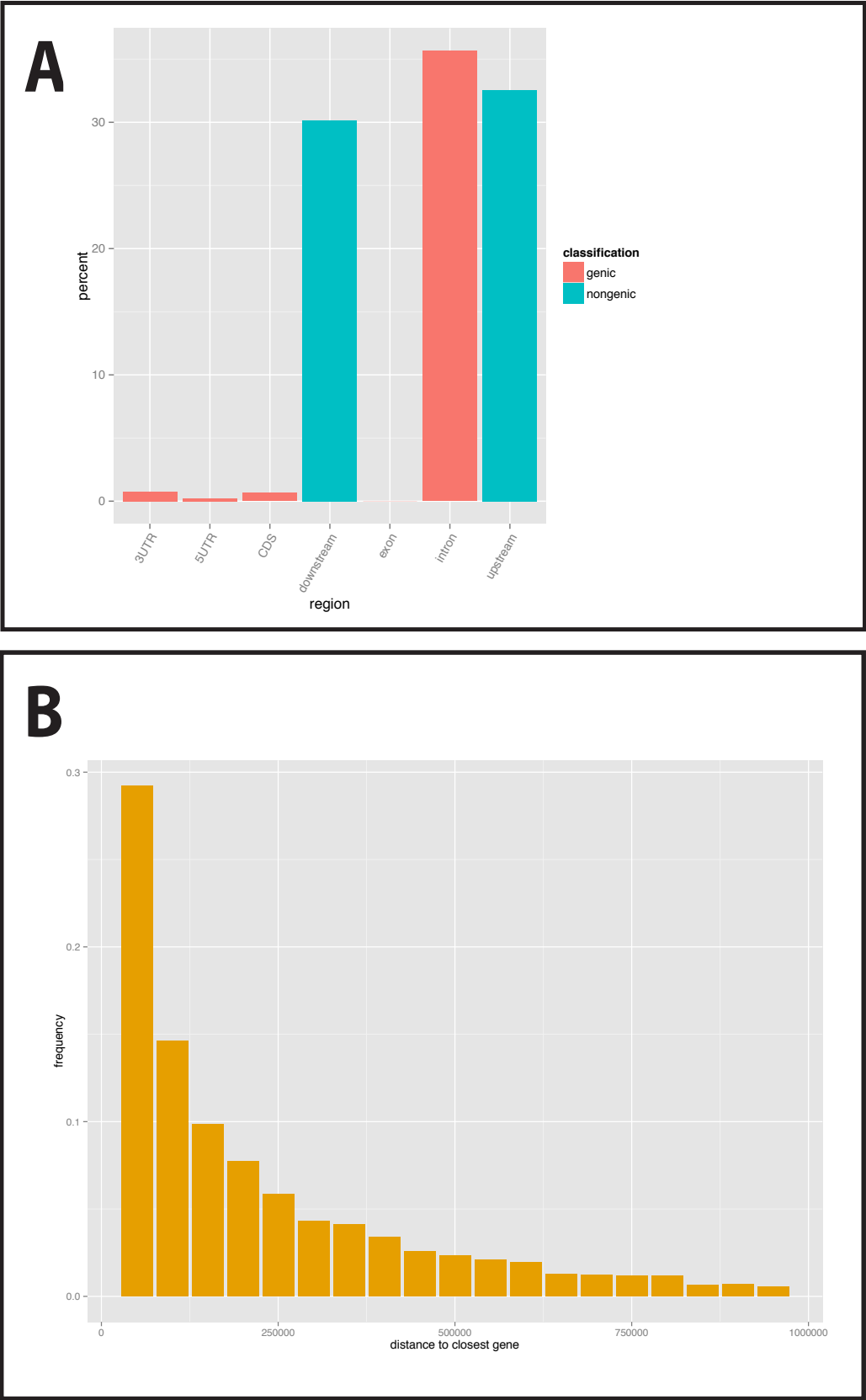

Supplement: Figure S3 — Genomic properties of cis-regulated allele-specific methylation candidates. Non-genic localization (upstream or downstream of annotated gene, blue) versus genic localization (5′UTR, 3′UTR, exons or introns of an annotated gene, red) of candidate cis-regulated ASM regions (A). Non-genic (upstream or downstream of annotated gene) candidate cis-regulated ASM regions are located a median distance of 129 kb from the closest gene (B). (PDF) [file pone.0098464.s003.pdf]

Figure S7

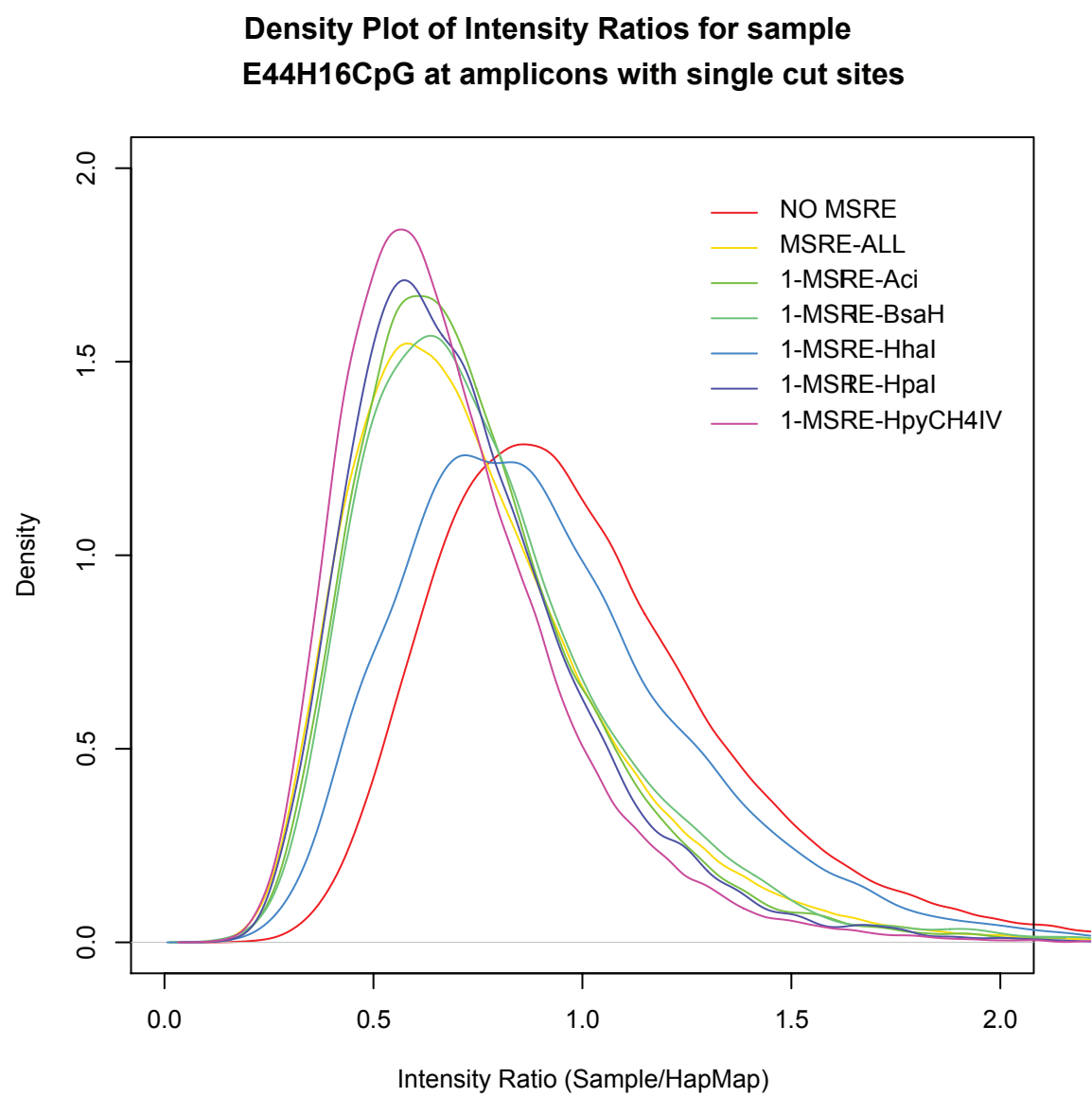

Supplement: Figure S7 — Individual assessment of methylation-sensitive restriction enzyme digest efficacy. Density plots are shown for assayed probe intensities for amplicons with single MSRE sites for each of the five MSRE enzymes used as well as for MNRs (UNCUT). Intensities are expressed as the assayed total intensities for these amplicons normalized against the total intensities for these amplicons in the HapMap samples. All MSREs with the exception of HhaI exhibited reduced overall intensities as compared to amplicons without MSRE sites (MNRs/UNCUT). (PDF) [file pone.0098464.s007.pdf]

Figure S8

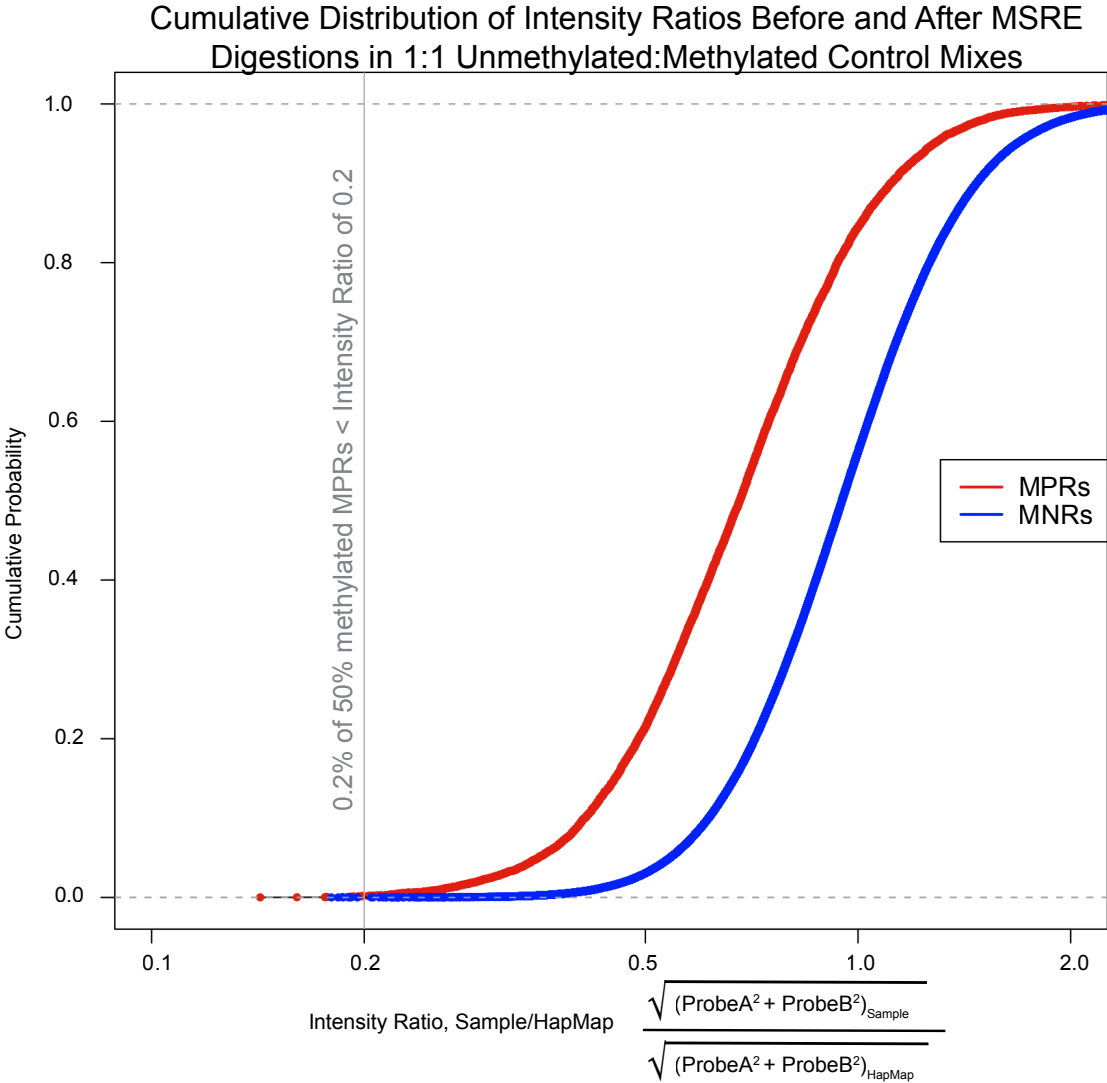

Supplement: Figure S8 — Derivation of intensity ratio cutoff. To filter out potential false positives derived from biallelic unmethylated MPRs a filter based on the intensity ratio of the MSRE treated MPRs as compared to that of the HapMap reference samples. The threshold was chosen to screen out biallelic unmethylated MPRS while still passing any ASM MPRs (which would be expected to show reduced overall intensities as compared to biallelic methylated MPRs). The final value of this filter was based on observation of this intensity ratio in 1∶1 unmethylated control mixes, which are expected to model the properties of monoallelic methylated MPRs at all assayed amplicons. At an intensity ratio value of 0.2, only 0.2% of these mock ASM MPRs were filtered out. (PDF) [file pone.0098464.s008.pdf]

Figure S9

A

Unnormalized

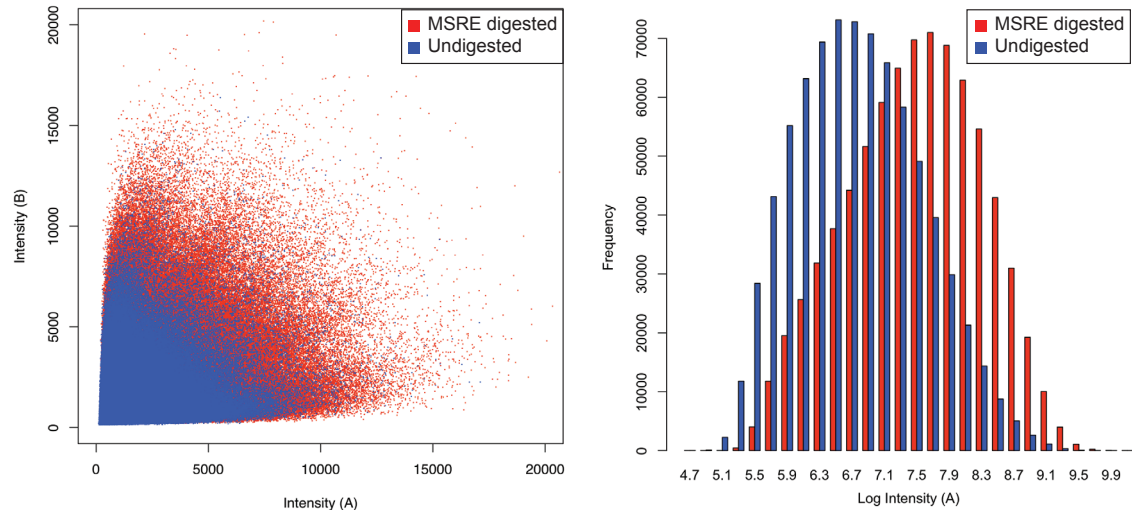

B

Median Normalized

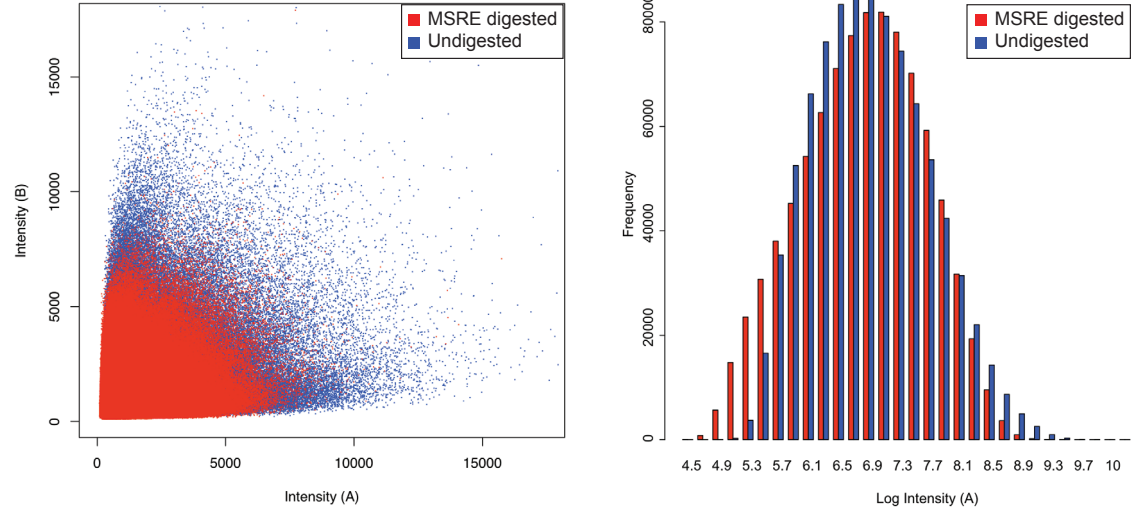

C

Quantile Normalized

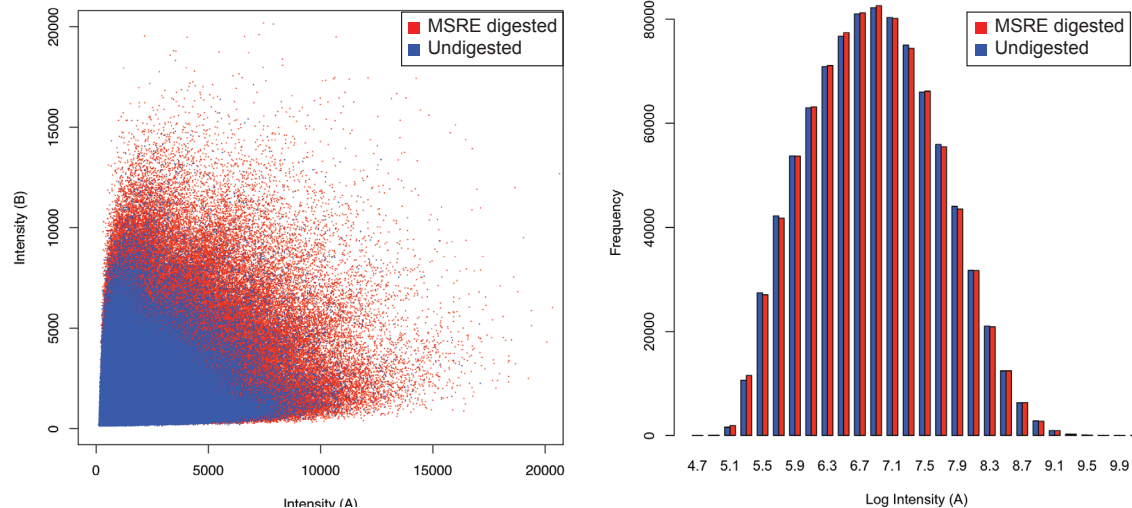

Supplement: Figure S9 — Assessment of traditional Affymetrix SNP6.0 array normalization methods. Scatter plots (left panels) and histograms (right panels) of un-normalized (top panels), median normalized (middle panels) and quantile normalized (bottom panels) probe intensities for both MSRE undigested and MSRE digested samples of an unmethylated control are shown. (PDF) [file pone.0098464.s009.pdf]

Figure S11

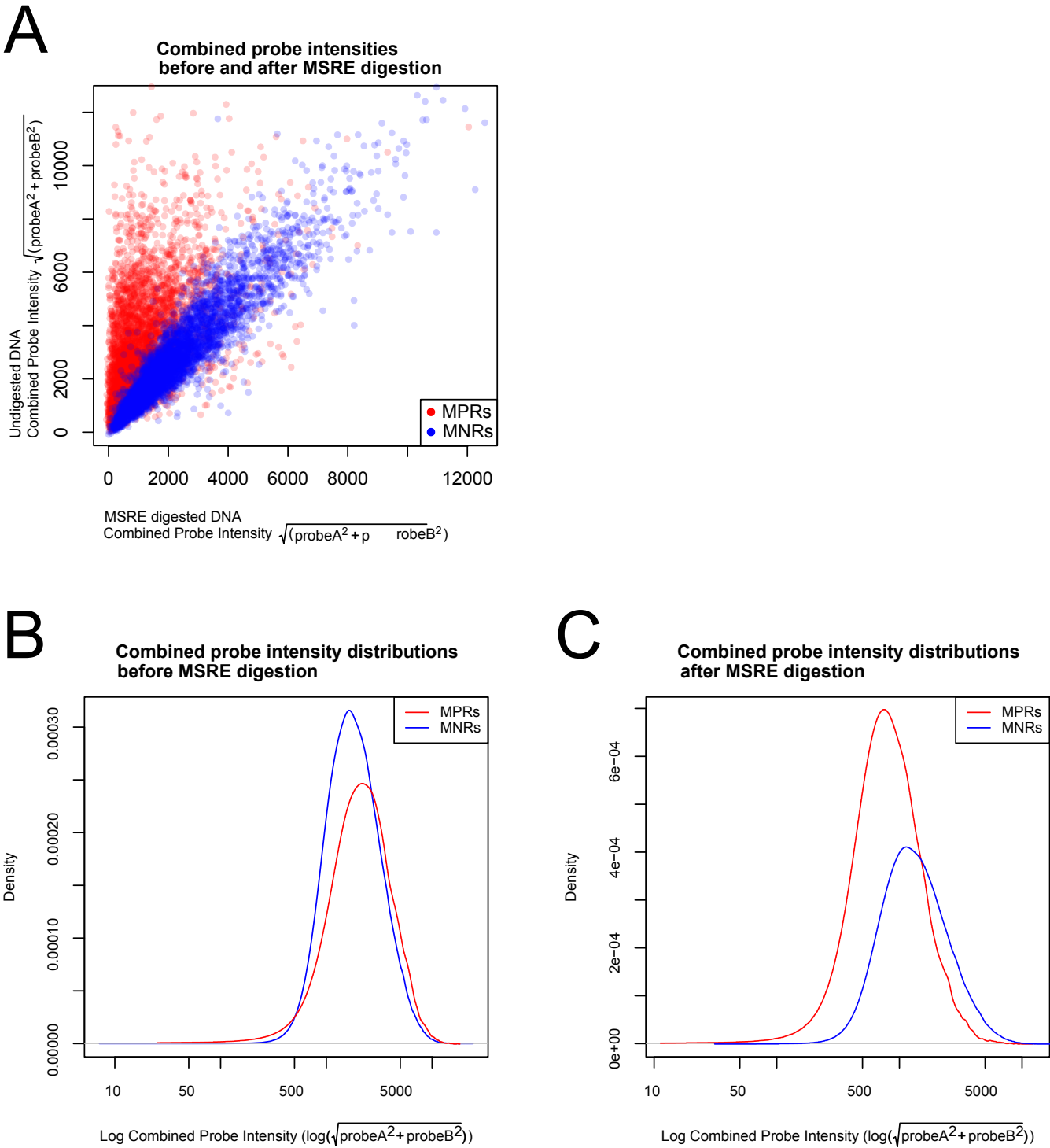

Supplement: Figure S11 — MPRs show lower combined probe intensities relative to MNRs after MSRE digest. Scatter plot (top panel) and density plots (bottom panels) of total probe intensities before (top panel and bottom left panel) and after MSRE digestion (top panel and bottom right panel) for amplicons with (MPRs - in red) and without (MNRs - in blue) MSRE sites in an unmethylated control sample. MPRs show a pronounced shift to lower intensities after MSRE digestion. (PDF) [file pone.0098464.s011.pdf]

Figure S12

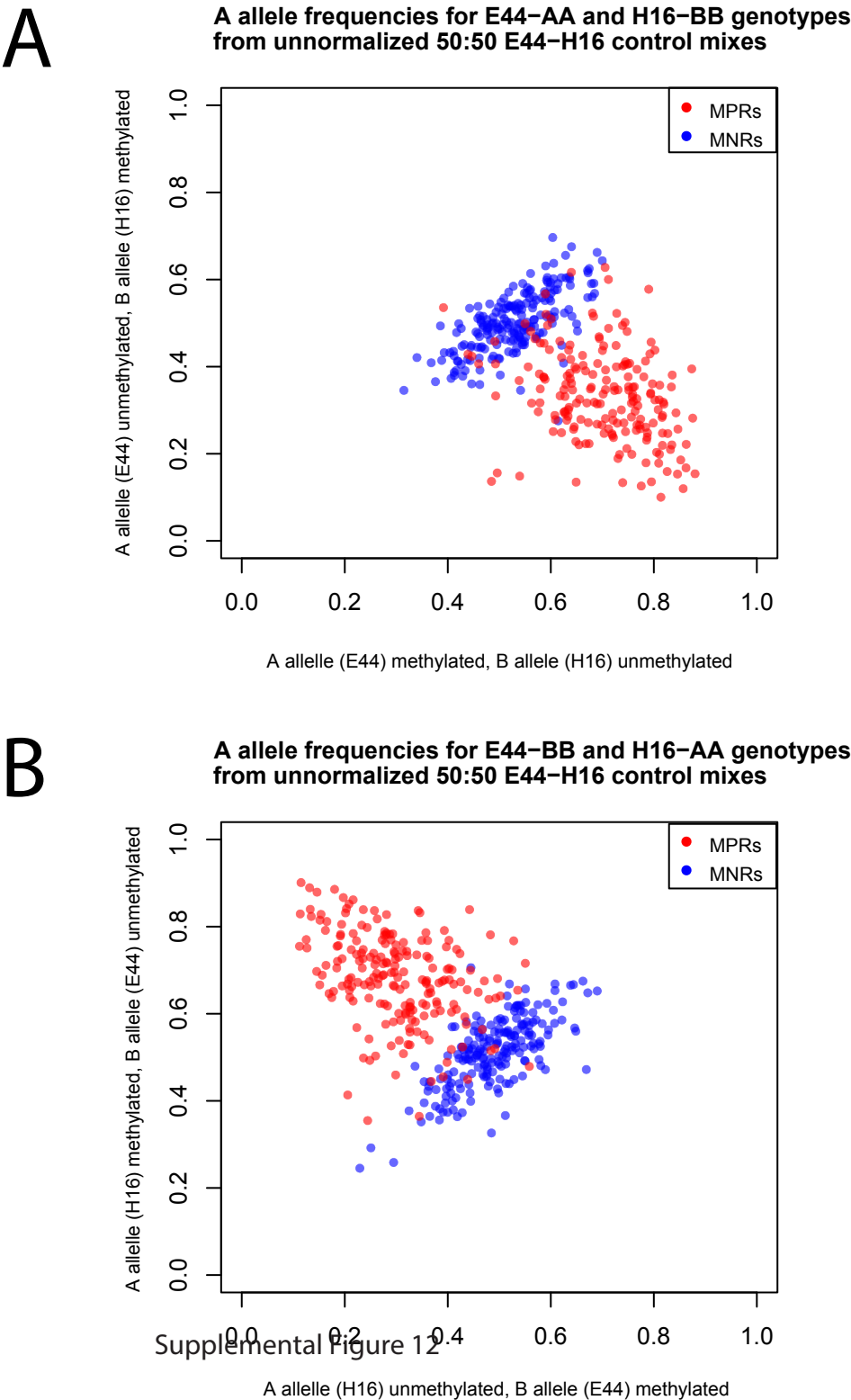

Supplement: Figure S12 — Allele frequencies vary with MSRE digest for MPRs but not MNRs in control methylation mixes. Scatter plots of A allele frequencies (probe A intensity/(probe A intensity + probe B intensity) for amplicons with (MPRs - in red) and without (MNRs - in blue) MSRE sites from two 50∶50 E44-H16 control mix samples, one where one sample has H16 methylated and E44 unmethylated (y-axis) and the other H16 unmethylated and E44 methylated (x-axis). In the top panel, MPRs where E44 contributes the A allele are shown and in the bottom panel, E44 contributes the B allele. MSRE digest does not change the distribution for MNRs but shifts those of MPRs towards the axis of the mix with the methylated A allele. (PDF) [file pone.0098464.s012.pdf]

Figure S14

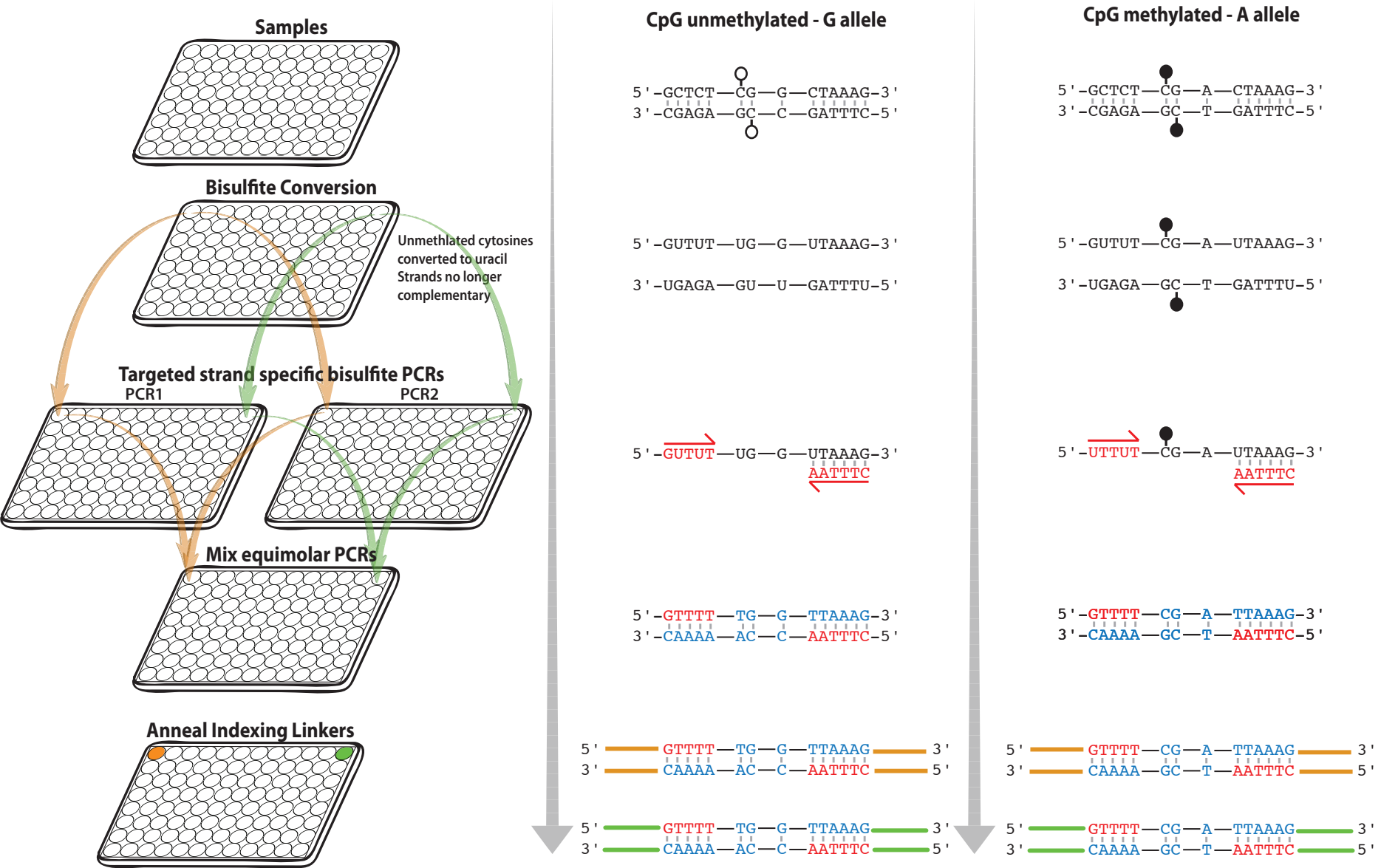

Supplement: Figure S14 — Bisulfite PCR methods. A simplified representation of the bisulfite PCR sequencing assays. Sample DNA was plated in 96 well plates, bisulfite converted and aliquoted into separate 96 well plates for each amplification target (left panel). Each 96 well plate was subjected to bisulfite-specific PCR (BSP), amplifications were combined in equimolar amounts by individual into a single 96 well and bar-coded before sequencing. Primers were designed to flank both the target SNP (here a G/A SNP on the top strand, middle and right panels) and CpG, amplify only bisulfite converted DNA and be strand specific in a manner that allowed allele identification after bisulfite conversion. Example amplifications for a scenario where the G-allele is not associated with CpG methylation (middle panel) and the A allele is associated with a methylated CpG (right panel) are shown. Note that strand specific amplification of the C/T SNP on the bottom strand would not allow allele identification after bisulfite conversion. (PDF) [file pone.0098464.s014.pdf]

Figure S15

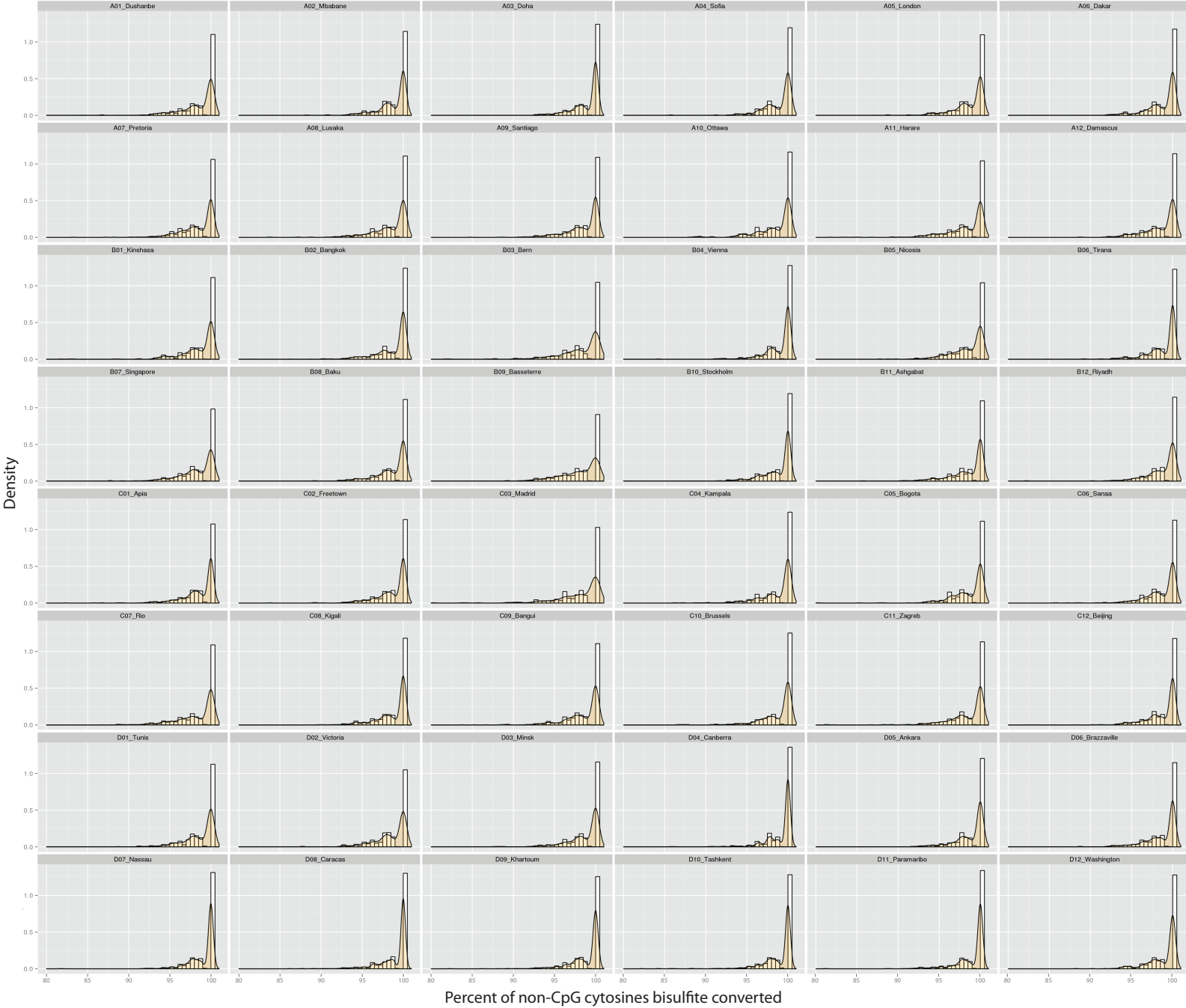

Figure S15 (continued)

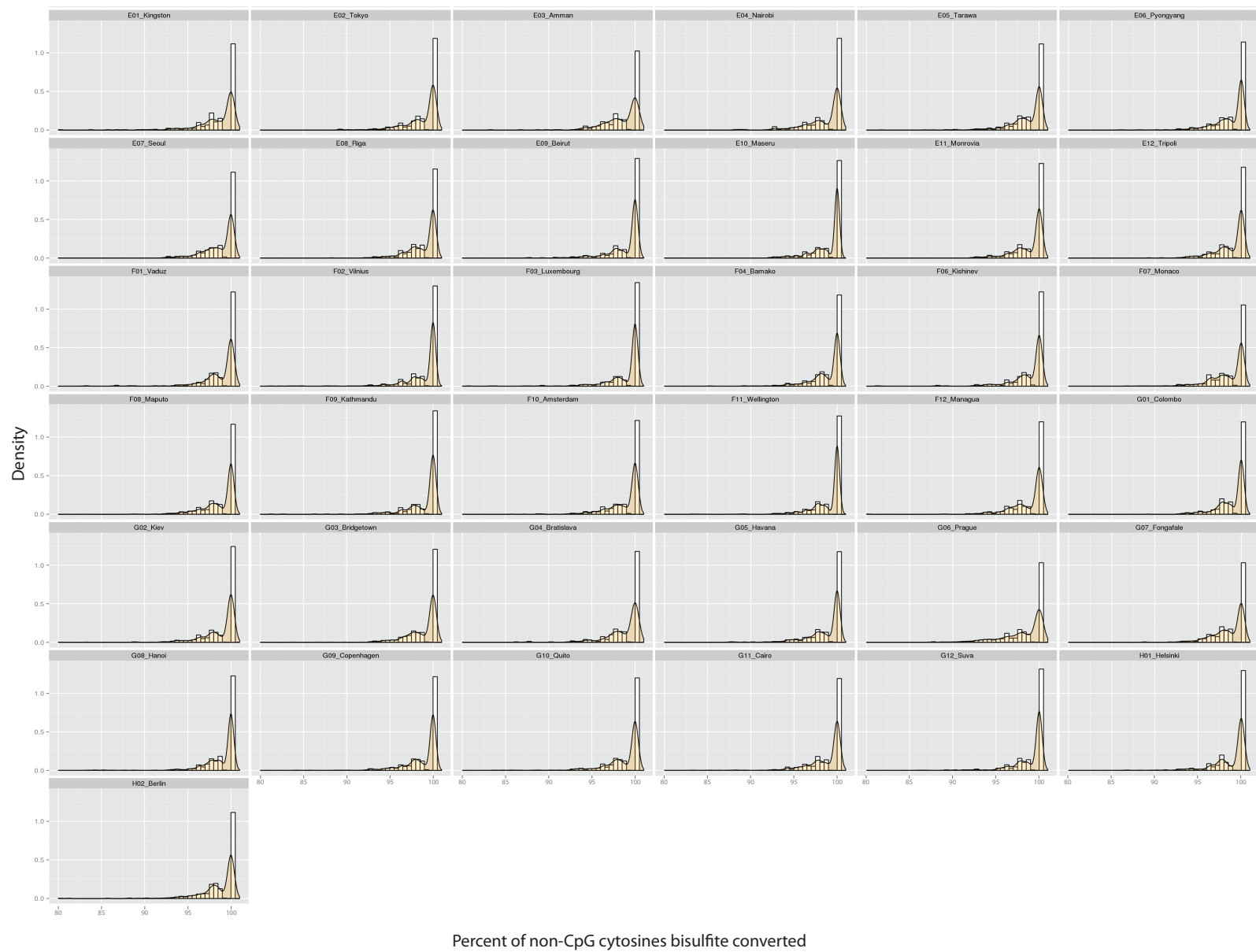

Supplement: Figure S15 — Bisulfite conversion rates. Distributions of median bisulfite conversion rates for all non-CpG cytosines in each amplicon are shown for each sample. Both histograms (grey) and density plots (orange) are shown. (PDF) [file pone.0098464.s015.pdf]

Figure S16

A

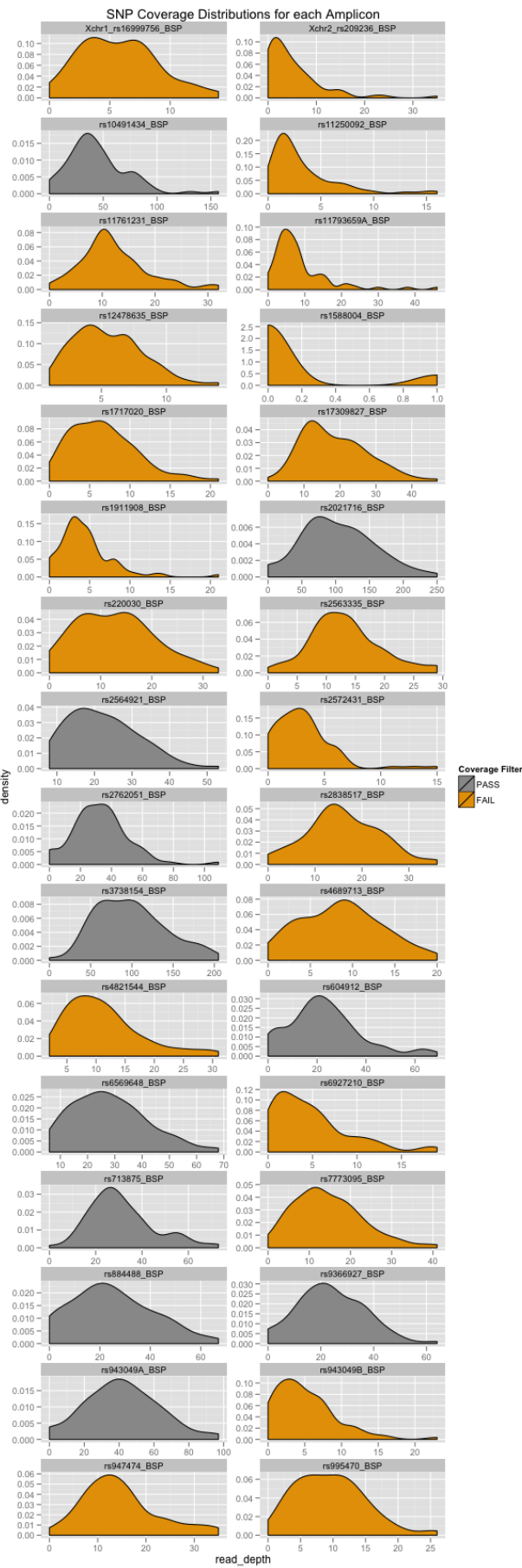

B

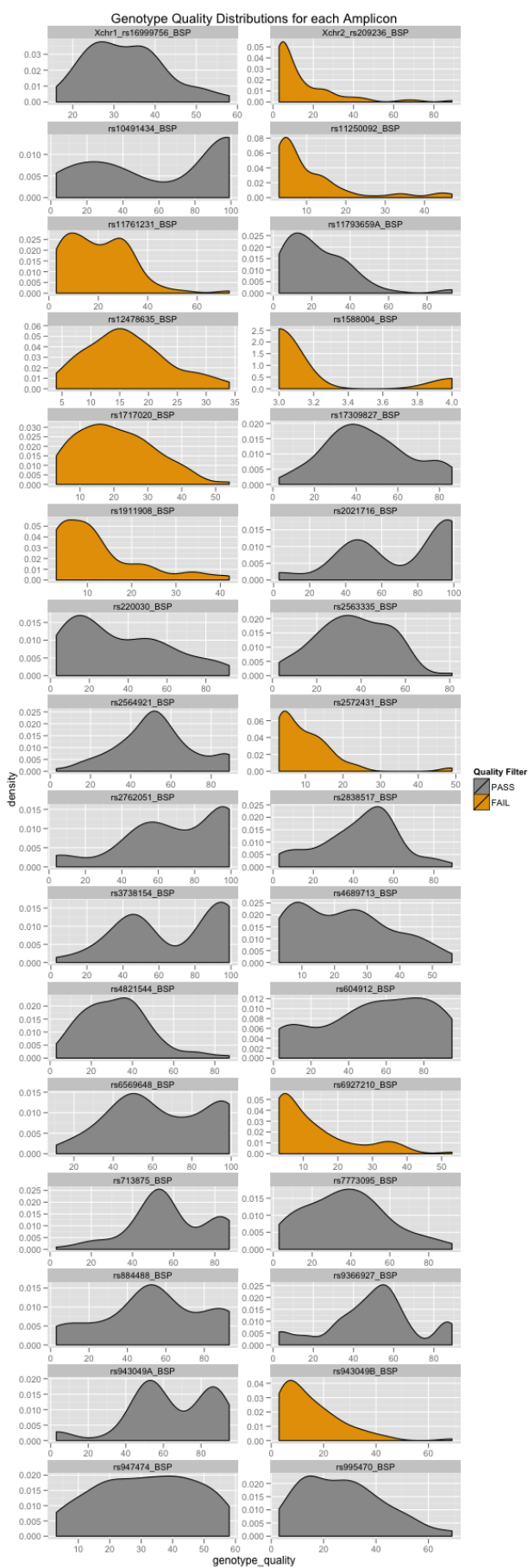

Figure S16 (continued)

C

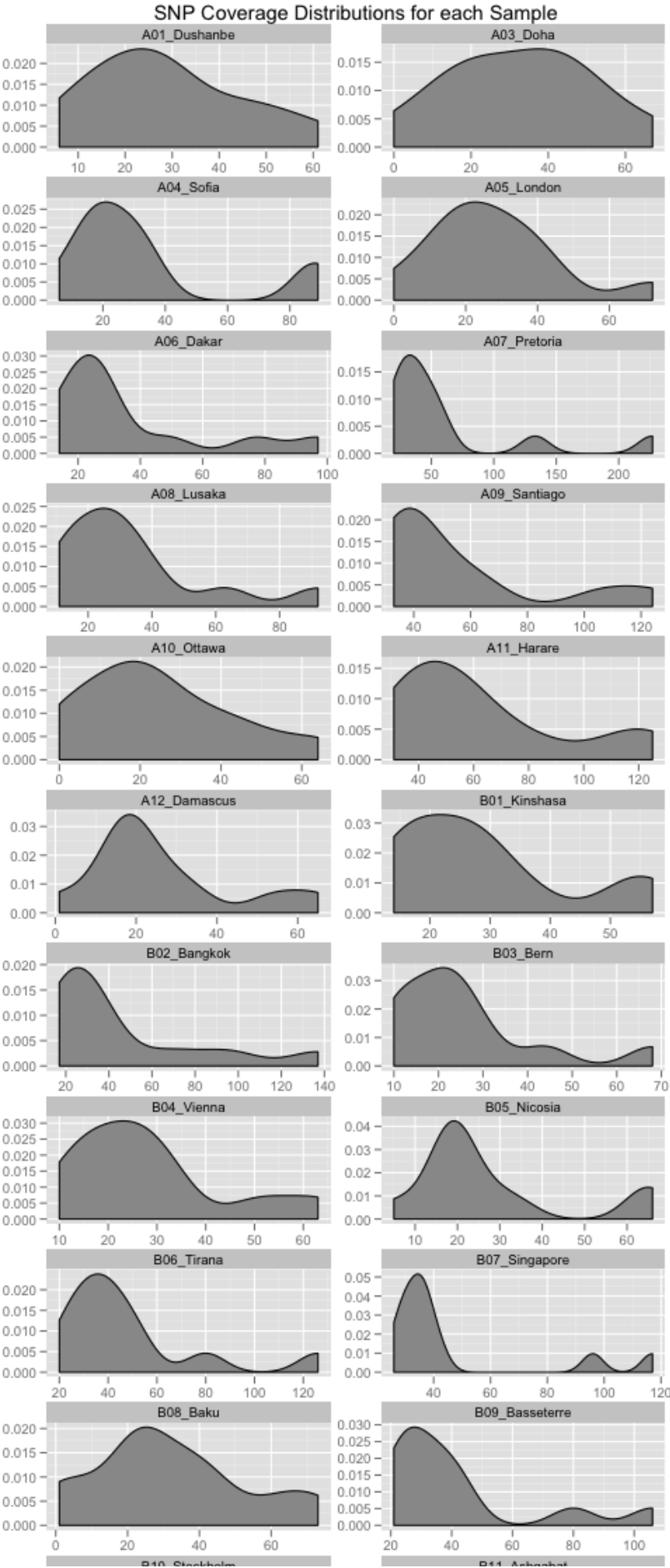

Figure S16 (continued)

D

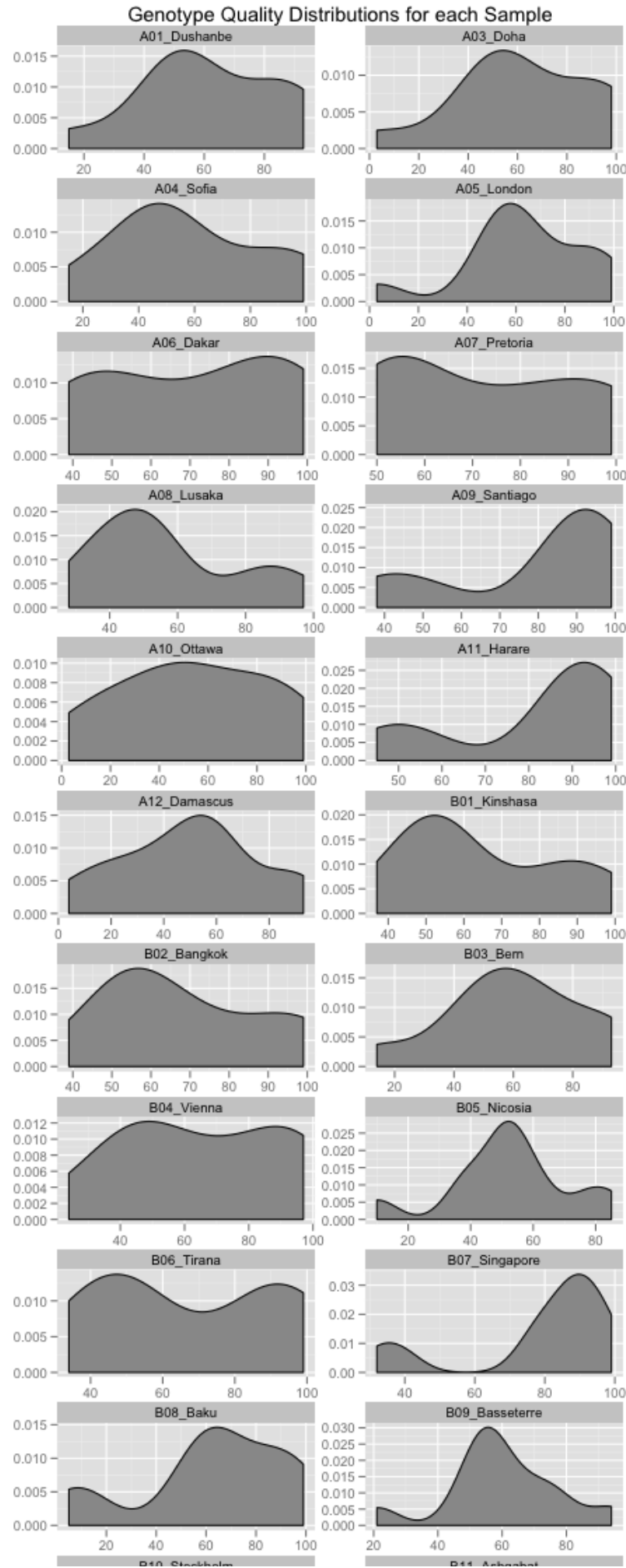

Supplement: Figure S16 — Amplicon and sample genotype coverage and qualities. Target SNP read coverage (A and C) and genotype quality (B and D) distributions across all samples for each amplicon (A and B) or across all amplicons for each sample (C and D) are shown. Amplicons failed this test (orange filled distributions) if the majority of samples had either less than 20-fold coverage or genotype qualities below 20. Amplicons that passed these quality filters (grey filled distributions in both panels A and B) were carried forward. Similarly, samples failed these tests if the majority of amplicons had either less than 20-fold coverage or genotype qualities below 20. (PDF) [file pone.0098464.s016.pdf]

Figure S17

A

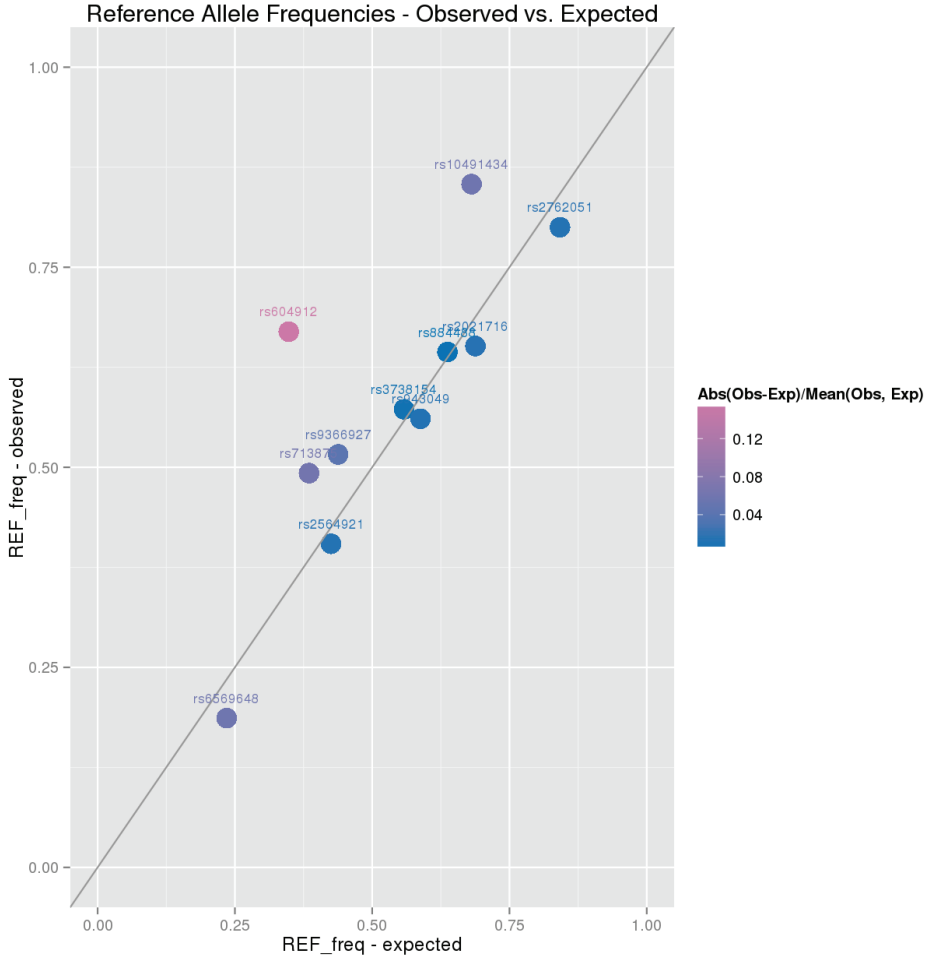

B

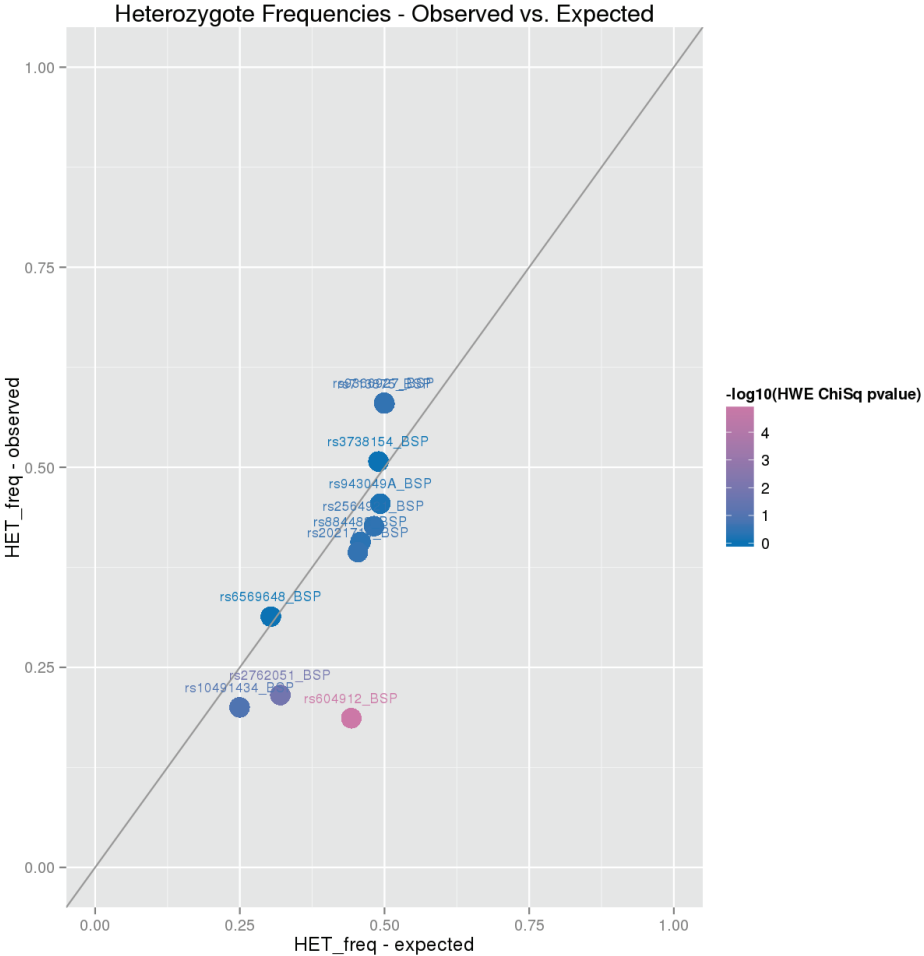

Supplement: Figure S17 — Hardy Weinberg Equilibrium and allele frequency based amplicon filtering. Reference allele frequencies and heterozygote frequencies for assayed variants. A) Observed versus expected (HapMap) reference allele frequencies are shown. Colors of plotted data reflect the relative mean difference of the observed minor allele frequency from the expected CEU minor allele frequency (HapMap release 27). B) Observed versus expected heterozygote frequencies as based on observed allele frequencies. Colors of plotted data reflect the p-values from of an exact test as defined by [49]. (PDF) [file pone.0098464.s017.pdf]
