# Supplementary material for: Allele-Specific Methylation Occurs at Genetic Variants Associated with Complex Disease
Source: PLoS One. 2014 Jun 9;9(6):e98464. doi: 10.1371/journal.pone.0098464 (PMC4049588; doi:10.1371/journal.pone.0098464)

Figure S4

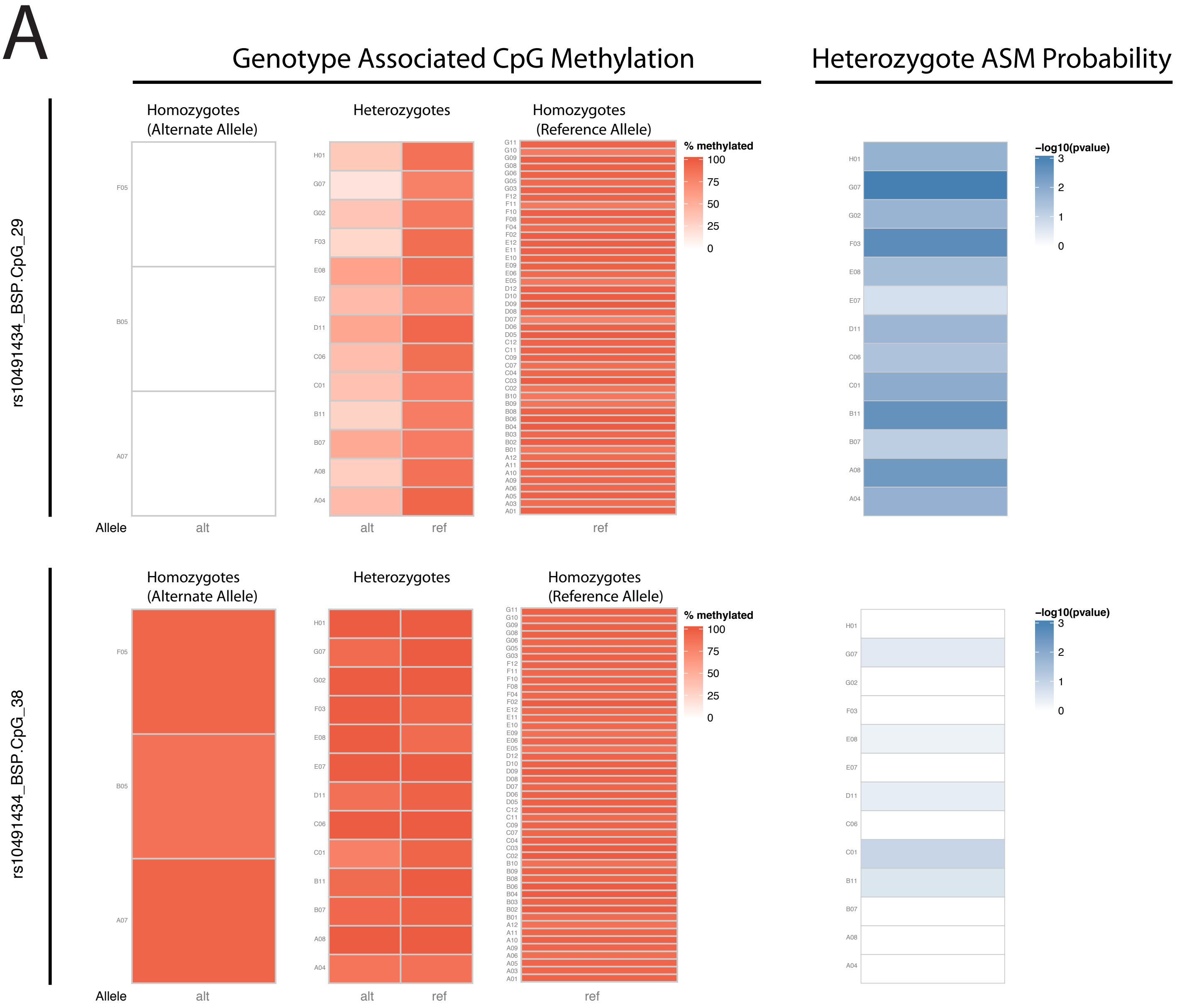
A
Genotype Associated CpG Methylation
Heterozygote ASM Probability
rs10491434_BSP.CpG_29
Homozygotes (Alternate Allele)
Heterozygotes
Homozygotes (Reference Allele)
% methylated
100
75
50
25
0
−log10(pvalue)
3
2
1
0
Allele
alt
alt
ref
ref
rs10491434_BSP.CpG_38
Homozygotes (Alternate Allele)
Heterozygotes
Homozygotes (Reference Allele)
% methylated
−log10(pvalue)
Allele
alt
alt
ref
ref

Figure S4 (continued)

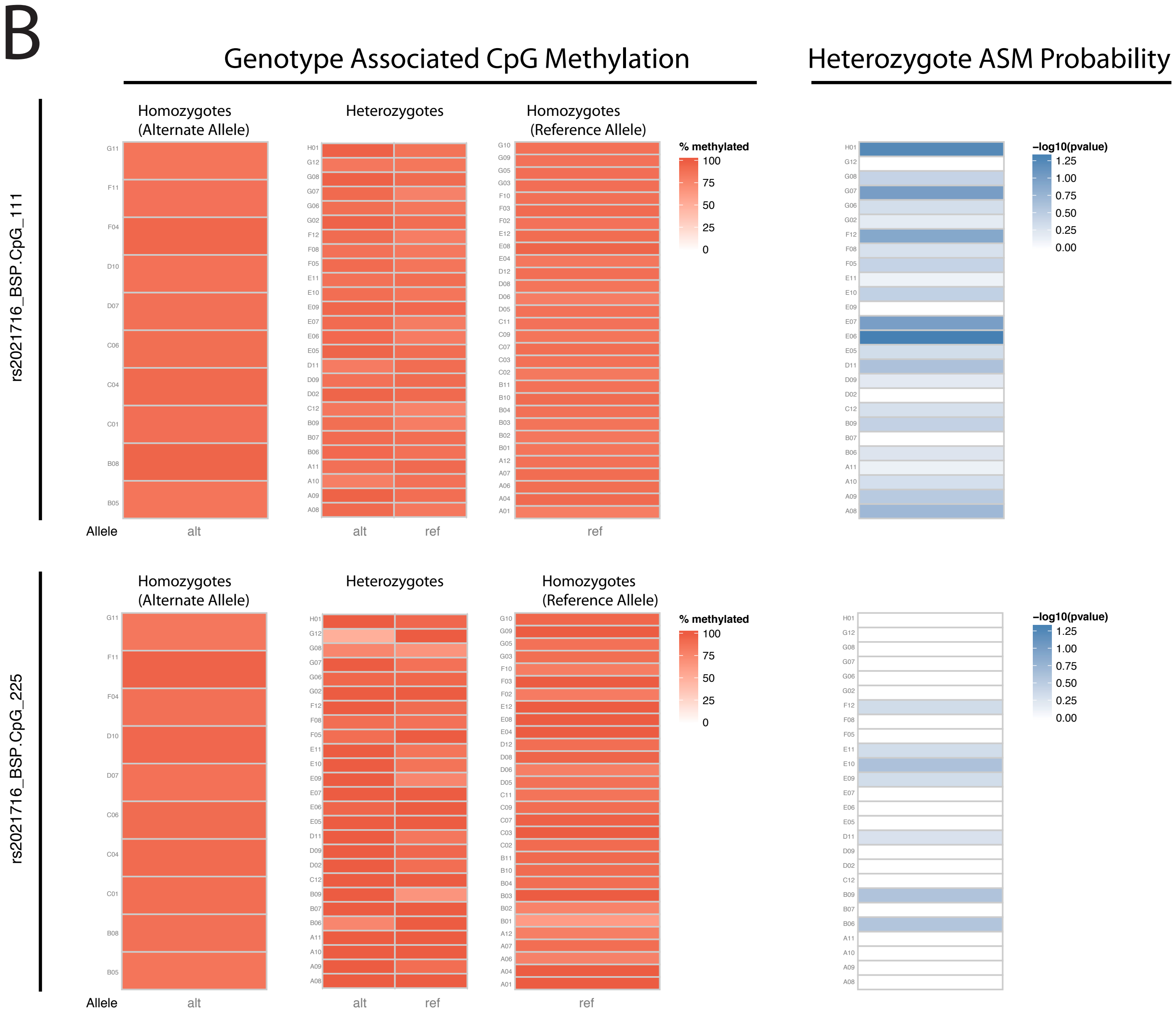

Figure S4 (continued)

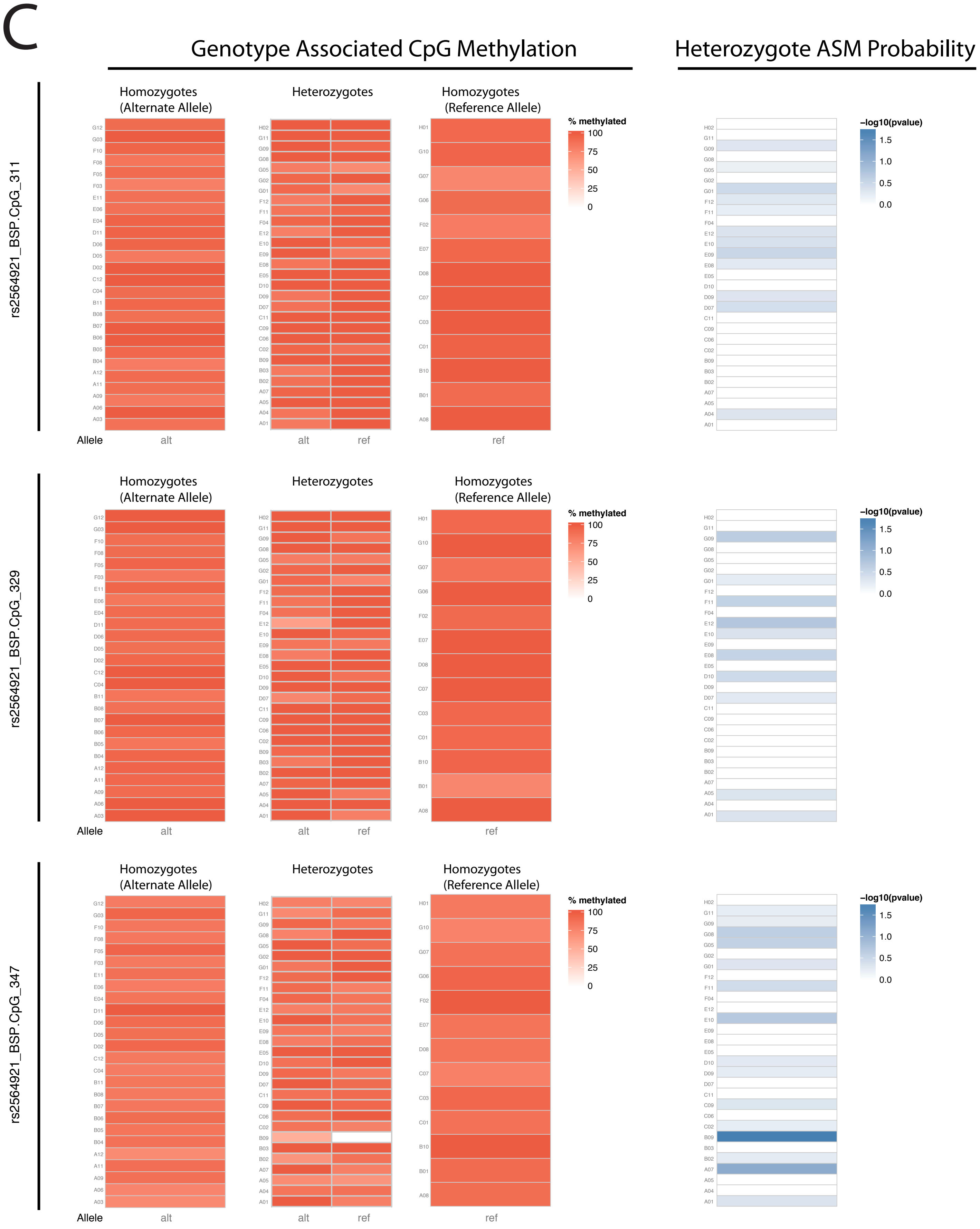

Figure S4 (continued)

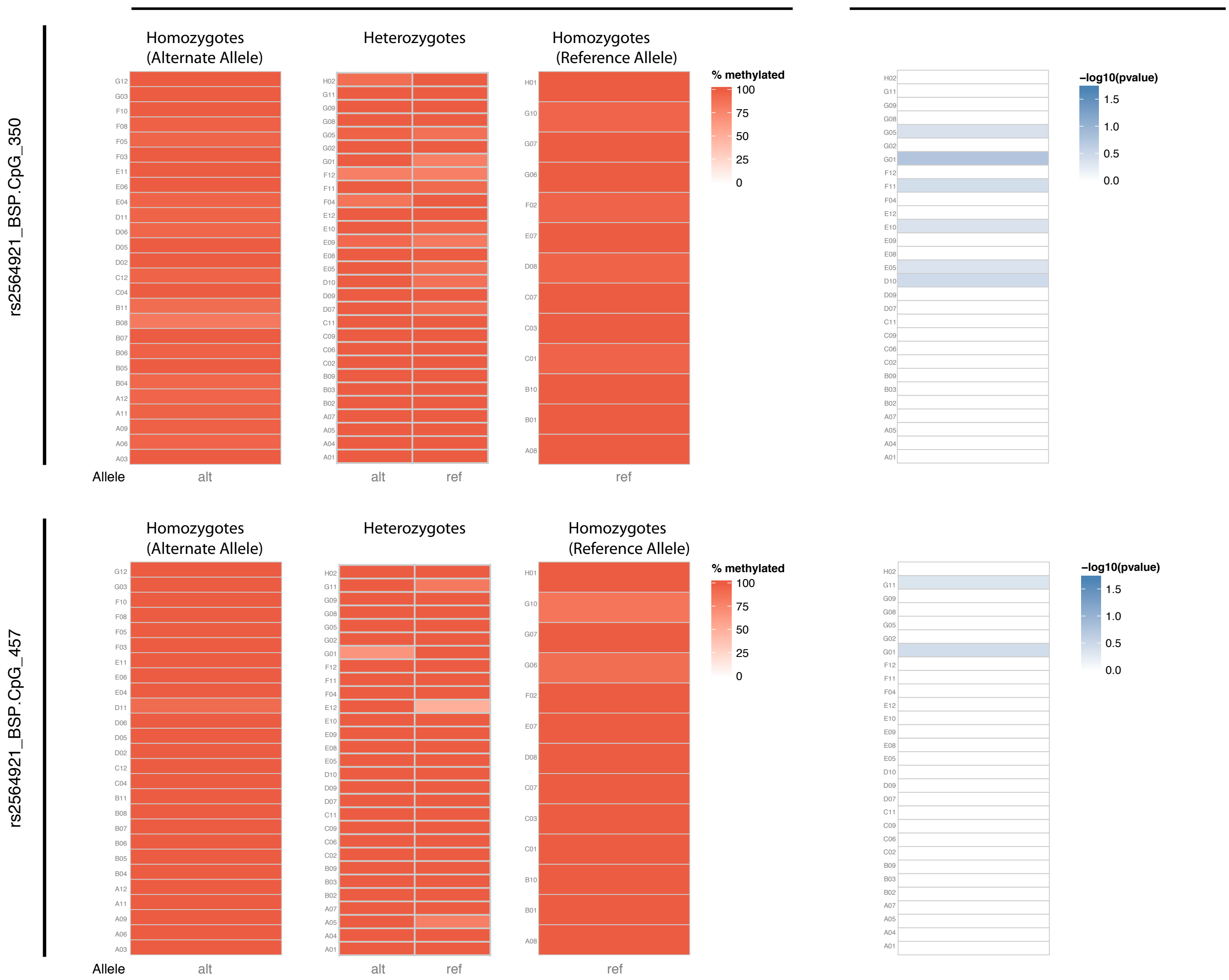

Figure S4 (continued)

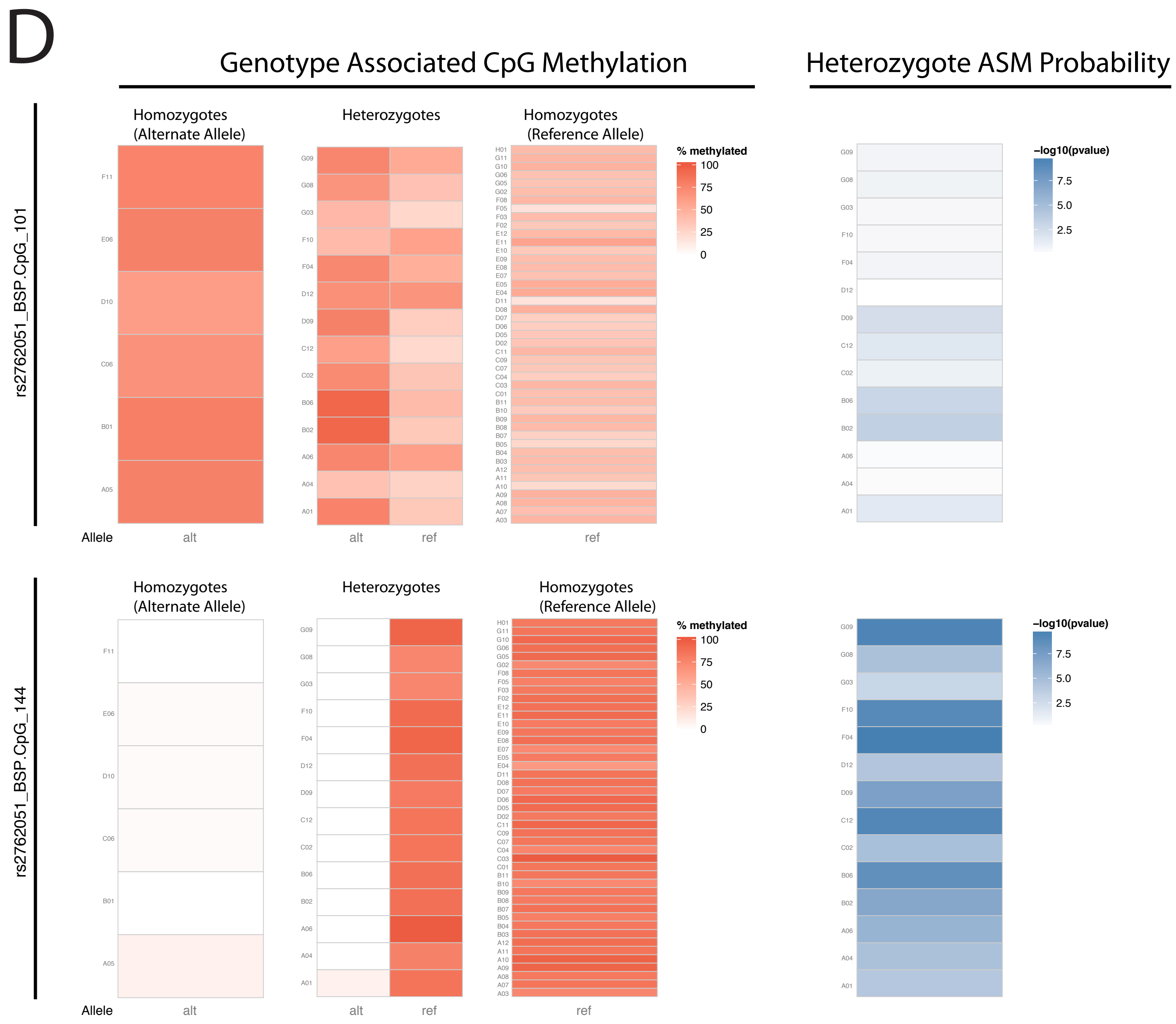

Supplemental Figure 9 (continued)

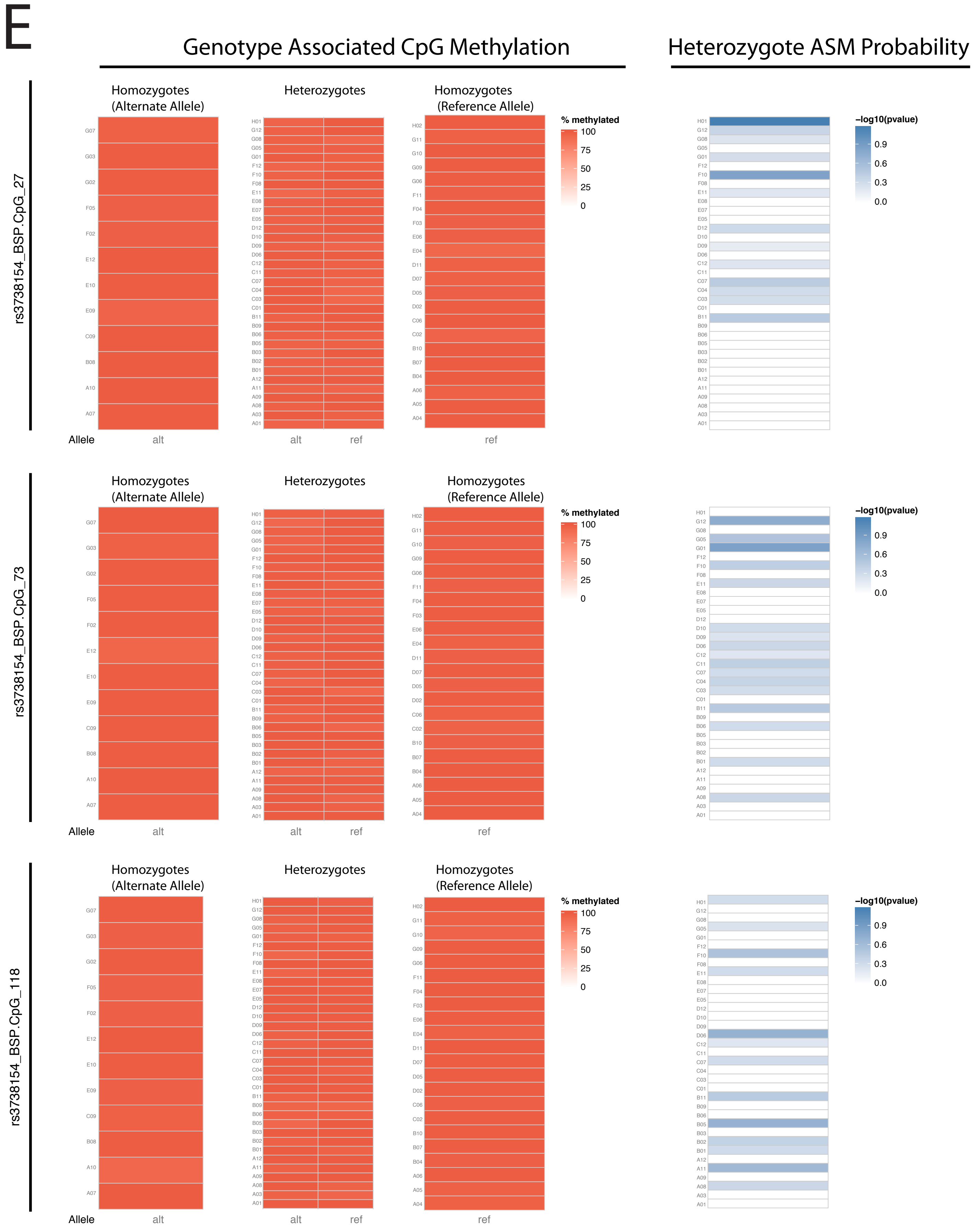

Figure S4 (continued)

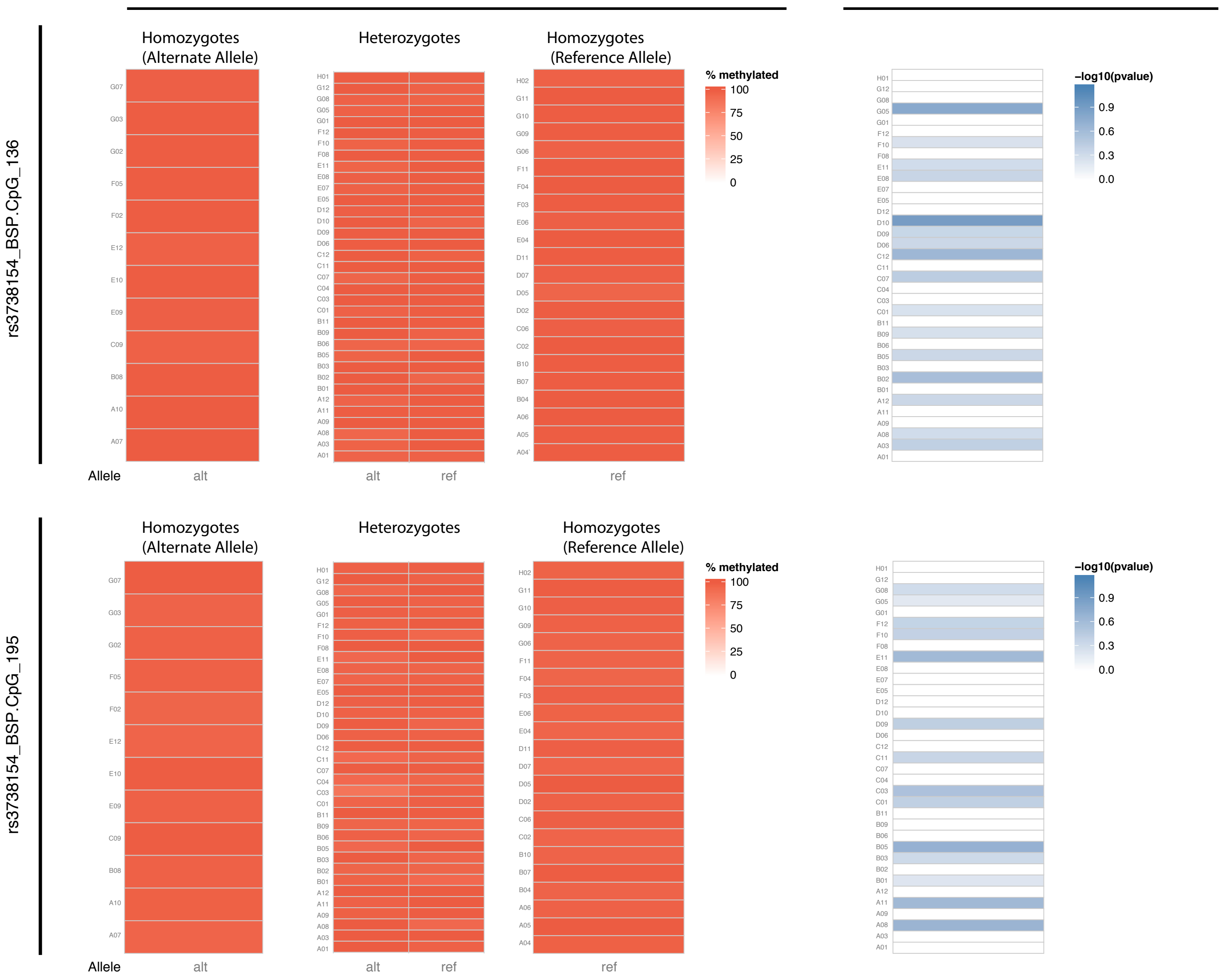

Figure S4 (continued)

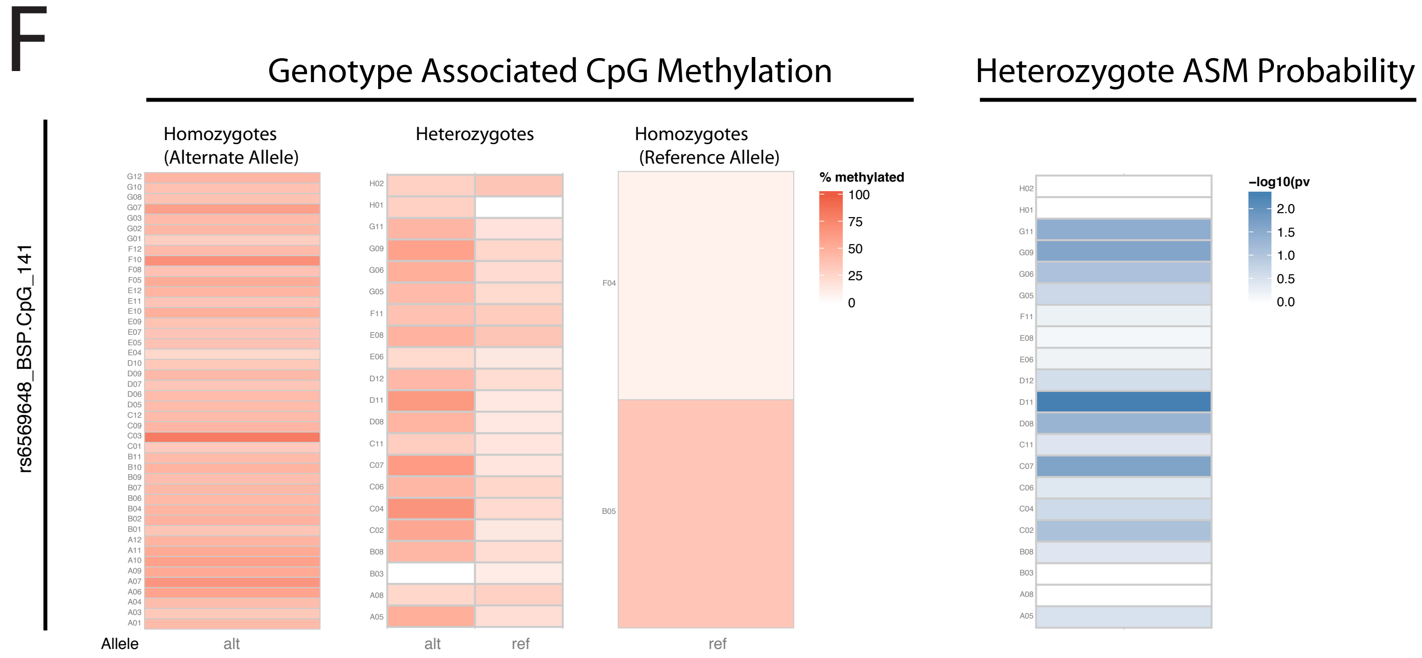

Figure S4 (continued)

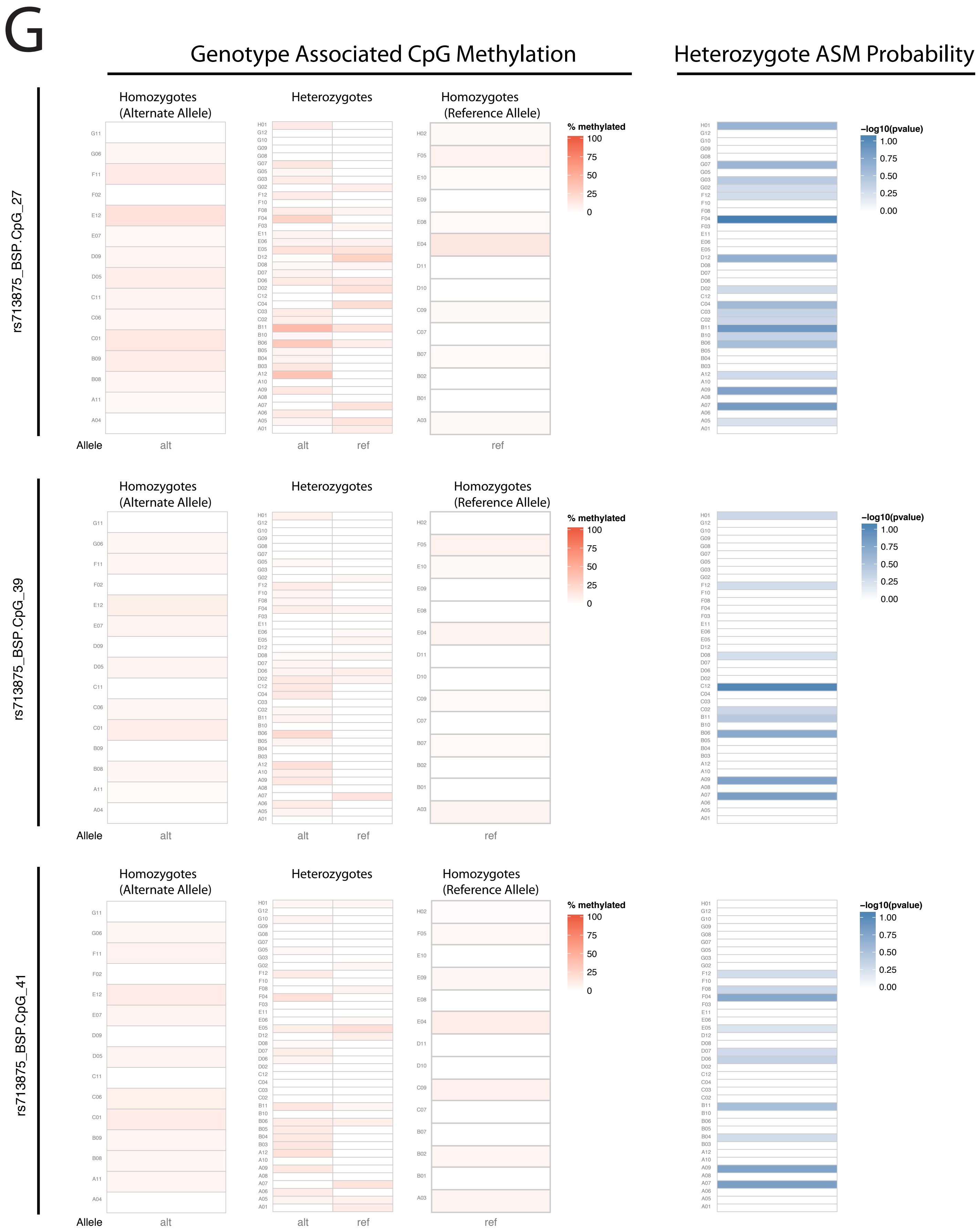

Supplemental Figure 9 (continued)

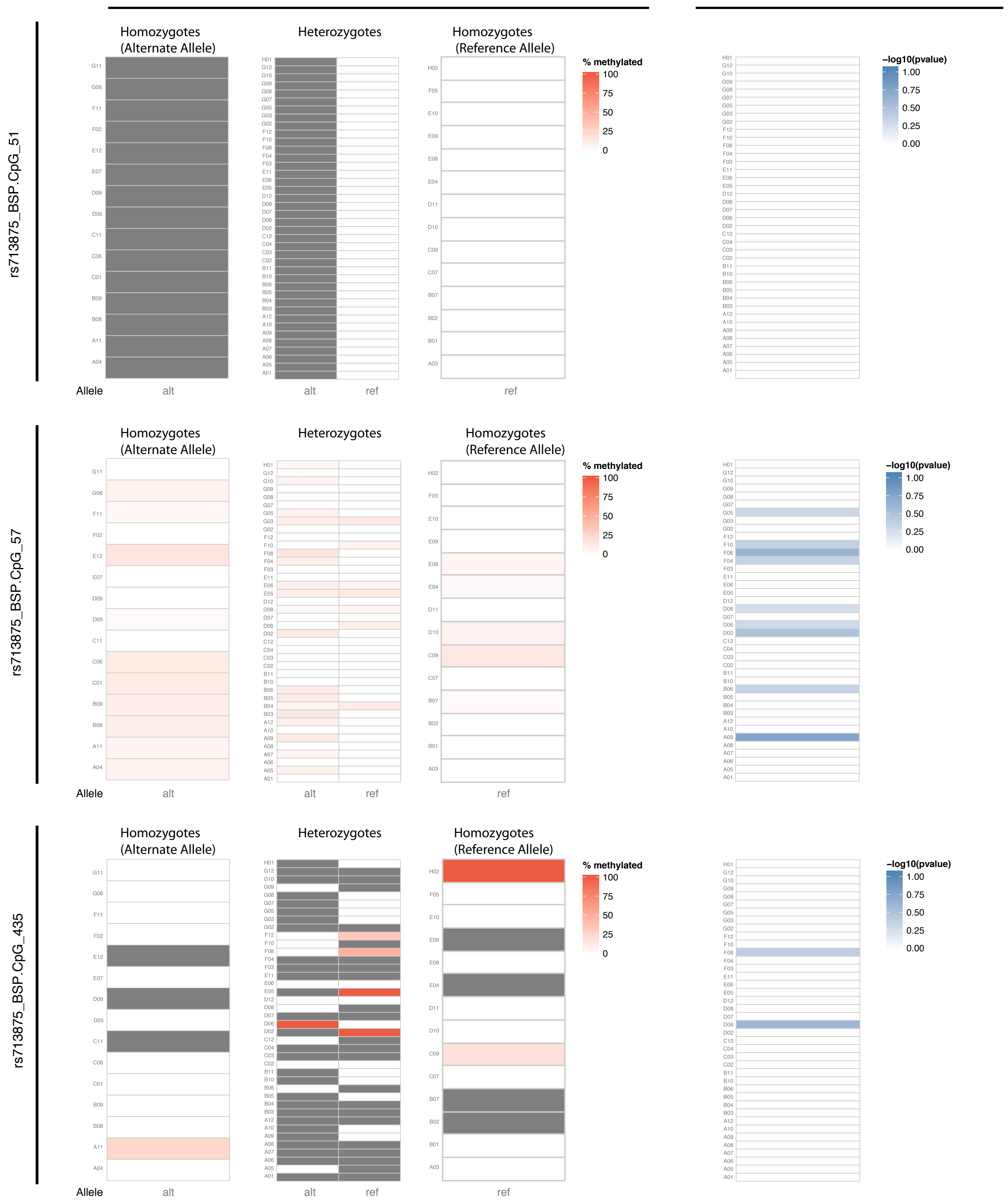

Figure S4 (continued)

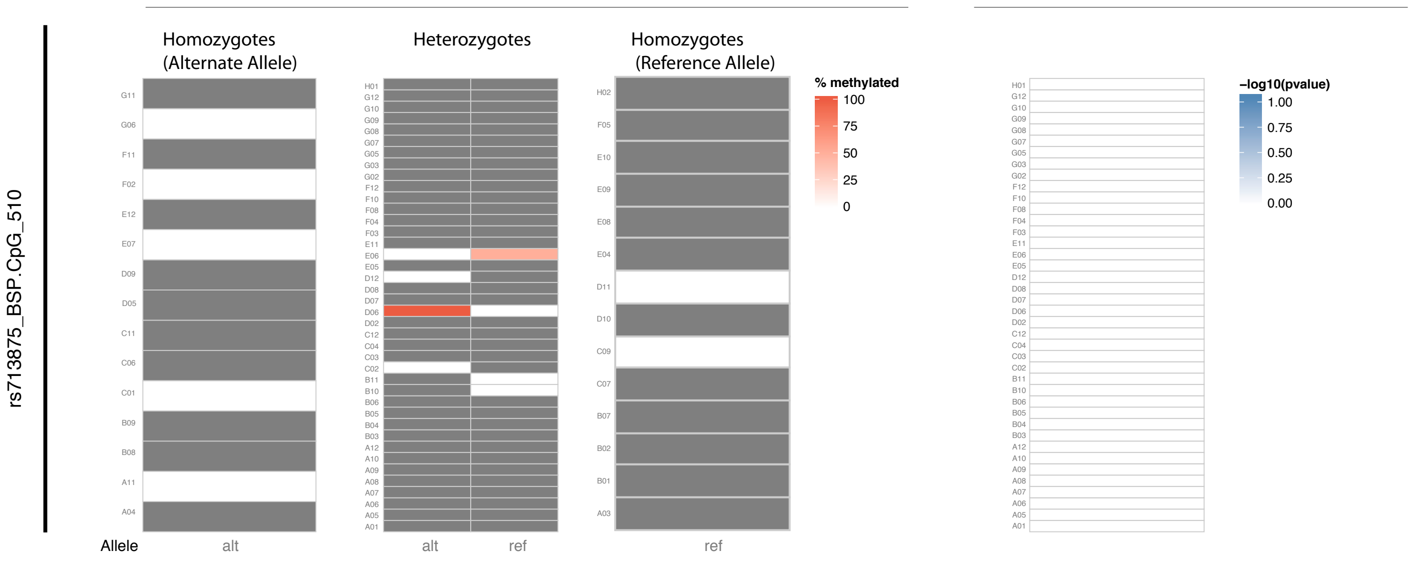

Supplemental Figure 9 (continued)

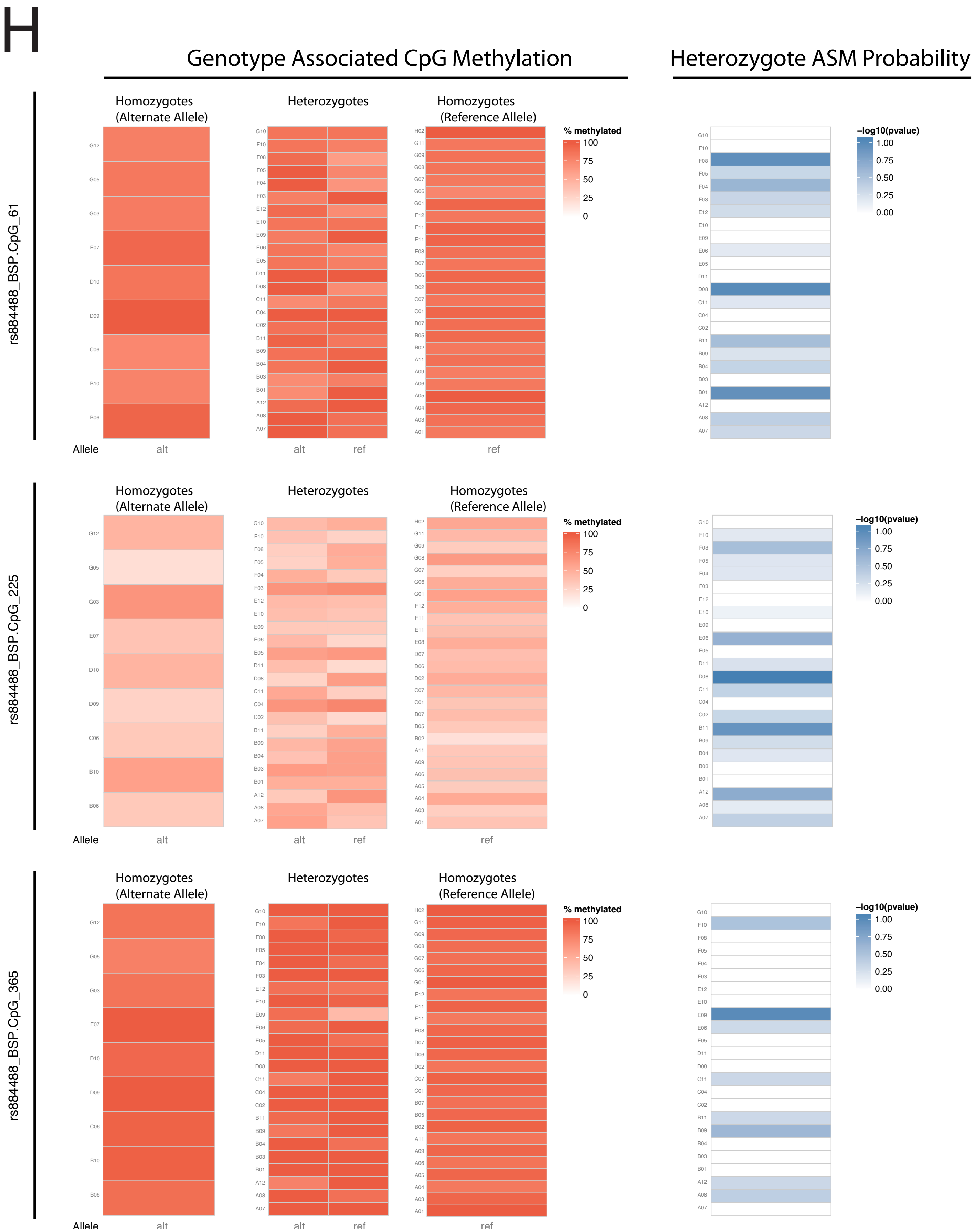

Figure S4 (continued)

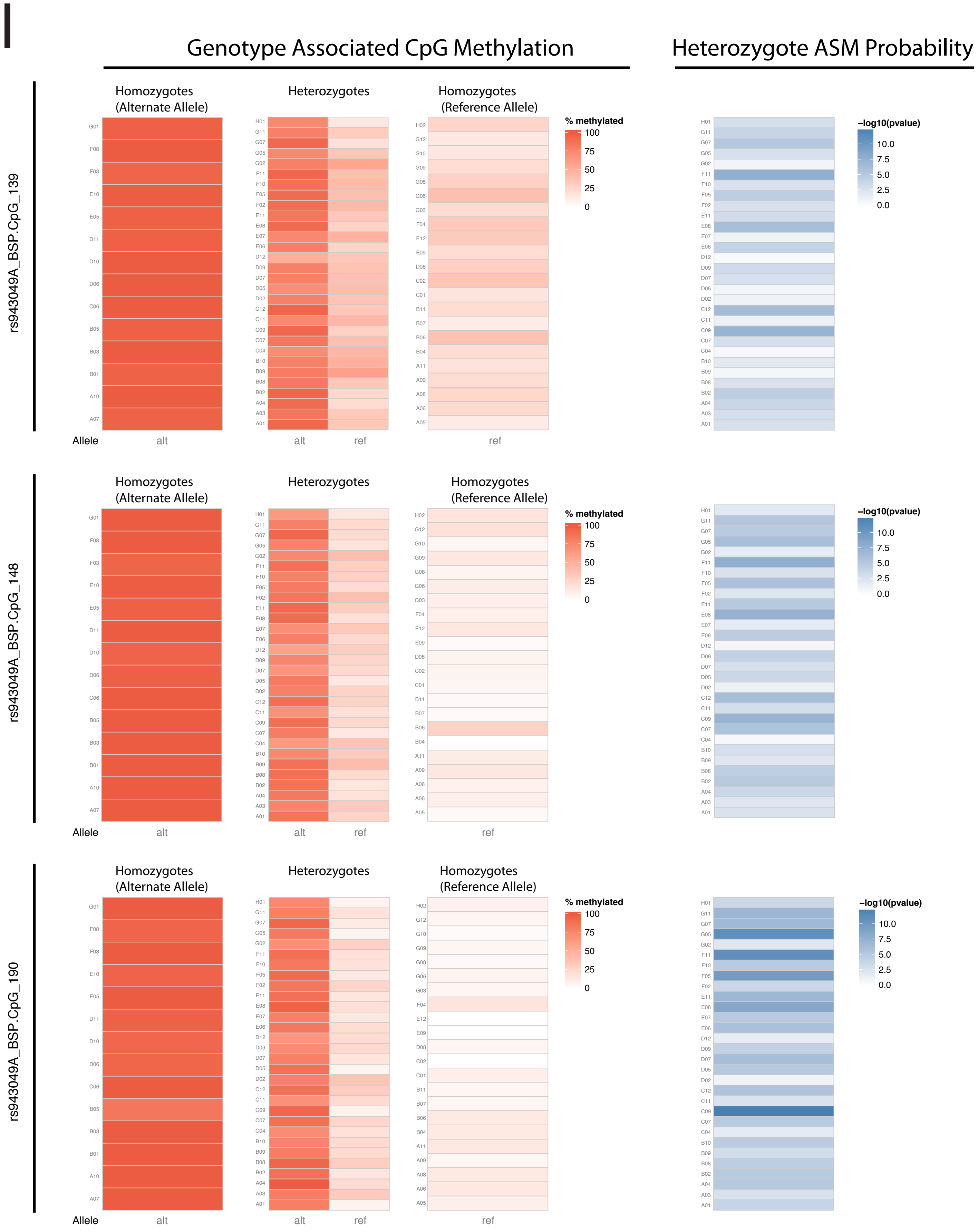

Supplemental Figure 9 (continued)

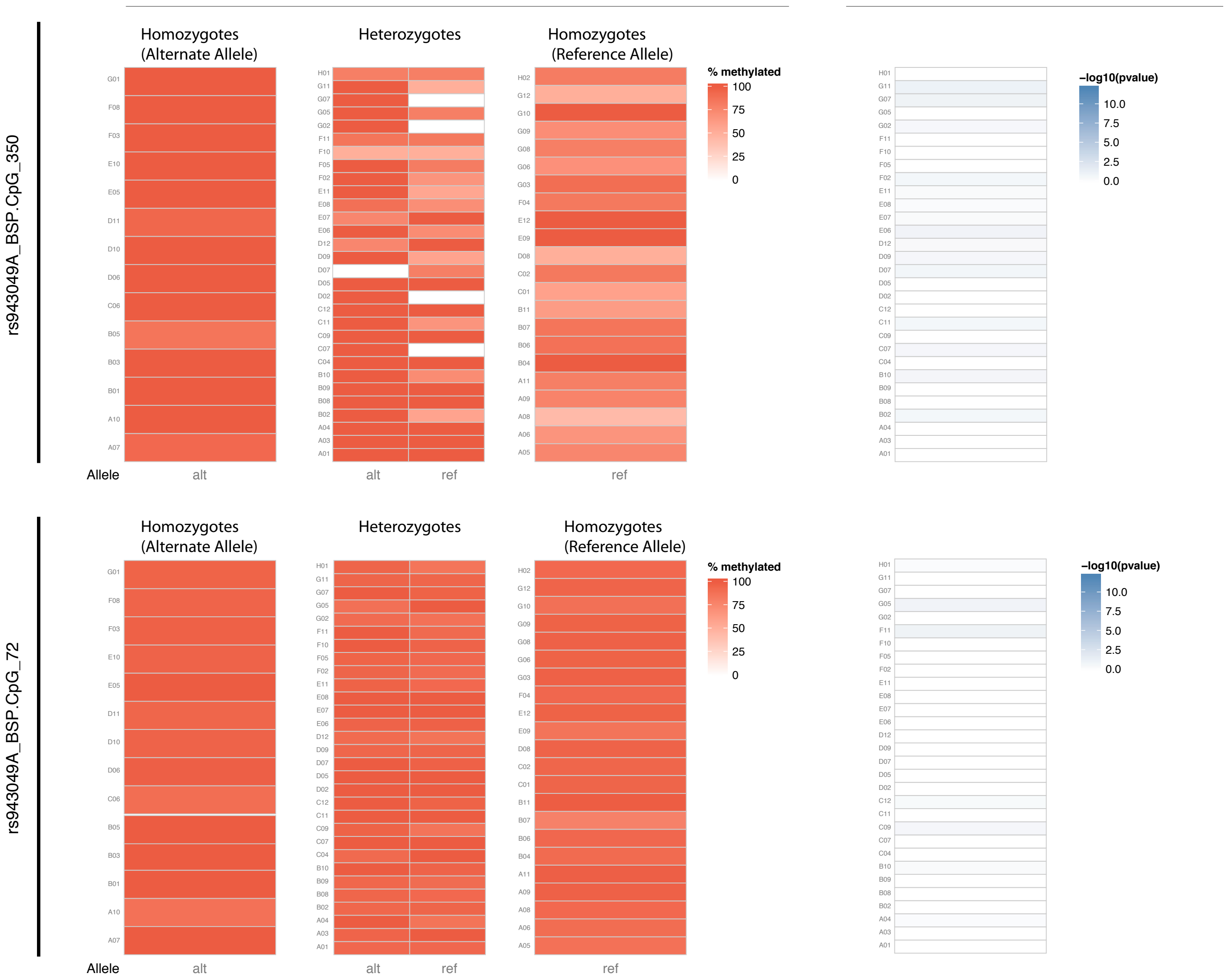

Figure S4 (continued)

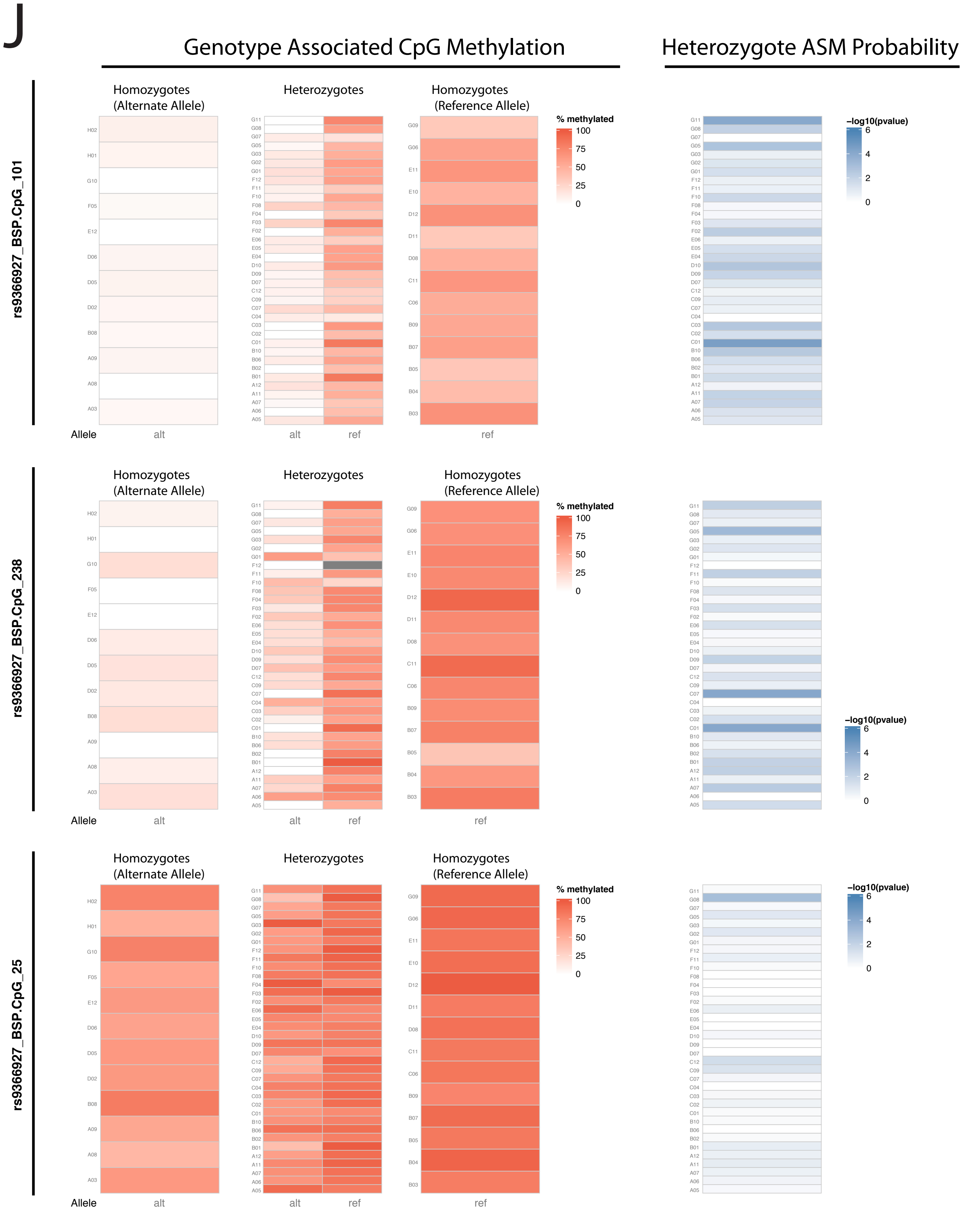

Supplemental Figure 9 (continued)

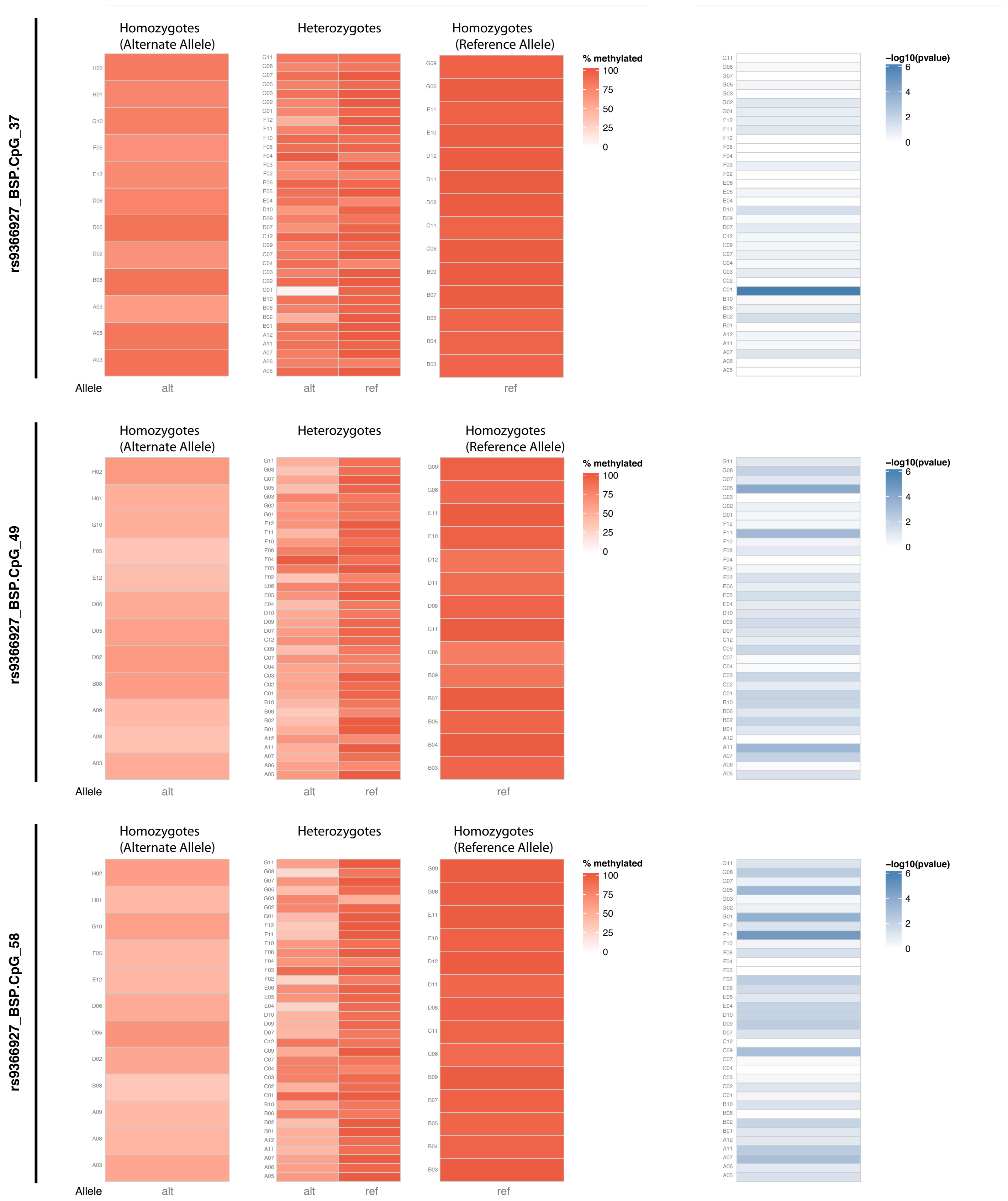

Figure S4 (continued)

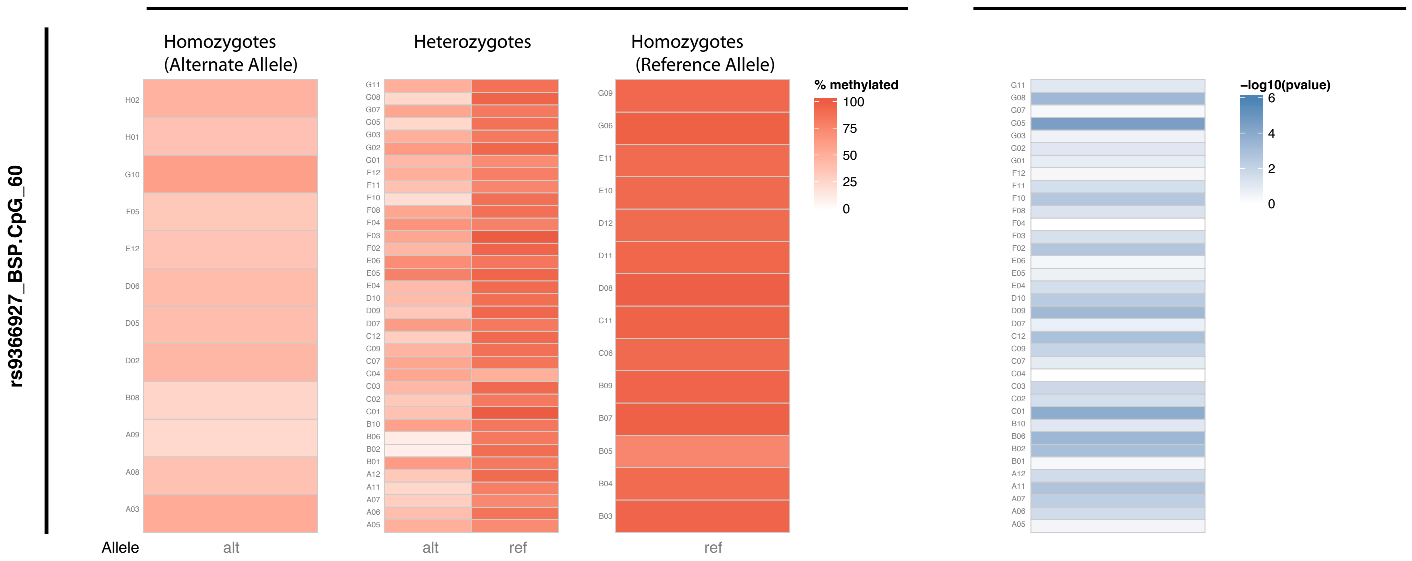

Supplement: Figure S4 — Assessing allele-specific methylation in an independent population. Results for all individual CpGs of the ten amplicons (A - rs10491434_BSP; B - rs2021716_BSP; C - rs2564921_BSP; D - rs2762051_BSP; E - rs3738154_BSP; F - rs6569648_BSP; G - rs713875_BSP; H - rs884488_BSP; I - rs943049A_BSP; J - rs9366927_BSP) are shown. Heatmaps show percent methylation status for single CpGs within the amplified regions of MPRs for all samples (alternate allele homozygotes (1st column), heterozygotes (2nd column) and reference allele homozygotes. (3rd column)); darker red denotes higher methylation percentages within a sample at the CpG. The final column shows the –log10 p-values derived from chi-square tests for association of methylation with one allele; darker blue results show greater evidence of cis-regulated ASM at the CpG in a particular sample. (PDF) [file pone.0098464.s004.pdf]
