# Supplementary material for: Allele-Specific Methylation Occurs at Genetic Variants Associated with Complex Disease
Source: PLoS One. 2014 Jun 9;9(6):e98464. doi: 10.1371/journal.pone.0098464 (PMC4049588; doi:10.1371/journal.pone.0098464)

Figure S5

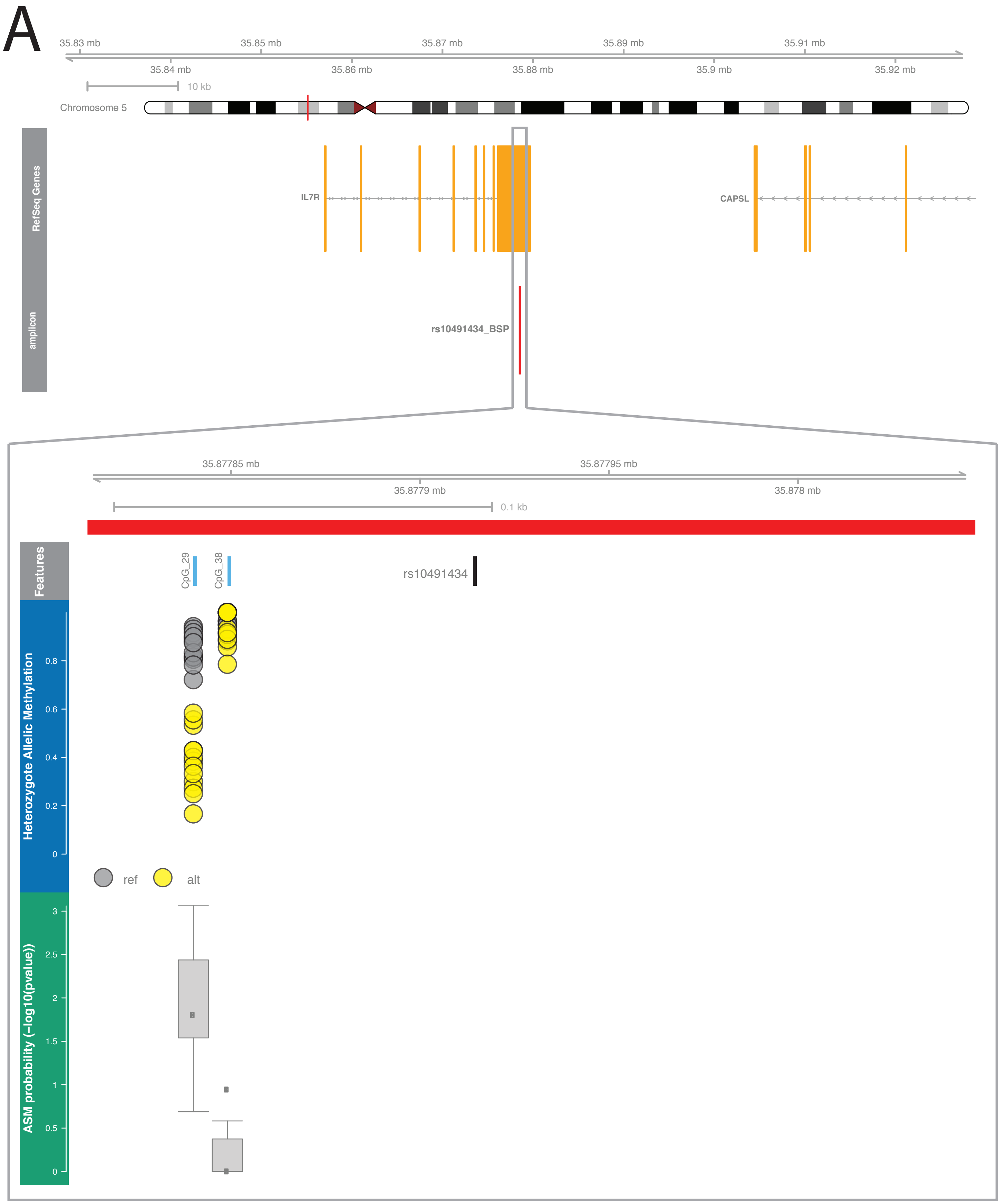
A
35.83 mb
35.85 mb
35.87 mb
35.89 mb
35.91 mb
35.84 mb
35.86 mb
35.88 mb
35.9 mb
35.92 mb
10 kb
Chromosome 5
RefSeq Genes
amplicon
IL7R
CAPSL
rs10491434_BSP
35.87785 mb
35.87795 mb
35.8779 mb
35.878 mb
0.1 kb
Features
CpG_29
CpG_38
rs10491434
Heterozygote Allelic Methylation
ref
alt
ASM probability (−log10(pvalue))

Figure S5 (continued)

B

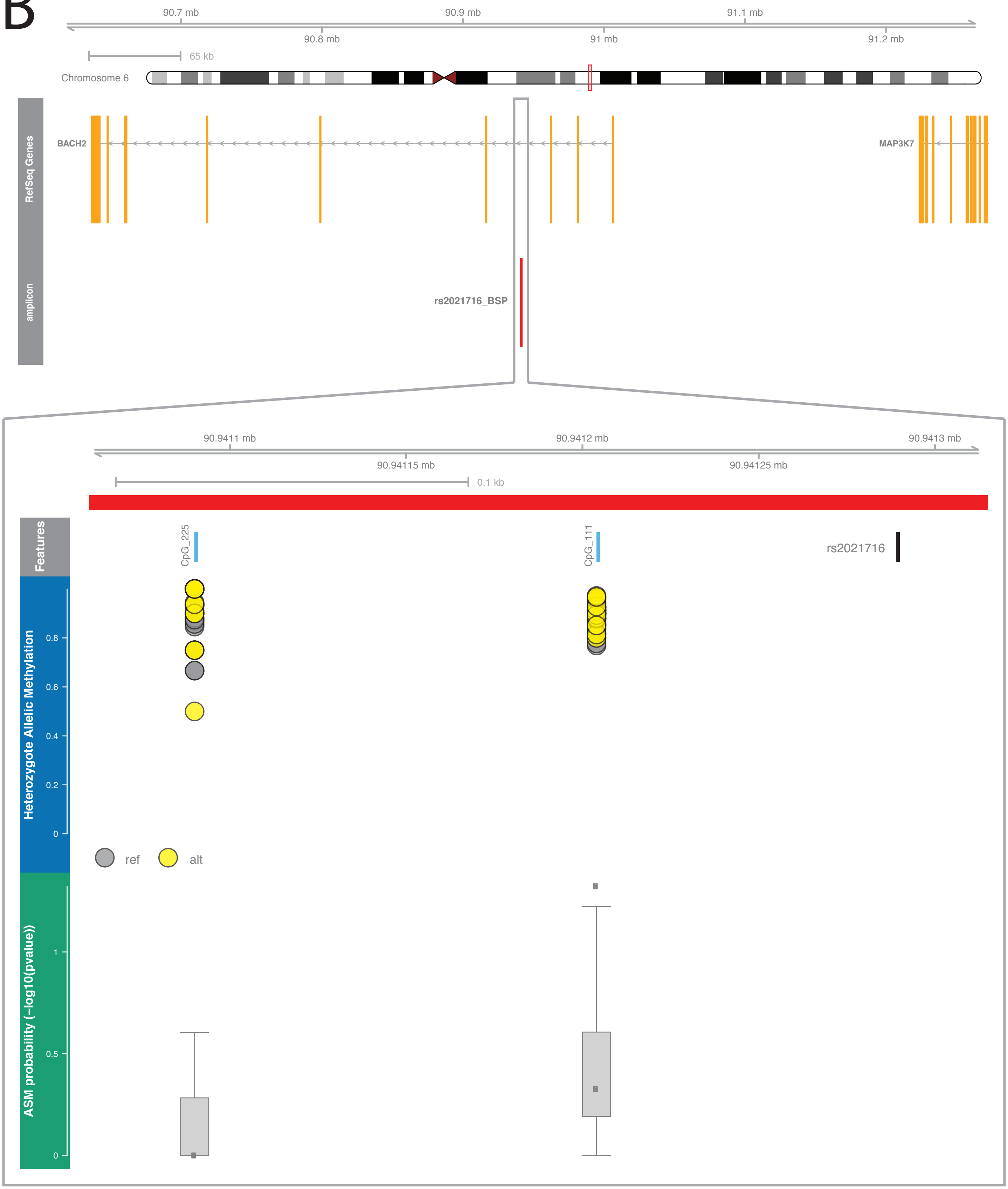

Figure S5 (continued)

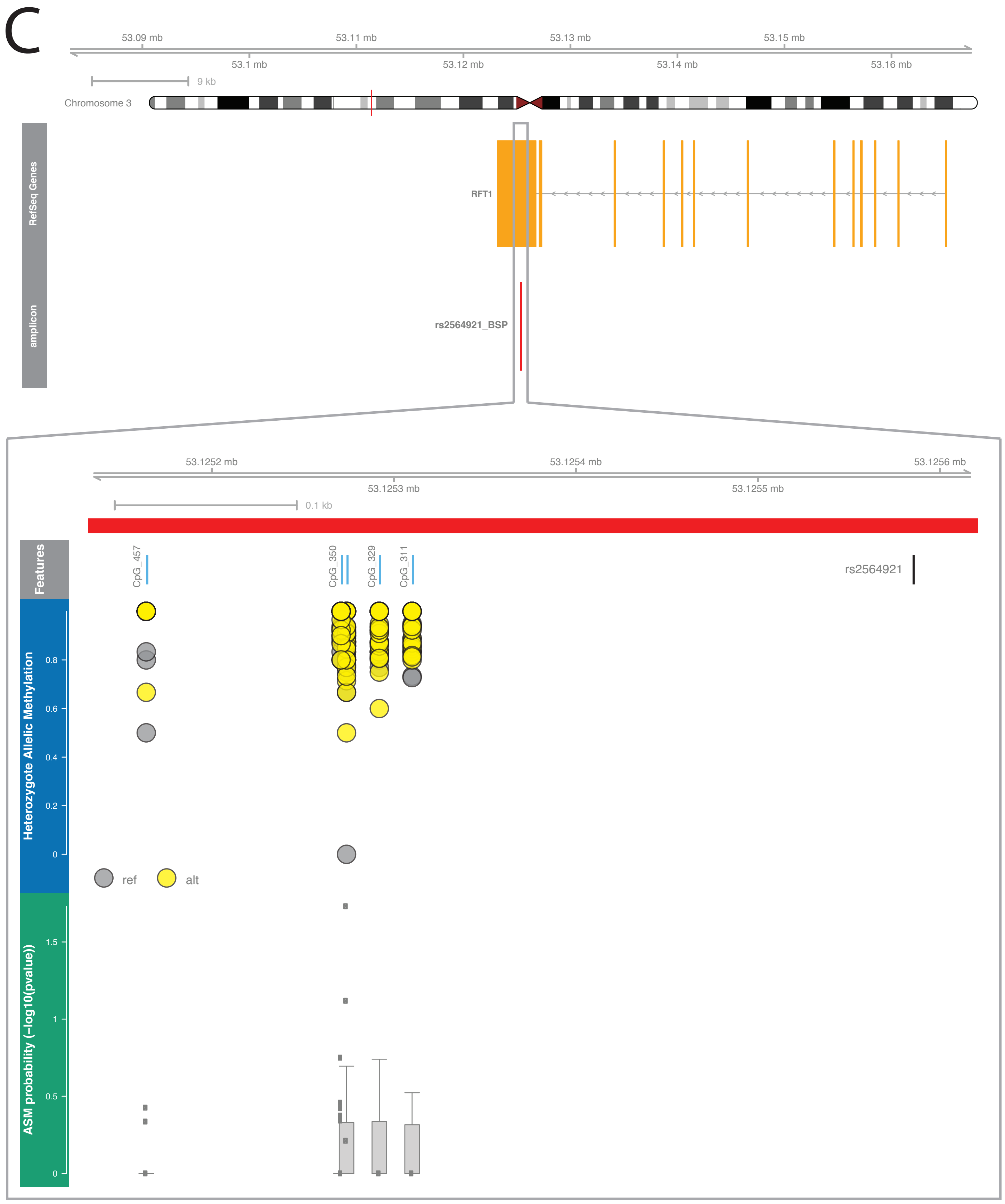

Figure S5 (continued)

D

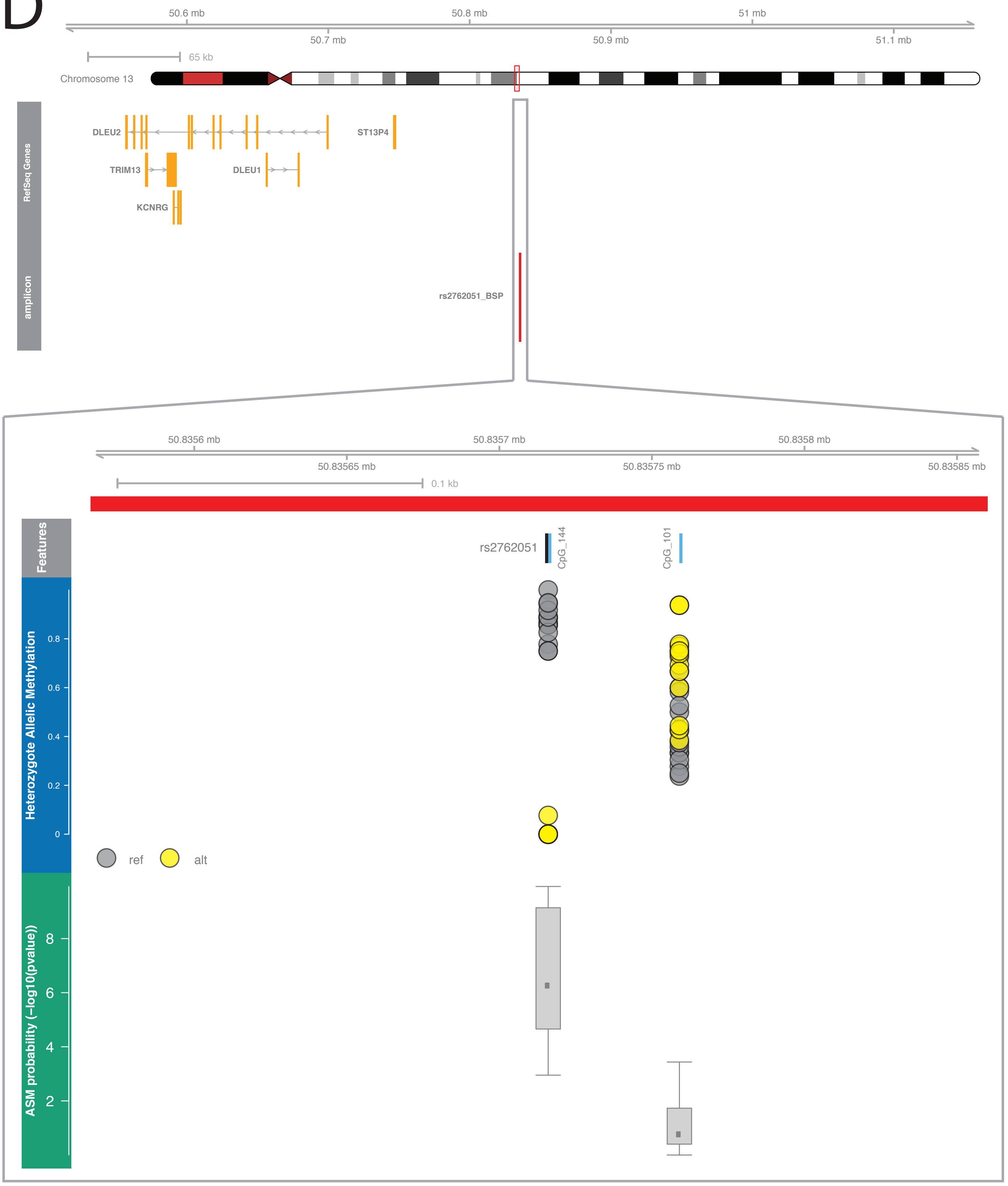

Figure S5 (continued)

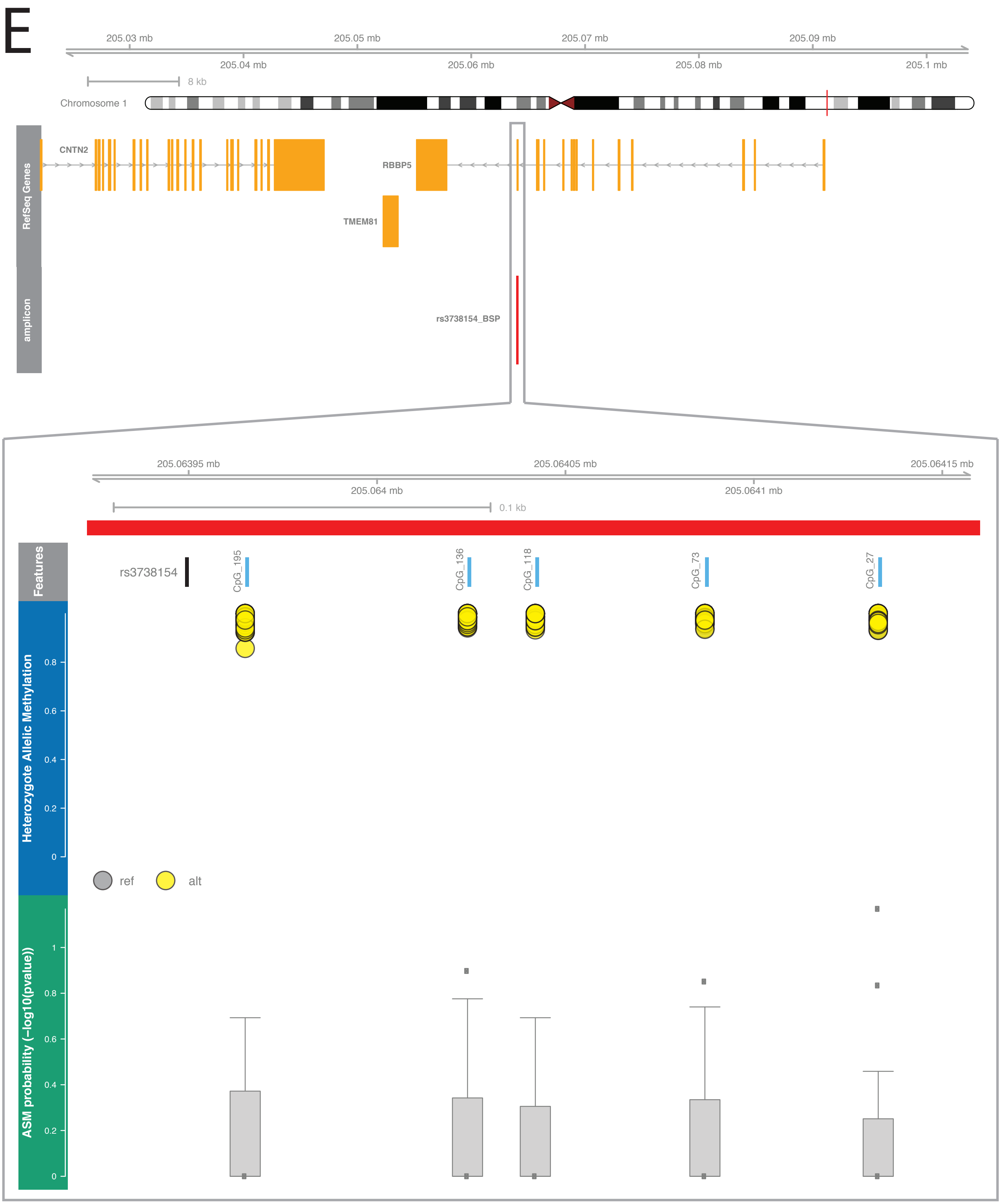

Figure S5 (continued)

F

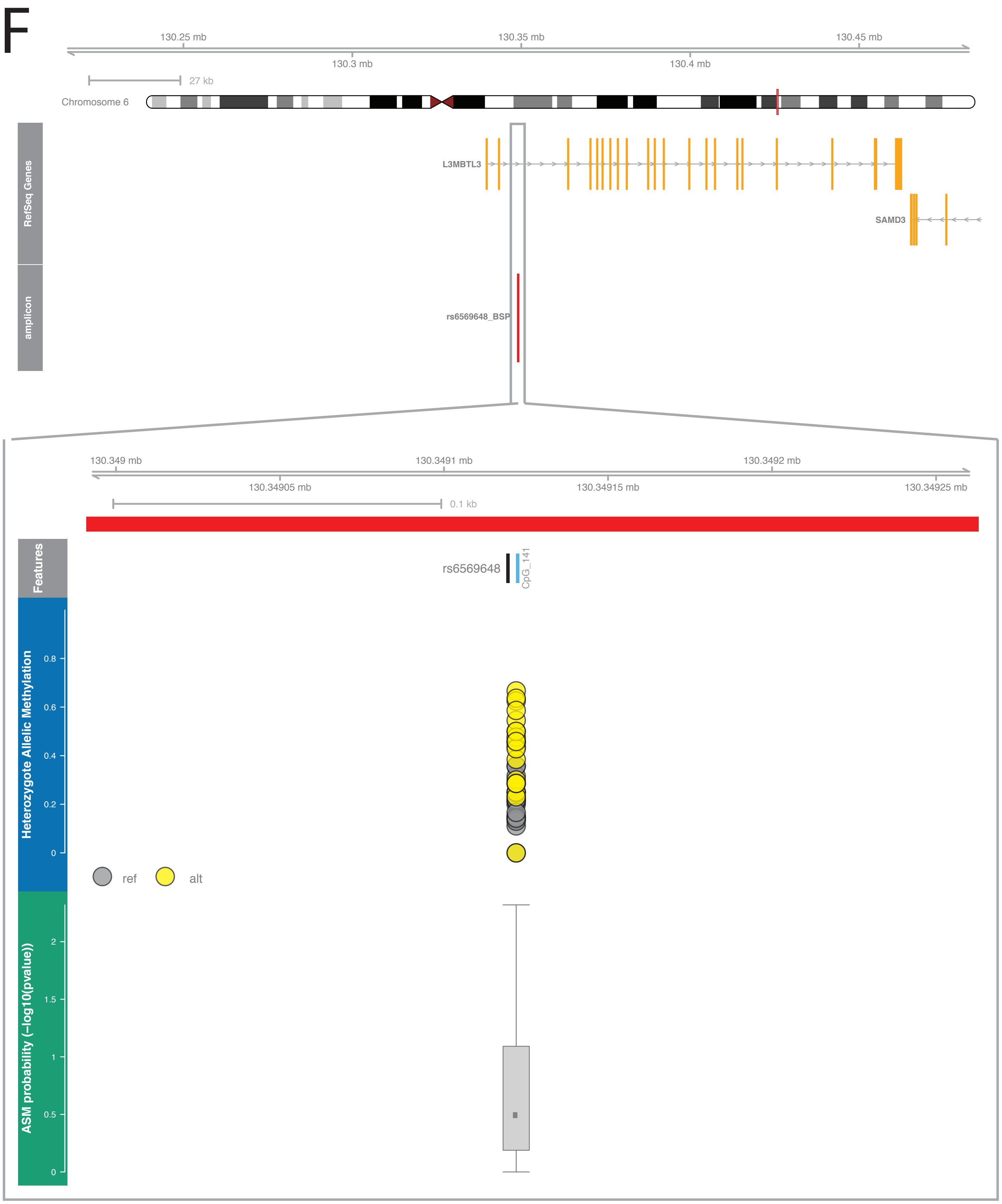

Figure S5 (continued)

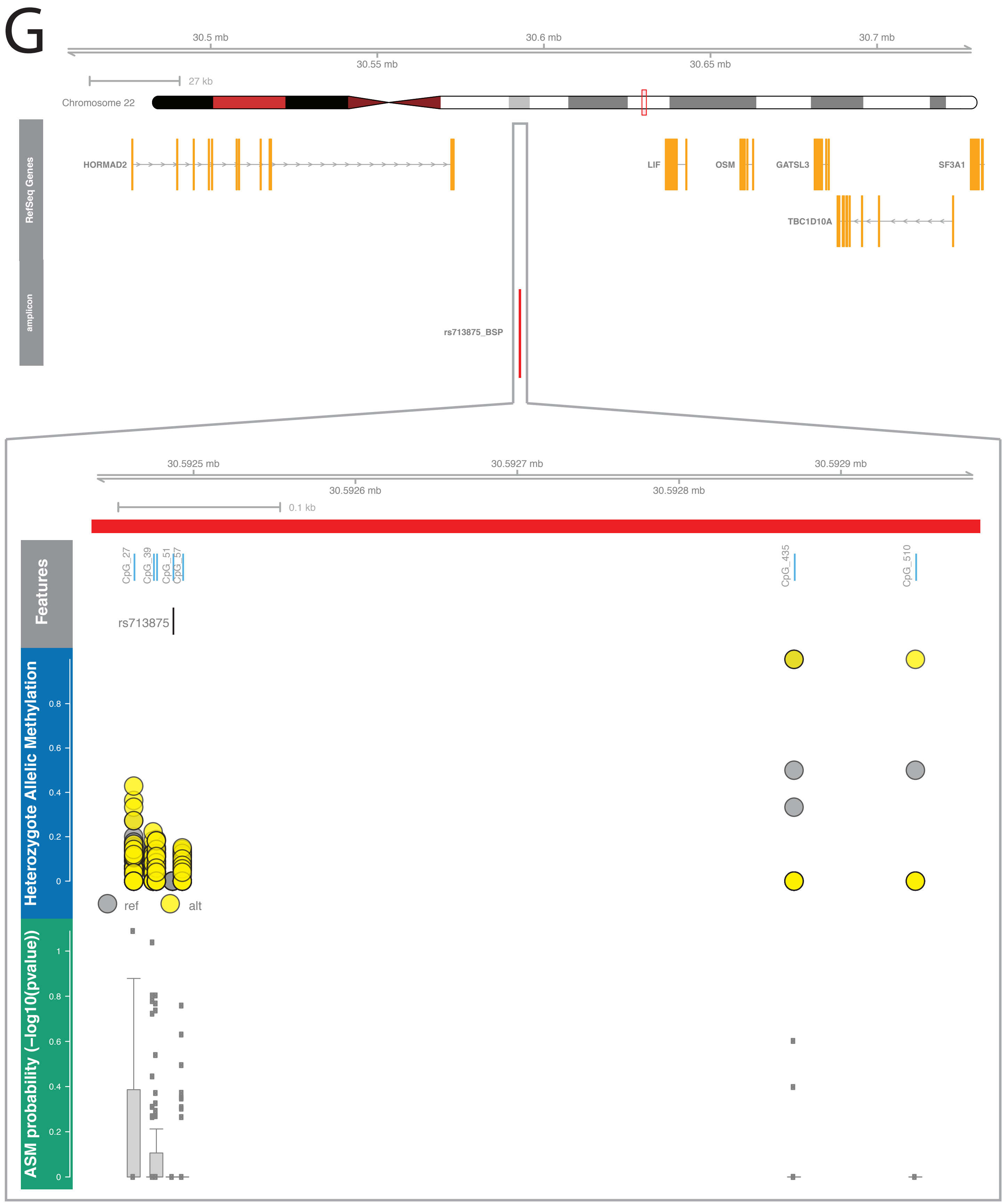

Figure S5 (continued)

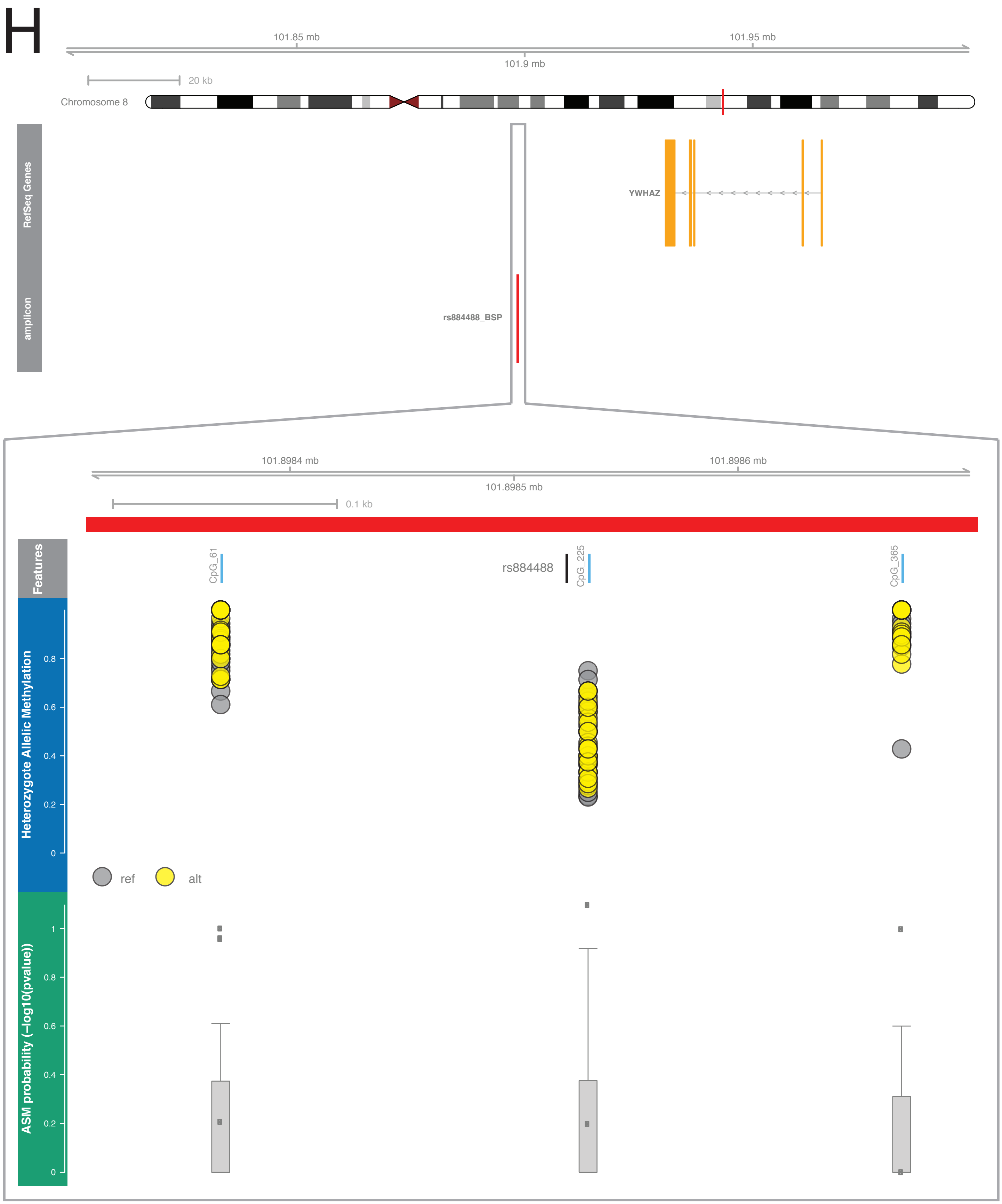

Figure S5 (continued)

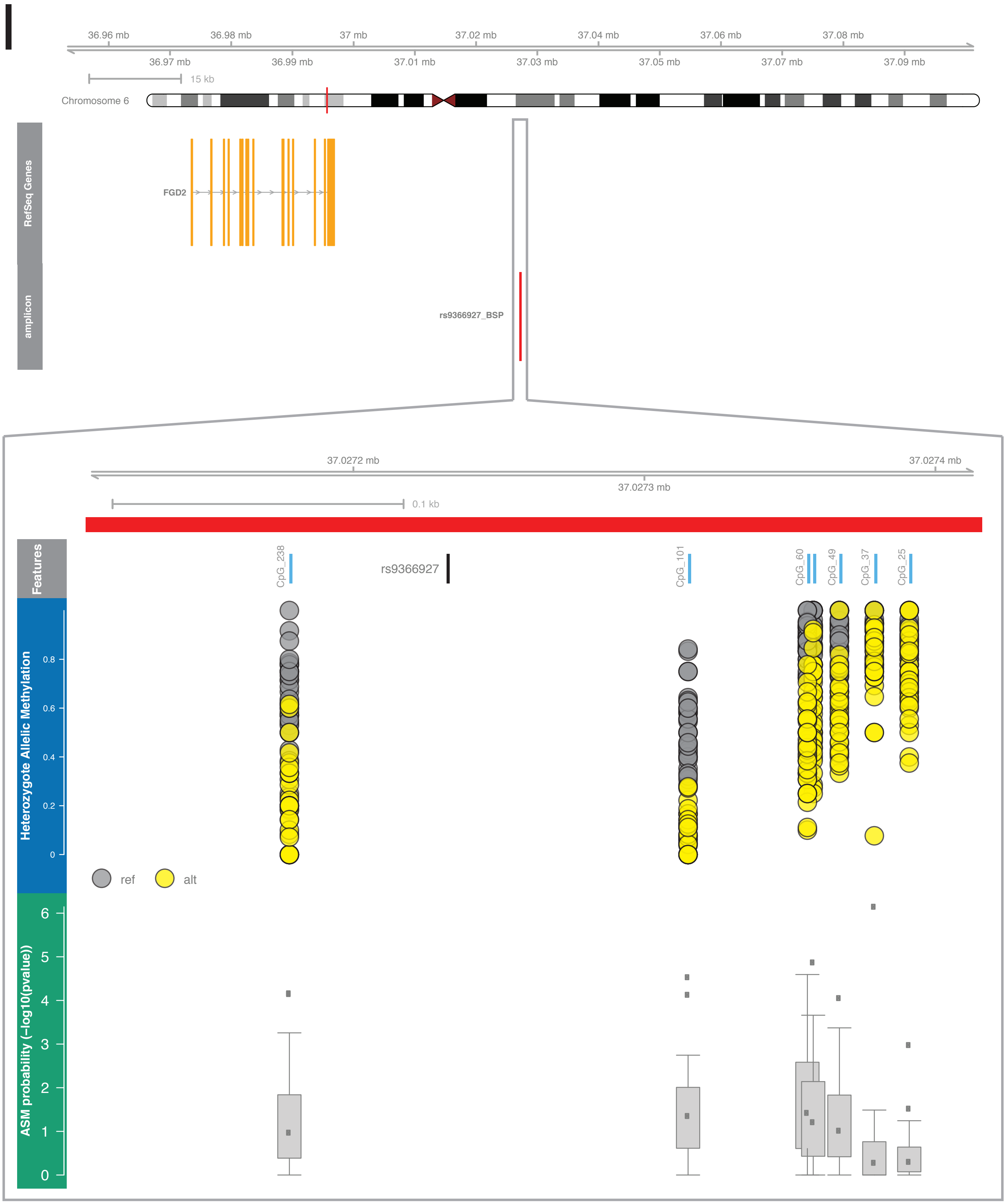

Figure S5 (continued)

J

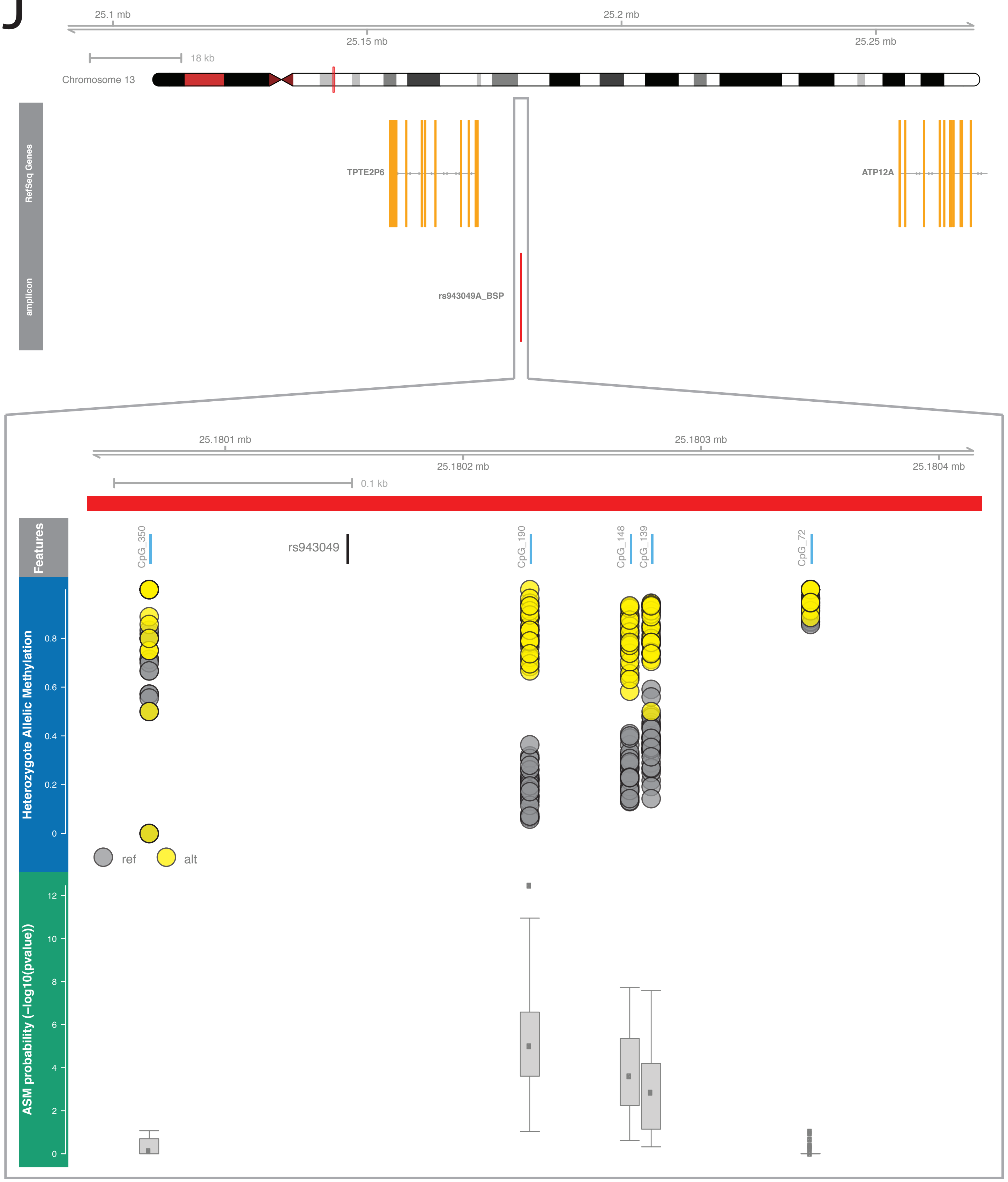

Supplement: Figure S5 — Genomic context of amplicons and methylation events. Illustrations showing genomic context and individual CpG methylation levels for ten amplicons (A - rs10491434_BSP; B - rs2021716_BSP; C - rs2564921_BSP; D - rs2762051_BSP; E - rs3738154_BSP; F - rs6569648_BSP; G - rs713875_BSP; H - rs884488_BSP; I - rs943049A_BSP; J - rs9366927_BSP) are shown. The chromosomal location of each amplicon is demonstrated with an ideogram and the RefSeq genes (orange) surrounding the amplicon (red line) are shown below. A section (grey box) contracts to the amplicon region itself to show the relative positions of the SNPs (black lines) and CpGs (blue lines) within the amplicons themselves (red bars); methylation levels of the alternate (grey circles) and reference (yellow circles) alleles within samples heterozygous for the SNP are graphed below each. The final bottom plot shows, for individual heterozygote samples, the results of chi-square tests of the association between allele state and individual CpG's cytosine methylation states, reported as–log10 transformed p-values. (PDF) [file pone.0098464.s005.pdf]
