# Supplementary material for: Allele-Specific Methylation Occurs at Genetic Variants Associated with Complex Disease
Source: PLoS One. 2014 Jun 9;9(6):e98464. doi: 10.1371/journal.pone.0098464 (PMC4049588; doi:10.1371/journal.pone.0098464)

Figure S6

A

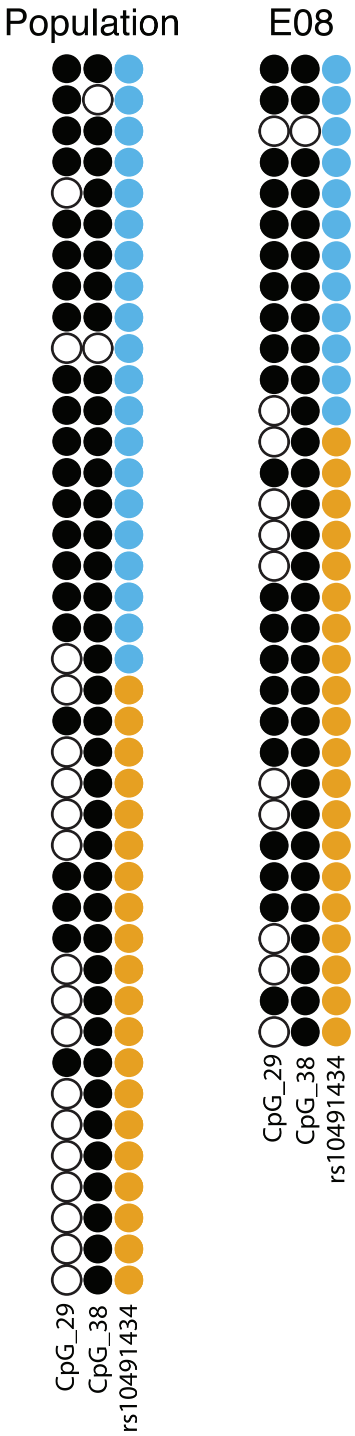

Population
E08
CpG_29
CpG_38
rs10491434
CpG_29
CpG_38
rs10491434

Figure S6 (continued)

B

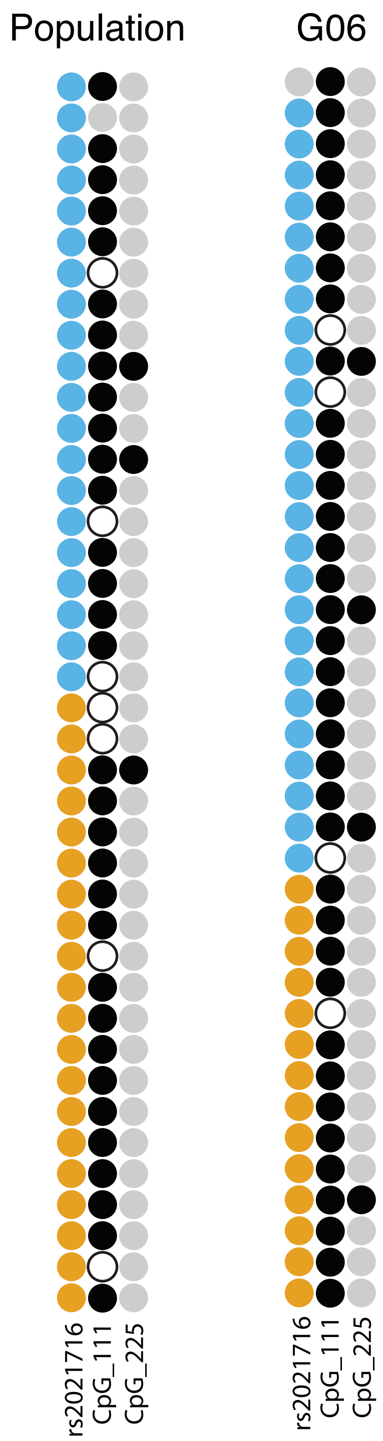

Figure S6 (continued)

C

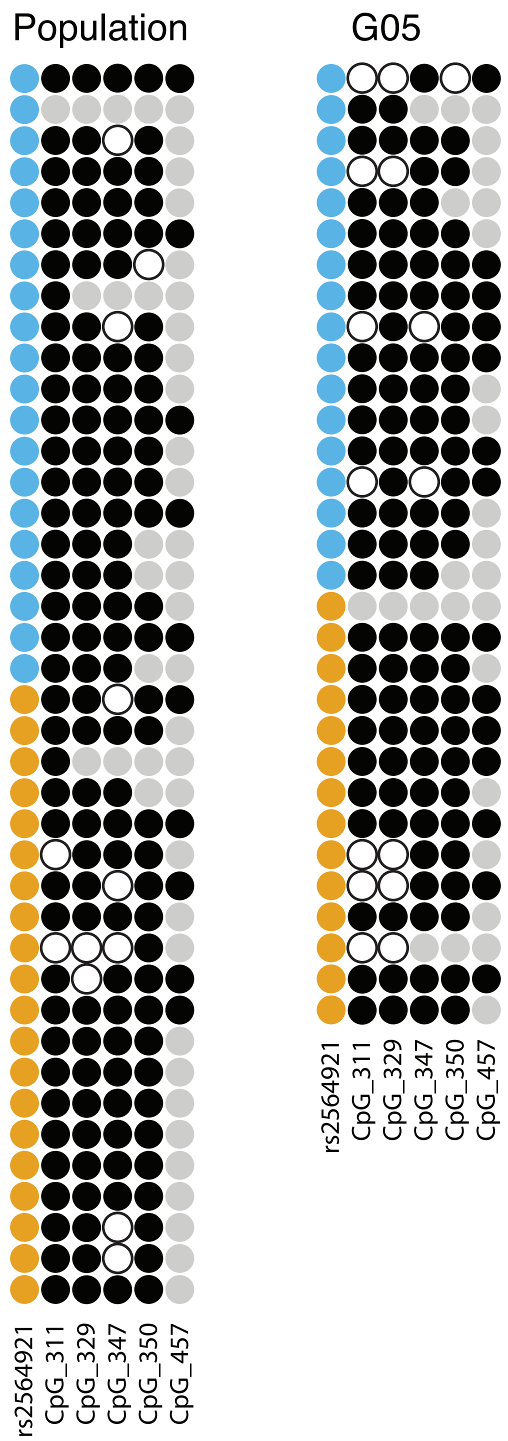

Figure S6 (continued)

D

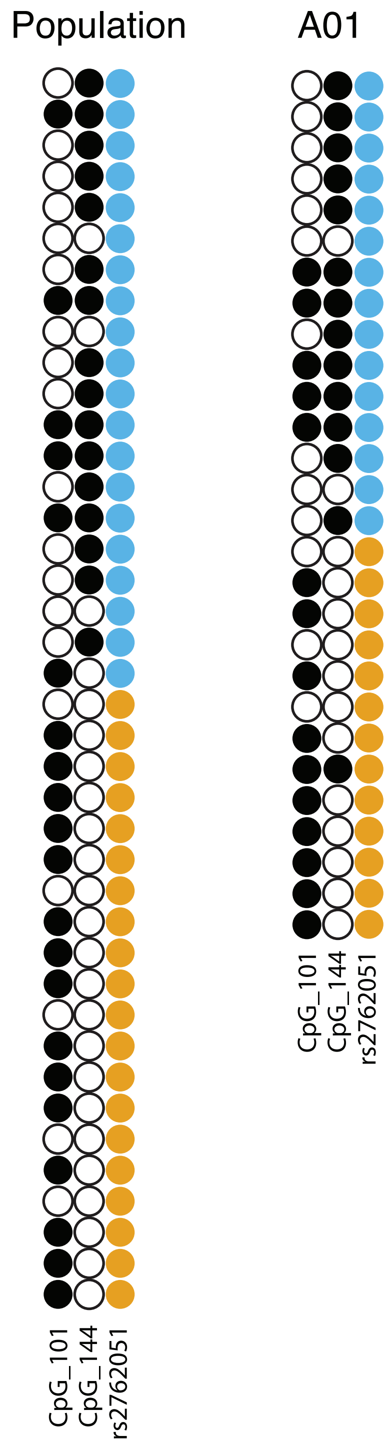

Figure S6 (continued)

E

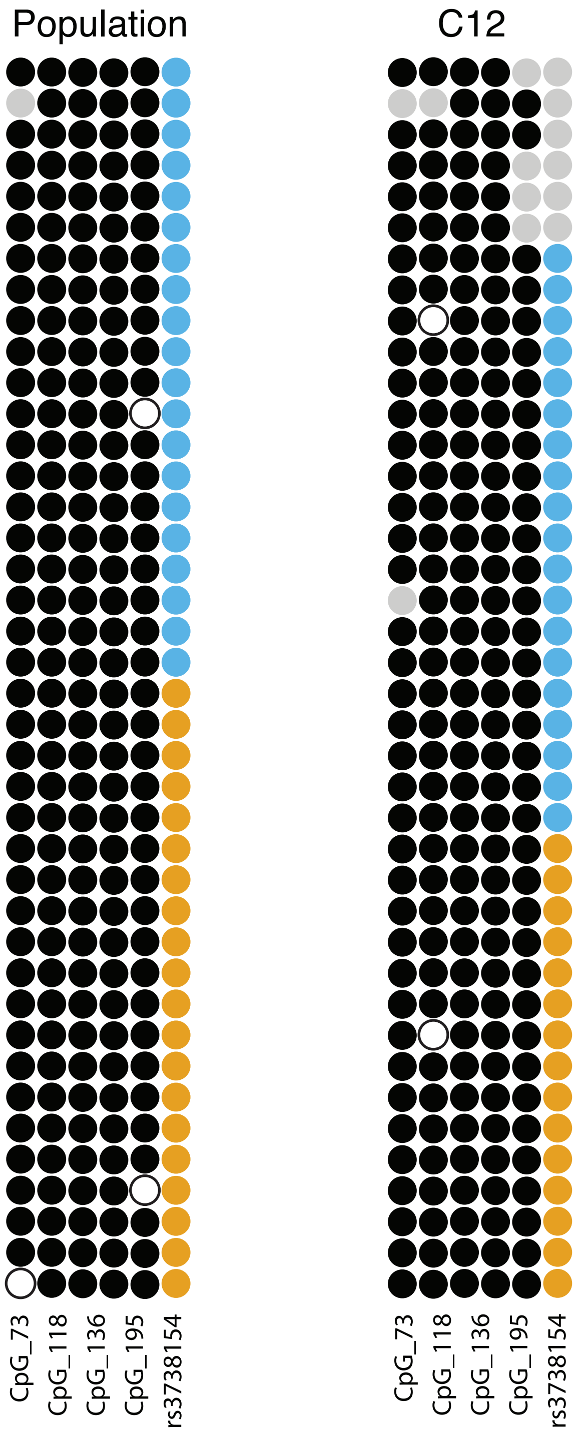

Figure S6 (continued)

F

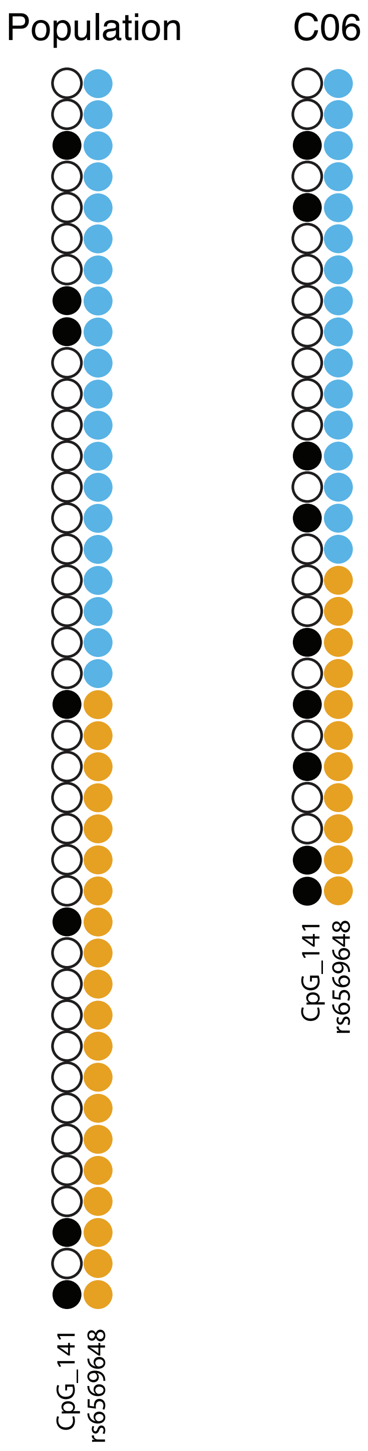

Figure S6 (continued)

G

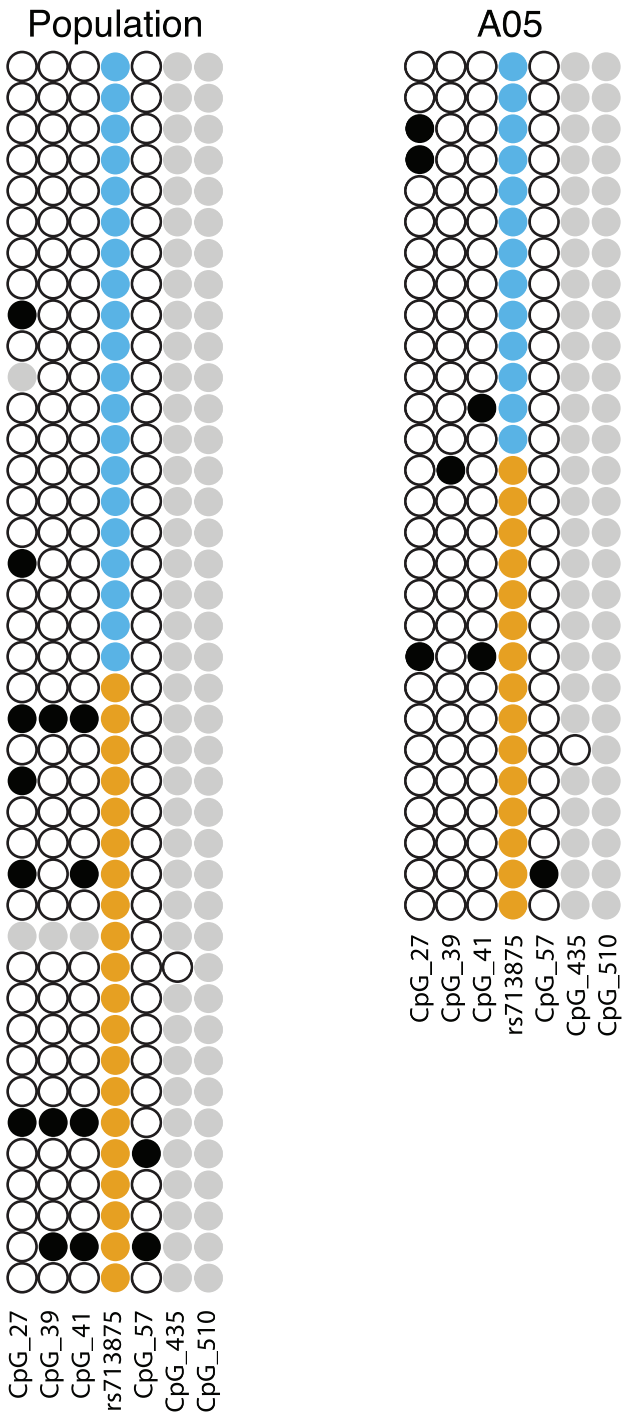

Figure S6 (continued)

H

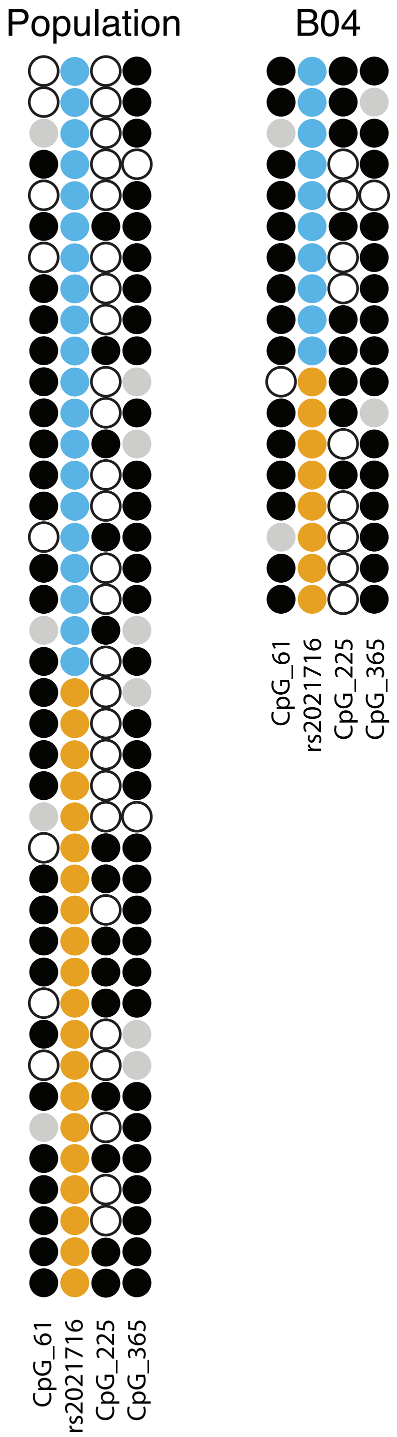

Figure S6 (continued)

I

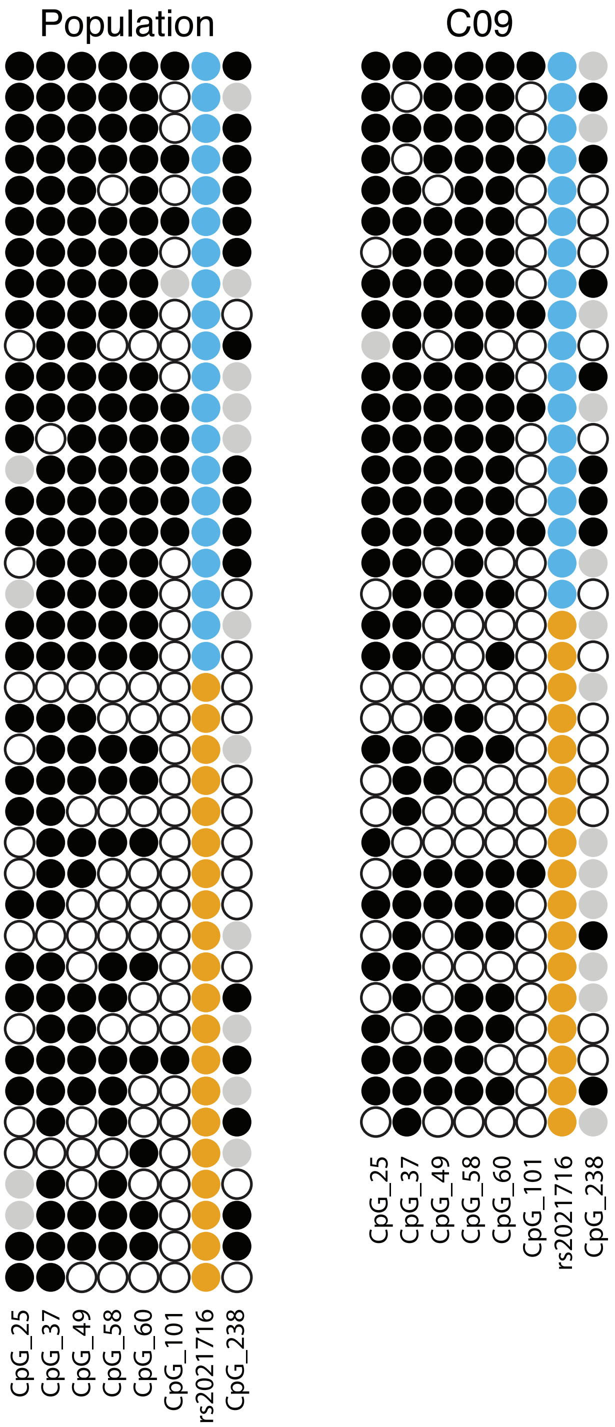

Figure S6 (continued)

J

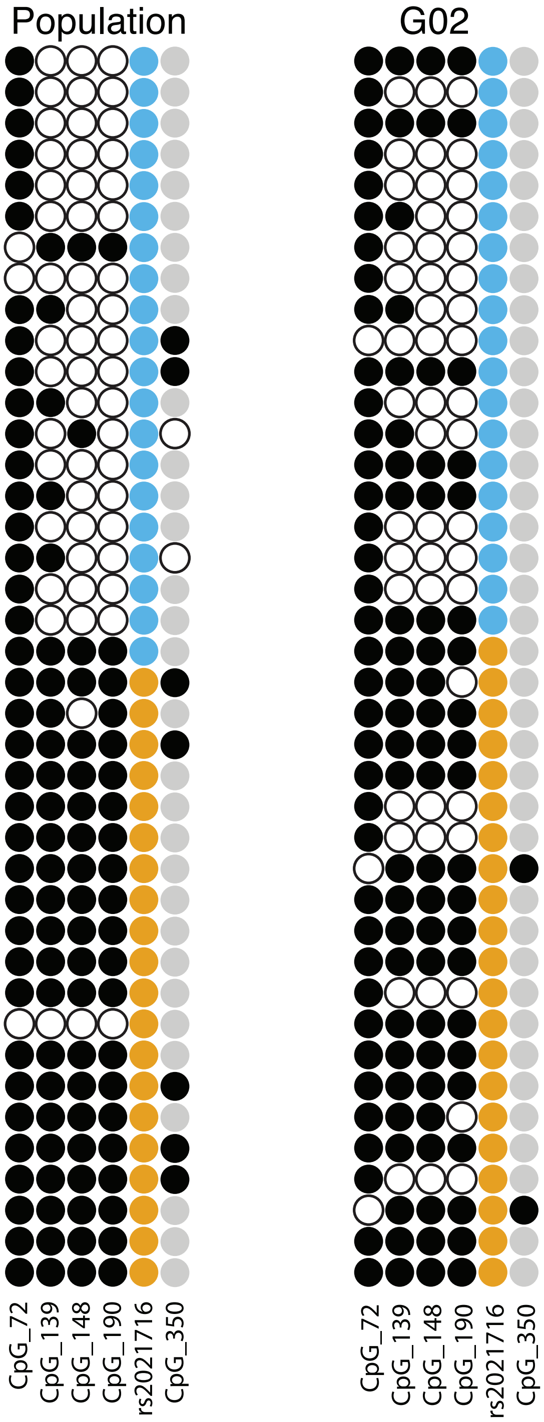

Supplement: Figure S6 — Methylation patterns of amplicons. Illustrations showing methylation patterns for ten amplicons at both a population level and within a representative individual. “Lollipop” plots show both methylation calls for 30 randomly drawn reads from each allele from the entire population (left plot) and for a representative heterozygous individual for that amplicon (right plot). Representative individuals were selected by finding the individual closest to the median chi-square based p-value for the CpG with the highest likelihood of ASM based on lowest mean chi-square based p-value. CpG methylation status is shown in black (methylated) and white (unmethylated) while SNP status is shown in blue (reference allele) and orange (alternate allele). CpGs or SNPs on a read without information (typically due to gaps in the read) are displayed in grey. (PDF) [file pone.0098464.s006.pdf]
