# Supplementary material for: Allele-Specific Methylation Occurs at Genetic Variants Associated with Complex Disease
Source: PLoS One. 2014 Jun 9;9(6):e98464. doi: 10.1371/journal.pone.0098464 (PMC4049588; doi:10.1371/journal.pone.0098464)

Figure S10

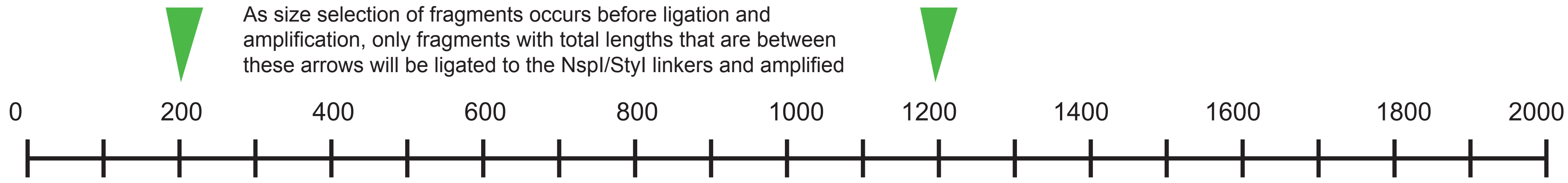

1st alternative

BOTH StyI and NspI fragments must have:
total lengths of 200-1200bp
no known MSRE sites
no known SNPs adding an MSRE site
no known SNPs which would change the number of NspI/StyI sites

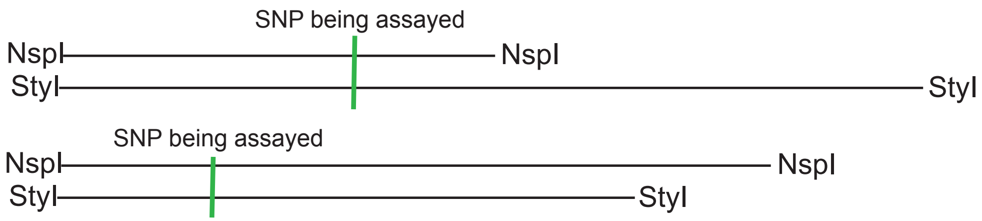

2nd alternative

One fragment must have:
a total length of 200-1200bp
no known MSRE sites
no known SNPs adding an MSRE site
no known SNPs that change the number of NspI/StyI sites

Other fragment may have:
have a total length of more than 1200bp
an MSRE site
SNPs modifying the nuymber of MSRE sites
no known SNPs that change the number of NspI/StyI sites
(even if this fragment is digested by MSREs to be within the 200-1100bp size range, it will not have two NspI/StyI sites available for ligation, so will not be amplified)

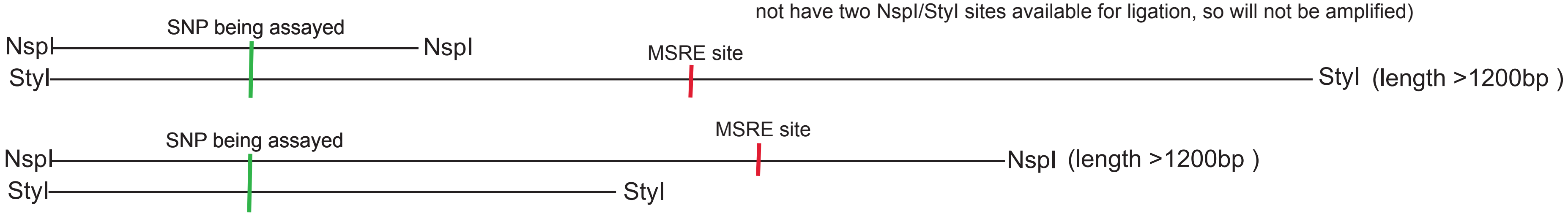

Supplement: Figure S10 — Method of MNR selection. Amplicons were chosen as MNRs based on two criteria: 1) assay based size selection of amplicons results in a final amplicon size range of 200–1200 bp and 2) bioinformatic prediction of MSRE site locations. These criteria allow selection of 3 classes of MNR amplicons expected to show no effect of MSRE digestion: a) those with both NspI and StyI amplicons of 200–1200bp with no MSRE sites (1st alternative) and those with either b) NspI amplicons of 200–1200 bp with no MSRE sites and StyI amplicons outside this size range (2nd alternative, top) or c) StyI amplicons of 200–1200 bp with no MSRE sites and NspI amplicons outside this size range (2nd alternative, bottom). (PDF) [file pone.0098464.s010.pdf]
