# Supplementary material for: Allele-Specific Methylation Occurs at Genetic Variants Associated with Complex Disease
Source: PLoS One. 2014 Jun 9;9(6):e98464. doi: 10.1371/journal.pone.0098464 (PMC4049588; doi:10.1371/journal.pone.0098464)
Supplement: Figure S13 — Assessment of MNRs based quantile interpolation normalization method in control methylation mixes. Scatterplots are shown of the probe A intensity values of un-normalized (left panels) and normalized (right panels) of two replicates from two separate reciprocal 50∶50 E44:H16 control mixes. In these 50∶50 mixes, only one of the samples is methylated; in the top panels, the E44 sample is methylated and the H16 unmethylated, in the bottom panels the H16 sample is methylated and the E44 sample unmethylated. The MNR quantile interpolated normalization based method used greatly reduced variation between replicates. (PDF) [file pone.0098464.s013.pdf]

Figure S13

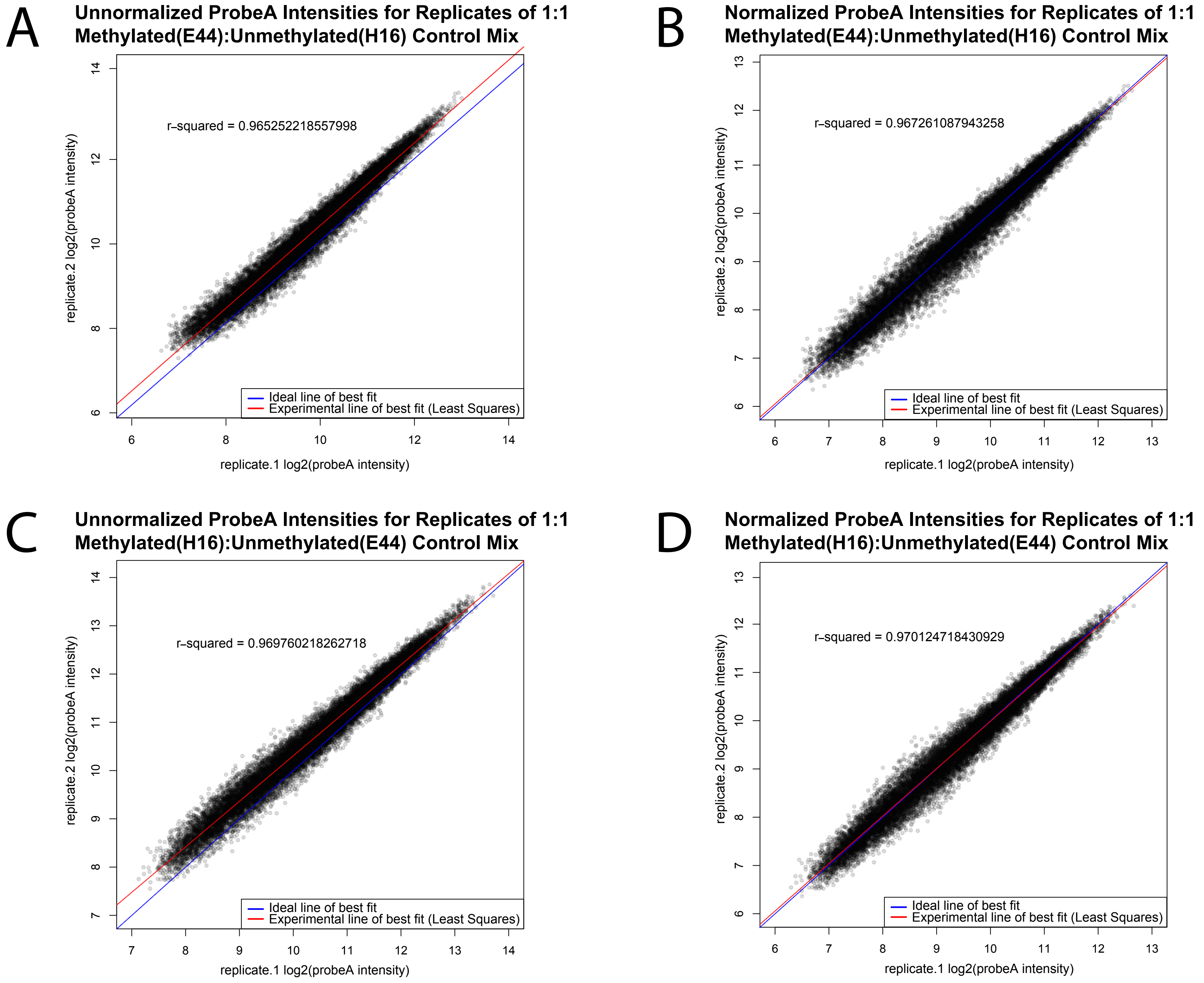
A
Unnormalized ProbeA Intensities for Replicates of 1:1 Methylated(E44):Unmethylated(H16) Control Mix
r–squared = 0.965252218557998
replicate.2 log2(probeA intensity)
replicate.1 log2(probeA intensity)
Ideal line of best fit
Experimental line of best fit (Least Squares)
B
Normalized ProbeA Intensities for Replicates of 1:1 Methylated(E44):Unmethylated(H16) Control Mix
r–squared = 0.967261087943258
replicate.2 log2(probeA intensity)
replicate.1 log2(probeA intensity)
Ideal line of best fit
Experimental line of best fit (Least Squares)
C
Unnormalized ProbeA Intensities for Replicates of 1:1 Methylated(H16):Unmethylated(E44) Control Mix
r–squared = 0.969760218262718
replicate.2 log2(probeA intensity)
replicate.1 log2(probeA intensity)
Ideal line of best fit
Experimental line of best fit (Least Squares)
D
Normalized ProbeA Intensities for Replicates of 1:1 Methylated(H16):Unmethylated(E44) Control Mix
r–squared = 0.970124718430929
replicate.2 log2(probeA intensity)
replicate.1 log2(probeA intensity)
Ideal line of best fit
Experimental line of best fit (Least Squares)
