# Supplementary material for: Allele-Specific Methylation Occurs at Genetic Variants Associated with Complex Disease
Source: PLoS One. 2014 Jun 9;9(6):e98464. doi: 10.1371/journal.pone.0098464 (PMC4049588; doi:10.1371/journal.pone.0098464)
Supplement: Information S1 — Supplementary methods and references. (DOC) [file pone.0098464.s023.doc]

## SUPPLEMENTAL METHODS

### Allele-specific methylation Detection Method Development

Methods to identify allele-specific methylation were developed through the use of control mixes with known amounts of differential methylation.

### Assay Development Control Mixes

To construct samples with known amounts of methylation, we first produced unmethylated replicates of the genomic DNA from clonal lymphoblast lines (LBLs) of two unrelated individuals (E44 (Coriell line GM07057) and H16 (Coriell line GM13130)) by performing whole-genome amplifications. Genomic DNA (10 ng) was amplified using the whole genome amplification phi29 kit (GE Healthcare) according to the manufacturer’s manual. The DNA was split into two aliquots and one aliquot methylated with the CpG methyltransferase M.SssI (NEB). Methylated and unmethylated DNAs from the two individuals were then mixed together at various ratios to produce samples with known amounts of methylation. Further, artificial heterozygotes (AABB/BBAA) in these mixes were formed at SNPs homozygous for opposite alleles (i.e. E44-AA and H16-BB) in the two individuals.

### Methylation Analyses - Affymetrix Array Data Extraction and Analysis

The median value of the representative allele probe sets was used to summarize allele probe intensities. Traditional normalizations were carried out using the Affymetrix powertools apt-probeset-summarize module . Our variation of invariant probeset normalization was achieved using a combination of custom Perl and R scripts, with the quantile normalization step carried out by the normalize.quantiles.robust module of the affy package in Bioconductor . In order to ensure constancy of quantile adjustments, the HapMap sample NA06985_C_F3 was used as a weighted model for all normalizations.

Conventional Affymetrix 6.0 genotyping was carried out by Birdseed v1.2 using default values. Arrays were run individually and dynamic modeling of clusters disabled i.e. genotypes were called from clusters derived from a reference model based on the 270 HapMap samples. Samples used were derived from GM10849 and GM12093 as previously described .

### Methylation Analyses - Allele-specific methylation Calling

Previous experiments within the Chess lab relied on the Affymetrix 500K platform and used conventional Affymetrix 500K genotyping methods for detection of allele-specific methylation . Using the Affymetrix 6.0 platform allowed us access to twice as many SNPs, but required the development and evaluation of new methods. These methods consisted of two main steps; normalization and differential methylation detection.

We attempted to remove variation between arrays of non-biological origin by the process of normalization. Initially, we examined conventional normalization methods for Affymetrix 6.0 chips i.e. median or quantile normalization of all probesets. We studied these methods using two of the most extreme sample types we could expect to encounter in an experiment i.e. a sample in which every MSRE site is unprotected from MSRE digest (unmethylated DNA + MSRE digestion) and a sample in which every site is unaffected by MSRE digestion (unmethylated DNA without MSRE digestion). A robust normalization method should be able to adjust for overall technical variation but leave true biological variation, with the MSRE digested sample distribution skewed towards lower intensities (from MSRE digested probesets) in comparison to the undigested sample, with similar high intensity ranges in the two samples (from any undigested probesets lacking MSRE sites).

When adjusting probe intensities by median normalization of all probesets (to make the overall median equal between these two samples) we found that the post-normalization MSRE digested unmethylated control mix sample ultimately had more probesets with higher intensities than the undigested post-normalization sample (Figure S9), a result that does not reflect the underlying biology. These results are likely due to the presence of a large number of probesets with low to no signal (from MSRE digested amplicons) in the un-normalized MSRE digested sample. The presence of these probesets skews the median low; subsequent equalization of the sample medians would result in excessive amplification of undigested probeset intensities. In contrast, quantile normalization results in inappropriate amplification of digested probeset intensities so that the post-normalization MSRE digested sample no longer has probesets with lower intensities than the undigested sample, a result that is also unreflective of the underlying biology. This result is due to quantile normalization methods assuming that samples share the same underlying distribution, an unwarranted assumption in any case where samples differ in their overall methylation levels.

To overcome these challenges, we developed methods tailored for the analysis of MSRE treated samples which took advantage of the fact that many amplicons lack MSRE sites and are expected to be unaffected by MSRE digestion. Specifically, probe intensities from these subsets of MSRE Negative Regions (MNRs) were quantile normalized. Normalization intensity adjustments for each MPR probe were taken from those of the MNR of closest pre-normalization intensity. The identities of MNRs were determined from the consensus human genome sequence and only amplicons with no MSRE sites or modifying SNPs from dbSNP129 were permitted in the set of MNRs (Figure S10, Table S4). As the Affymetrix 6.0 platform does not separately hybridize the NspI and StyI fragment amplicons, we further filtered the MNRs to eliminate any SNPs assayed by a StyI based target amplicon with at least one MSRE site and an NspI-based target amplicon without an MSRE site (or vice versa). To validate this approach we examined the effect of MSRE digestion on MNRs and MPRs (the latter containing at least one of each of the MSRE sites) in artificially methylated control mixes. While there was little difference in the intensity distributions of MNRs to MPRs in an unmethylated control sample before MSRE digestion, MPRs showed greatly lowered combined probe intensities relative to the MNRs after MSRE digestion (Figure S11). Similarly, in the un-normalized data from control mixes with a 1:1 mix of unmethylated DNA from one individual and methylated DNA from the other, the A allele frequency was largely constant regardless of the A allele methylation status. In contrast, MPRs showed lower A allele frequencies when the A allele was unmethylated (Figure S12). The effects of this normalization process are illustrated for various control mixes (Figure S13).

Previous experiments utilizing the Affymetrix 500K platform detected allele-specific methylation as a genotype change from "AB" in undigested genomic DNA to an "AA" or "BB" genotype in the same sample after MSRE treatment. We initially explored the potential to use conventional Affymetrix 6.0 genotyping methods such as Birdseed , which relies on a model-based clustering algorithm to assign SNPs to one of the 3 genotypes, to perform similar analyses with our MSRE treated samples. To do so, we examined the genotyping results from identical samples before and after MSRE treatment, which were run on the Affymetrix 500K (genotyped as previously described ) and Affymetrix 6.0 platforms (genotyped with the Birdseed algorithm after normalization as detailed above). Comparison of genotypes from these two approaches showed discordant genotypes for up to 15% of the SNPs common between platforms in undigested samples. In contrast, in MSRE treated samples, the genotypes were discordant for up to 40% of the common SNPs. In particular, SNPs labeled NoCall by the 500K platform (mainly representing SNPs with biallelic lack of methylation) were always called by the Birdseed algorithm and accounted up to two-thirds of the discordance in MSRE treated samples while accounting for less than 20% of the original 500K calls. These results suggest that SNPs associated with biallelic lack of methylation were wrongly assigned to genotype clusters in our MSRE treated samples. Birdseed can use a confidence score to exclude SNPs, but we were unable to use these to identify such SNPs from true genotype calls as examination of these confidence scores revealed no difference between the distribution of confidence scores for SNPs with matching calls and those SNPs with mismatching calls between platforms (data not shown). These results indicated that the conventional clustering based methods of genotyping Affymetrix 6.0 could not be easily adapted to the detection of allele-specific methylation in MSRE treated samples.

We developed a custom method to detect allele-specific methylation in MSRE treated samples based on standard normalized changes (Standard Scores) in the log2 relative allele (probeA/probeB) frequency (RAF) for AB genotypes after MSRE digestion derived from a reference set composed of 270 samples from the International HapMap project (Figure 1). Untreated samples were initially genotyped with Birdseed. In MSRE treated samples, SNPs with less than 20% combined probe intensity (
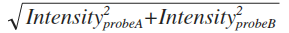
) relative to the average combined probe intensity observed in the reference HapMap dataset were removed from analysis, as these SNPs should represent biallelically unmethylated SNPs for which the allelic probe intensities represent only non-specific hybridization (Figure S8). For each SNP, a distribution of log2 (A/B) values was derived from 270 undigested heterozygous HapMap samples, and all MSRE-treated heterozygous sample SNPs standard normalized on a SNP-by-SNP basis to obtain a Standard Score. To make allele-specific methylation calls, Standard Score cutoffs were chosen that excluded 95% of the MNR Standard Score values, any SNP with a Standard Score value more extreme than these empirically derived sample-specific cutoffs was called as either A (A allele methylated) or B (B allele methylated), depending on the direction of deviation from the mean. To ensure that changes from AB states were reliably detected, SNPs showing poor separation of genotypes within the reference data set as determined from RAF values (i.e. SNPs where the reference set AA RAF value was less than 3 standard deviations from the AB mean) were discarded from all analyses.

REFERENCES
